# Comprehensive transcriptional variability analysis reveals gene networks regulating seed oil content of *Brassica napus*

**DOI:** 10.1186/s13059-022-02801-z

**Published:** 2022-11-07

**Authors:** Zengdong Tan, Yan Peng, Yao Xiong, Feng Xiong, Yuting Zhang, Ning Guo, Zhuo Tu, Zhanxiang Zong, Xiaokun Wu, Jiang Ye, Chunjiao Xia, Tao Zhu, Yinmeng Liu, Hongxiang Lou, Dongxu Liu, Shaoping Lu, Xuan Yao, Kede Liu, Rod J. Snowdon, Agnieszka A. Golicz, Weibo Xie, Liang Guo, Hu Zhao

**Affiliations:** 1 National Key Laboratory of Crop Genetic Improvement, Huazhong Agricultural University, Wuhan, China; 2 Hubei Hongshan Laboratory, Wuhan, China; 3 Department of Plant Breeding, Justus Liebig University, Giessen, Germany; 4 Hubei Key Laboratory of Agricultural Bioinformatics, College of Informatics, Huazhong Agricultural University, Wuhan, China; 5 Shenzhen Institute of Nutrition and Health, Huazhong Agricultural University, Wuhan, China; 6 Shenzhen Branch, Guangdong Laboratory for Lingnan Modern Agriculture, Genome Analysis Laboratory of the Ministry of Agriculture, Agricultural Genomics Institute at Shenzhen, Chinese Academy of Agricultural Sciences, Shenzhen, China

**Keywords:** *Brassica napus*, eQTL, Subgenome, Machine learning, Regulatory network, Seed oil content

## Abstract

**Background:**

Regulation of gene expression plays an essential role in controlling the phenotypes of plants. *Brassica napus* (*B. napus*) is an important source for the vegetable oil in the world, and the seed oil content is an important trait of *B. napus*.

**Results:**

We perform a comprehensive analysis of the transcriptional variability in the seeds of *B. napus* at two developmental stages, 20 and 40 days after flowering (DAF). We detect 53,759 and 53,550 independent expression quantitative trait loci (eQTLs) for 79,605 and 76,713 expressed genes at 20 and 40 DAF, respectively. Among them, the local eQTLs are mapped to the adjacent genes more frequently. The adjacent gene pairs are regulated by local eQTLs with the same open chromatin state and show a stronger mode of expression piggybacking. Inter-subgenomic analysis indicates that there is a feedback regulation for the homoeologous gene pairs to maintain partial expression dosage. We also identify 141 eQTL hotspots and find that hotspot87-88 co-localizes with a QTL for the seed oil content. To further resolve the regulatory network of this eQTL hotspot, we construct the XGBoost model using 856 RNA-seq datasets and the Basenji model using 59 ATAC-seq datasets. Using these two models, we predict the mechanisms affecting the seed oil content regulated by hotspot87-88 and experimentally validate that the transcription factors, NAC13 and SCL31, positively regulate the seed oil content.

**Conclusions:**

We comprehensively characterize the gene regulatory features in the seeds of *B. napus* and reveal the gene networks regulating the seed oil content of *B. napus*.

**Supplementary Information:**

The online version contains supplementary material available at 10.1186/s13059-022-02801-z.

## Background

Genetic variants affect gene expression and thereby impact multiple phenotypes [1–3]. Expression quantitative trait locus (eQTL) studies associate genomic variations and transcriptomic datasets and have been widely used to reveal the molecular mechanisms of target trait-associated genomic variations [4]. In recent years, this approach has also played an important role in the resolution of key genes and regulatory networks for plant traits, such as resolving that *Abscisic acid 8′-hydroxylase* plays a negative role in maize drought tolerance [5], *GhHRK1* is a negative regulator in cotton high-temperature stress [6], and *Aradu.10025440* was identified as a novel gene that may control the peanut purple testa [7]. In addition, imbalanced regulation is prevalent among subgenomes of polyploid crops [8–15]. The overall gene expression of the cotton *Dn* subgenome was stronger than the *An* subgenome, and the bias of DNA methylation levels can influence the expression bias of homologous gene pairs in the subgenomes [16, 17]. Moreover, *B. napus* gene expression levels and various active epigenetic signal modifications were significantly stronger on the *An* subgenome than on the *Cn* subgenome, with a higher proportion of SNPs also observed on the *An* subgenome [11, 18]. eQTL is also a good approach to explore the characteristics and regulatory mechanisms among subgenomes of polyploid plants [19–21]. In cotton, for example, eQTL analysis revealed the imbalanced genetic regulation patterns between *An* and *Dn* subgenomes and indicated the important role of the *Dn* subgenome in fiber development [19].

eQTL mapping is a potentially powerful method for revealing gene regulatory relationships. However, the identification of gene regulatory relationships directly from eQTL remains challenging [22]. On the one hand, there are often dozens or hundreds of candidate genes in an eQTL interval and it is difficult to determine which gene plays a regulatory role [23, 24]. On the other hand, significant variants do not definitely represent regulatory variants (RVs), and uncovering regulatory elements (REs) and RVs from numerous significant variants is still very difficult at the present stage [25, 26]. eQTLs combined with machine learning can assist to investigate the causal interactions across genes [27–29]. A previous study used a large amount of transcriptome data to construct XGBoost models to predict gene regulatory networks (GRNs) and combined eQTL information to find the key transcription factors (TFs) in different GRNs [30]. Furthermore, REs are enriched in open chromatin regions (OCRs) and the identification of REs in OCRs as well as high-impact variants can help identify RVs in eQTL study [31, 32]. Assay for transposase-accessible chromatin sequencing (ATAC-seq) can provide insights into genomic patterns of open chromatin [33]. Deep learning network-based models can automatically extract complex features from genomic sequences to accurately and efficiently learn the OCR information and predict the RVs in non-coding regions [34–38]. The development of these methods has greatly facilitated the identification of regulatory relationships in eQTL studies. More importantly, examination of how RVs affect gene expression can help bridge the gap between genomic variations and phenotype.

As an important oil crop, improvement of seed oil content (SOC) is one of the major breeding goals for *Brassica napus* (AACC, 2n = 38) [39]. As we know, there is a complex network of transcriptional regulation in plant lipid metabolism [40, 41]. Among them, several TFs such as *WRINKLED1* (*WRI1*), *LEAFY COTYLEDON2* (*LEC2*), *FUSCA3* (*FUS3*), *ABSCISIC ACID-INSENSITIVE 3* (*ABI3*), *LEAFY COTYLEDON1* (*LEC1*), *GLABRA2* (*GL2*), and *TRANSPARENT TESTA2* (*TT2*) were shown to play important functions in the regulation of seed oil content [42–49]. As an allotetraploid oil crop, *B. napus* is formed by natural hybridization between *Brassica rapa* (AA, 2n = 20) and *Brassica oleracea* (CC, 2n = 18). And with the development of whole-genome sequencing technology, a series of QTLs and genes regulating SOC have been identified in *B. napus* using the genome-wide association study (GWAS) and transcriptome-wide association study (TWAS) [50, 51]. There are complex regulatory networks between subgenomes [52, 53], previous studies lacked the analysis of regulatory networks using the omics data at the population level, and prediction methods of regulatory networks also need to be further developed in the face of new high-throughput sequencing technologies. eQTL mapping from rapeseed population, epigenetic landscapes constructed by ATAC-seq [54], and the well-established machine learning prediction models can help explore the key TFs and corresponding gene regulatory networks involved in seed oil synthesis.

Here, we applied the genomic data of 505 *B. napus* accessions and the corresponding 583 transcriptomes for eQTL identification. We characterized the gene regulatory features between two seed developmental stages and subgenomes. To further obtain accurate regulatory relationships and regulatory elements, we also constructed XGBoost and Basenji models using 856 ZS11 RNA-seq data and 59 ATAC-seq data to predict the key TFs and regulatory mechanisms affecting SOC. Finally, combining the eQTL mapping and the constructed machine learning models, we successfully predicted 141 regulatory hotspots and identified key transcription factors NAC13 and SCL31 impacting SOC and their corresponding regulatory network. Meanwhile, in order to facilitate researchers to query and use this resource, we constructed the Bn-eQTL database (http://yanglab.hzau.edu.cn/BneQTL/), from which the regulatory relationships can be easily accessed (Additional file 1: Fig. S1).

## Results

### Static and dynamic eQTLs underpin regulatory landscapes at two stages of seed development in *B. napus*

**Fig. 1:**
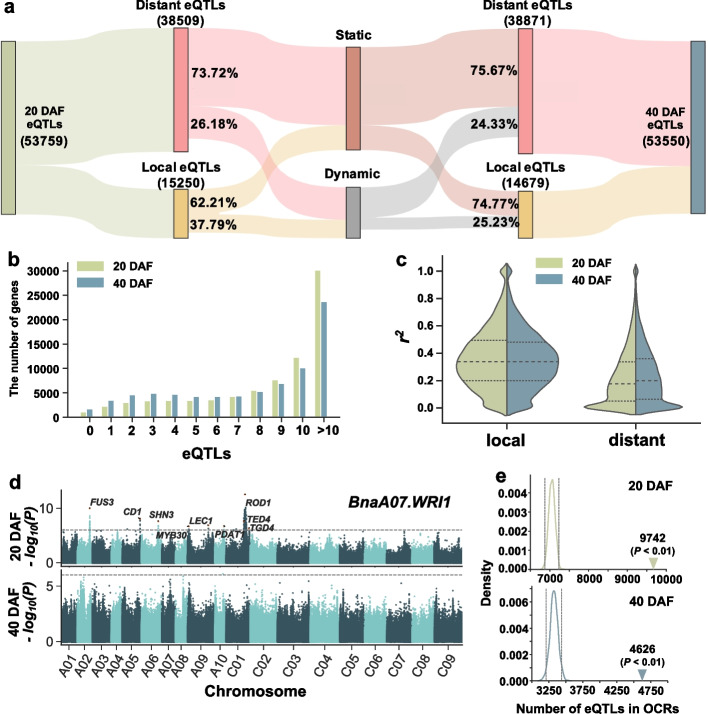
Genome-wide eQTL dynamic changes and characterization. **a** The proportion of static and dynamic distribution of genome-wide local and distant eQTLs at 20 and 40 DAF. **b** The number of eQTLs for each gene. The *x*-axis and *y*-axis represent the number of eQTLs mapped for each gene and the number of genes in each group, respectively. **c** Comparison of the explained variance (*r*^2^) of local and distant eQTLs for expression at 20 and 40 DAF. **d** Manhattan plot of *BnaA07.WRI1* eGWAS at 20 DAF and 40 DAF. Gene names related to lipid metabolism are labeled on the plot. *FUS3*, *FUSCA3*; *CD1*, *CUTIN DEFICIENT 1*; *SHN3*, *SHINE3*; *MYB30*, *MYB DOMAIN PROTEIN 30*; *LEC1*, *LEAFY COTYLEDON 1*; *PDAT*, *PHOSPHOLIPID: DIACYLGLYCEROL ACYLTRANSFERASE*; *ROD1*, *REDUCED OLEATE DESATURATION 1*; *TED4*, *TRACHEARY ELEMENT DIFFERENTIATION-RELATED 4*; *TGD4*, *TRIGALACTOSYLDIACYLGLYCEROL 4*. **e** The distribution of overlapping ATAC peaks and eQTLs after randomly permuting the positions of ATAC peaks and eQTLs on the whole genome 1000 times at 20 DAF and 40 DAF. The inverted triangle represents the actual number of eQTLs in OCRs. The dashed lines indicate 95% confidence interval (CI) values

We mapped the genome sequences of 505 *B. napus* accessions re-sequenced in our previous study [50] to the ZS11 reference genome [55] and identified 11,700,689 genomic variants. In addition, a total of 583 transcriptomes from two seed development stages (309 accessions at 20 DAF and 274 accessions at 40 DAF) which were collected previously [50] were used for eQTL mapping. The transcriptome data were aligned to the ZS11 reference genome and identified 80,122 and 78,404 expressed genes at 20 DAF and 40 DAF, respectively. Based on genome-wide mapping, we identified 53,759 and 53,550 eQTLs for 79,605 and 76,713 genes (representing 99.35% and 97.84% of expressed genes) at two stages (Fig. 1a; Additional file 1: Fig. S2; Additional file 2: Tables S1 and S2). An average of six eQTLs were mapped per gene, with 53.7% and 44.1% of mapped genes having more than ten eQTLs affecting their expression at 20 DAF and 40 DAF, respectively (Fig. 1b; Additional file 2: Tables S3 and S4), possibly indicating a relatively complex regulatory mechanism for expression variation in *B. napus*. Based on whether an eQTL regulates gene expression nearby or distantly, all eQTLs were designated into local (most act as *cis*) eQTLs and distant (*trans*) eQTLs at 20 DAF and 40 DAF (local eQTLs_20 DAF_: 15,250, distant eQTLs_20 DAF_: 38,509, local eQTLs_40 DAF_: 14,679, distant eQTLs_40 DAF_: 38,871). Overall, 75.00% and 75.15% of the local eQTLs explained more than 20% of the expression variation at both stages of seed development, but the values were 45.53% and 50.04% in the distant eQTLs, respectively (Fig. 1c). We also noted that 32,002 eQTLs (73.72% and 75.67% distant eQTLs and 62.21% and 74.77% local eQTLs at 20 and 40 DAF, respectively) were detected at both stages (Fig. 1a), suggesting that the genetic effects on gene expression remain stable between two stages.

Although most of the eQTLs were consistently observed at both stages, we also found some stage-specific eQTLs. *WRI1* is a key regulator involved in the oil biosynthesis pathway and mainly plays a function in the initial stage of seed maturation [56]. *BnaA07.WRI1* had 11 eQTLs at 20 DAF and some known TFs regulating *WRI1*, such as *FUS3* and *LEC1*, were successfully identified. However, all these eQTLs were no longer detected at 40 DAF (Fig. 1d). In addition, we also found that some genes were only expressed at 40 DAF and eQTLs were detected at this time, such as *BnaA05.FAD7* (Additional file 1: Fig. S3), which is responsible for the synthesis of C16:3 and C18:3 fatty acids in galactolipids, sulfolipids, and phosphatidylglycerols [57, 58]. We further characterized the genes predicted to be regulated by eQTLs at a certain stage (eGene, defined as a gene in which the expression is significantly associated with an eQTL) and there were average of 8.64 eQTLs per eGene at 20 DAF and 8.19 eQTLs per eGene at 40 DAF. The GO enrichment analysis showed that eGenes at 20 DAF were involved in a variety of biological processes, such as substance synthesis, transport, and transcriptional activity. However, the eGenes at 40 DAF were significantly enriched in response to various stresses and metabolism processes (Additional file 2: Tables S5 and S6), indicating that these eGenes are under different genetic regulation at different developmental stages and are thus involved in the corresponding biological processes.

### OCRs are enriched for eQTL significant variants

Several lines of evidence suggest that genomic variations affect gene expression by perturbing *cis*-regulatory elements and altering the chromatin accessibility [37, 38]. To further investigate how genomic variations affect gene expression, we selected six representative accessions (ZS11, Westar, No2127, Zheyou7, Gangan, and Shengli) of *B. napus* for ATAC-seq using developing seeds at four stages (20, 26, 34, and 40 DAF) (Additional file 1: Fig. S4). A total of 59 ATAC-seq datasets were collected and 646,675 OCRs were identified across the genome (Additional file 2: Table S7). Correlation analysis showed clustering of samples was consistent with their origin, samples from the same accession clustered together (Additional file 1: Fig. S5). To assess whether eQTLs were enriched in OCRs or not, we investigated the significance of the overlap between OCRs and lead SNPs of eQTLs using a permutation-based approach. There were 9742 and 4626 lead SNPs located within the OCRs at 20 and 40 DAF, respectively, while the values of 95% confidence interval based on 1000 permutations were 7209 and 3442 (*P* < 0.01, Fig. 1e). For example, *BnaA08.TGD1*, which is involved in lipid transfer, had a stable local eQTL in the OCRs of ZS11 (Additional file 1: Fig. S6). Besides, an investigation of the contribution of eQTLs to explain phenotypic variation revealed that the eQTLs in OCRs had a greater impact (*P* = 1.16×10^−10^, Kolmogorov-Smirnov test) (Additional file 1: Fig. S7). These results are consistent with expectations under the assumption that OCRs harbor REs and the lead SNPs of eQTL are expected to be located in or near REs, and the combination of this resource allows further understanding of the regulatory mechanisms of eQTL.

### Local eQTL and chromatin states reveal expression piggybacking of adjacent genes

**Fig. 2:**
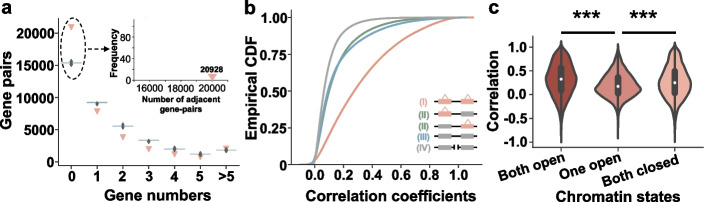
Local eQTL characterization. **a** Genes with local eQTLs occur more frequently at adjacent positions than expected by chance. The *x*-axis indicates the number of intervening genes between two genes in the ZS11 genome. The light red triangles indicate the number of gene pairs observed with local eQTL mapping. The blue histograms indicate the distribution of gene pairs from 1000 random permutations. The green histogram in the inset indicates the distribution of adjacent gene pairs based on 1000 random permutations. **b** CDF plots show that all adjacent gene pairs with any local eQTLs exhibit the highest expression correlation. Class I indicates the correlation coefficient distribution of adjacent genes with any local eQTLs. Class II indicates the correlation coefficient distribution of adjacent genes with local eQTLs detected on either gene. Class III indicates the correlation coefficient distribution of adjacent genes that do not map local eQTLs on both genes. Class IV indicates the correlation coefficient distribution of non-adjacent genes with local eQTLs mapped on both genes. **c** The violin plot depicts the highest correlation of expression of adjacent genes when local eQTLs regulating two class I adjacent genes which are in the same chromatin open state. In the horizontal axis, “Both open” indicates that the local eQTLs for both class I genes are located in the ATAC chromatin open region; “One open” indicates that the local eQTLs for only one class I gene are located in the ATAC chromatin open region. “Both closed” indicates that the local eQTLs of both class I genes are not located in the open region of ATAC chromatin. ***P < 0.001 compared with “One open” in Student’s *t* test

To investigate the characteristics of local regulatory variants in the *B. napus* genome, we extracted the location information of genes with any local eQTLs and analyzed their distribution in the genome. Among 38,614 genes with any local eQTLs, 20,928 adjacent gene pairs were formed, which was significantly greater than the number expected by random chance (*P* < 0.001, with 1000 permutations; Fig. 2a), indicating that local eQTLs appear to be in adjacent genes more frequently. We also found that the correlation of all adjacent genes’ expression was significantly higher than that of randomly sampled gene pairs (*P* < 0.001, Kolmogorov-Smirnov test; Additional file 1: Fig. S8). Furthermore, we investigated whether the expression correlation of adjacent gene pairs was influenced by local eQTL and divided the adjacent gene pairs into three classes: (I) two genes both have local eQTLs, (II) only one gene has local eQTL, and (III) two genes have no local eQTL. We also randomly selected gene pairs in the whole genome for correlation analysis. In adjacent gene pairs, the expression correlation was the highest when two genes have local eQTLs, and the lowest when two genes have no local eQTL (Fig. 2b). These results suggest that the expression changes of one gene are closely related to the expression of its adjacent gene in *B. napus*, and this expression piggybacking is stronger when two genes have local eQTLs in adjacent gene pairs. Interestingly, this phenomenon also occurred in maize [59].

To explore the effect of chromatin states of local eQTLs on expression piggybacking, we classified adjacent gene pairs both with local eQTLs into three types (eQTLs both in OCRs, only one in OCRs, and none in the OCRs) based on the chromatin opening states of the lead SNP for local eQTL. The analysis showed that gene expression correlations were significantly higher in the same chromatin state (i.e., eQTLs both in OCRs or none in the OCRs) than those with different chromatin states (*P* < 0.01; Fig. 2c). These results indicate that chromatin states influenced the local eQTLs, thus impacting the expression piggybacking effect of adjacent genes.

The above results indicate that expression piggybacking occurs widely in *B. napus* and is stronger when adjacent genes are both regulated by eQTLs in the same chromatin state. Therefore, comparing with the results in maize [59], we speculate that the phenomenon of expression piggybacking may be widespread in plants.

### Feedback regulation of homoeologous genes in *B. napus*

**Fig. 3:**
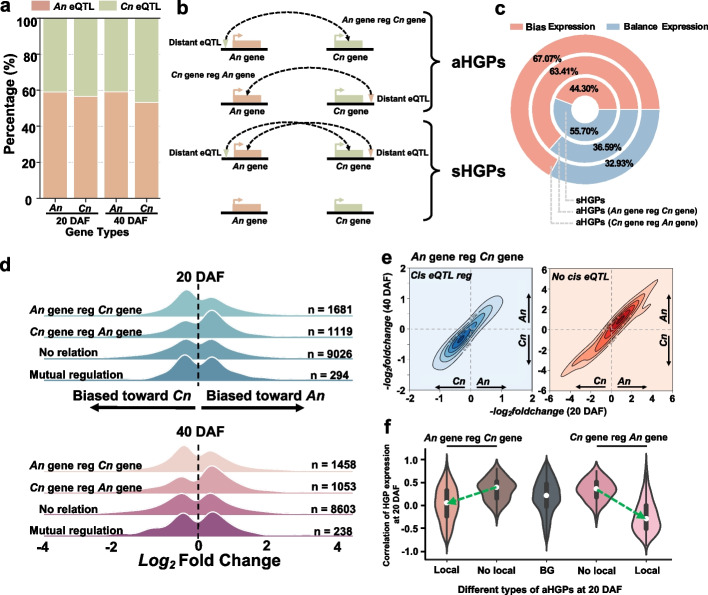
Homoeologous gene expression trend regulation. **a** The proportion of regulation between eQTLs and genes between *An* and *Cn* subgenomes at 20 and 40 DAF. The individual layer of the bars represents the percentage of data for that category to the overall data for that subgroup. The regulatory eGenes-eQTLs between *An* and *Cn* subgenomes were defined as four types of regulation. *An* eQTL regulates the *An* gene; *An* eQTL regulates *the Cn* gene; *Cn* eQTL regulates the *An* gene; *Cn* eQTL regulates the *Cn* gene. **b** The module of aHGPs and sHGPs. Each row represents a homoeologous gene pair (HGP), and the inverted triangle represents a distant eQTL. **c** The propensity of genome-wide homoeologous gene expression on different chromosomes of *An* and *Cn* subgenomes at 20 DAF. **d** The expression propensity distribution of aHGPs and sHGPs at 20DAF and 40DAF. The aHGPs contain “*An* gene reg *Cn* gene” and “*Cn* gene reg *An* gene,” and the sHGPs contain “No relation” and “Mutual regulation.” “n” in each row represents the number of each modulation combination. The *log*_*2*_*foldchange* represents the expression propensity. **e** Effect of local eQTLs on asymmetric regulation (*An* gene regulating *Cn* gene) of subgenomes. Horizontal coordinates indicate *log*_*2*_*foldchange* of HGPs at 20 DAF and vertical coordinates indicate *log*_*2*_*foldchange* of HGPs at 40 DAF. The left panel indicates affected by local eQTLs and the right panel represents unaffected by local eQTLs. **f** Comparison of gene correlations in different types of aHGPs at 20 DAF. “BG” represents all HGPs, “Local” represents aHGPs with local eQTLs, and “No Local” represents aHGPs without local eQTLs

The mapping of distant eQTLs provides *a wealth of information* for dissecting inter-subgenomic genetic regulation of polyploid plants and understanding the response of gene expression to allopolyploidy [10, 20]. We found that higher eQTL density on the *An* subgenome (55.83% and 56.15% of eQTL at 20 and 40 DAF, respectively) could be observed in the *B. napus* genome and that *An* gene expression was usually regulated by more eQTLs (average of 8.14 eQTLs per *An* genes and 7.77 eQTLs per *Cn* genes; Additional file 1: Fig. S9). Our analysis showed that nearly 60% of genes were regulated by the eQTL located in the *An* subgenome (Fig. 3a). These results indicate that regulation between subgenomes is unbalanced between *An* and *Cn* subgenomes, with the *An* subgenome having more abundant variations.

To explore the effect of imbalanced regulation between subgenomes on gene expression, we defined the mutually most similar gene pairs (sequence similarity reciprocates for best hit) between subgenomes as homoeologous gene pairs (HGPs) and a total of 27,689 HGPs were identified in *B. napu*s (Additional file 2: Table S8). We assumed that if a gene was in the distant eQTL of its homoeologous gene (150 kb before and after TSS), then it was likely to regulate the homoeologous gene. Based on this, when only one gene in the HGP was regulated by a homoeologous gene, the HGPs were considered as asymmetrically regulated homoeologous gene pairs (aHGPs). In addition to this, HGPs that were not mutually regulated, or regulated by each other, were defined as symmetrically regulated homoeologous gene pairs (sHGPs; Fig. 3b). Subsequently, an expression trend analysis of all HGPs suggested that HGPs had imbalanced expression between subgenomes with adjusted *P* value (padj) < 0.05 and demonstrated that aHGPs had a much higher percentage of gene imbalance expression than sHGPs (Fig. 3c; Additional file 1: Fig. S10; Additional file 2: Tables S9 and S10). This indicates that imbalanced regulation is more likely to result in imbalanced gene expression between *An* and *Cn* subgenomes.

Subsequently, we investigated the expression levels of HGPs in the subgenome at 20 and 40 DAF and found that in aHGPs with *An* eQTL regulating *Cn* gene, the *Cn* genes of aHGPs tend to be highly expressed and in aHGPs with *Cn* eQTL regulating *An* gene, the *An* genes of aHGPs tend to be highly expressed (Fig. 3d). These results show that aHGPs tend to be highly expressed in genes regulated by homoeologous genes. To explain this phenomenon, we analyzed HGPs with stable regulatory eQTLs at two stages. In aHGPs with *An* eQTL regulating *Cn* genes, aHGPs tend to be expressed in *Cn* genes when *An* genes were regulated by *cis*-variants. Conversely, aHGPs without local eQTLs tended to have a higher expression for *An* genes (Fig. 3e). The opposite phenomenon was also found for aHGPs with *Cn* eQTL regulating *An* genes (Additional file 1: Fig. S11). These results suggest that local eQTLs in aHGPs can influence the direction of expression propensity.

In addition, we performed gene expression correlation analysis between the *An* gene and *Cn* gene in aHGPs. We found that the correlation of aHGPs with local eQTLs had a significant decrease compared to aHGPs without local eQTLs, showing a negative correlation (Fig. 3f; Additional file 1: Fig. S12). These results suggest that aHGPs with local eQTLs have feedback regulation to maintain partial expression dosage between homoeologous genes. We also found aHGPs were mainly TFs (permutation-based test, *P* < 0.01; Additional file 1: Fig. S13). In summary, these results showed that the imbalanced regulation at the transcriptional level between subgenomes leads to unbalanced gene expression and the direction of expression propensity can be influenced by local eQTL. Moreover, it also suggested that there is a regulatory mechanism to maintain partial gene expression dosage among HGPs when the regulated genes in aHGPs have local eQTLs.

### Abundant distant eQTL hotspots exist in the genome

**Fig. 4:**
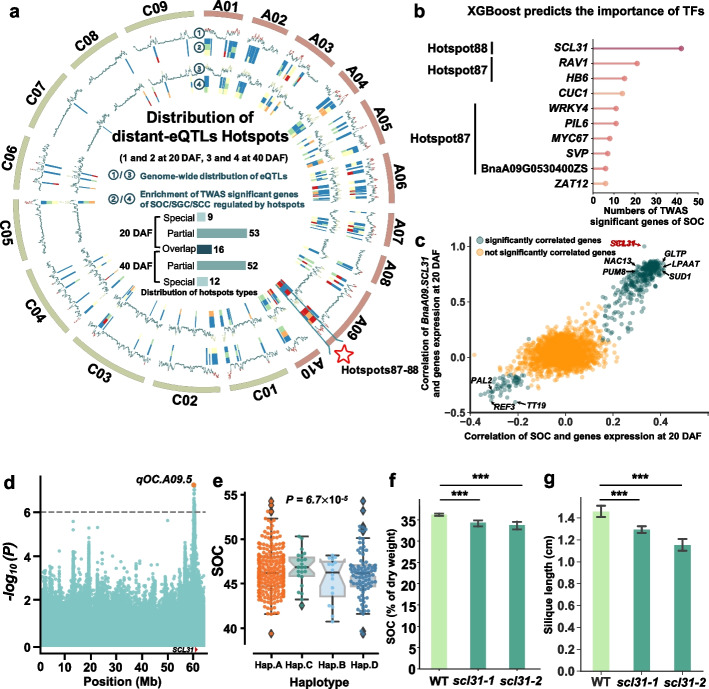
Distant eQTL hotspot identification. **a** The genomic distribution of distant eQTL hotspots and the enrichment of TWAS significant genes of SOC, SGC, and SCC in the hotspots. Dot plots represent the distribution of eQTL (outer: 20 DAF, inner: 40 DAF). And the red and light green dots indicate eQTLs in hotspots at 20 DAF and 40 DAF, respectively. Heatmaps represent the enrichment of TWAS significant genes of SOC, SGC, or SCC (outer: SOC, mid: SGC, inner: SCC) in hotspots at 20 DAF and 40 DAF (outer: 20 DAF, inner: 40 DAF). The pentagram indicates the location of Hotspot87-88. The bars indicate the number of hotspots. “Special” indicates the specific hotspot, “Partial” indicates the partially overlapping hotspot, and “Overlap” indicates the fully overlapping hotspot at two stages. **b** XGBoost predicts the importance of TFs. The horizontal coordinate indicates the statistics of the number of TFs ranked first in each TWAS significant gene of SOC prediction. The vertical coordinate indicates the ranking of the top 10 TFs predicted by XGBoost. **c** TWAS significant genes of SOC and reported SOC-related gene expression correlation analysis with *BnaA09.SCL31* and SOC. The horizontal coordinates indicate the correlation between gene expression and SOC at 20 DAF, and the vertical coordinates indicate the correlation between gene expression and *BnaA09.SCL31*. Green dots indicate significantly correlated genes and yellow dots indicate uncorrelated genes. **d** Manhattan plot on A09 chromosome. Points represent the log-transformed *P* values for variations from GWAS of SOC BLUP value. The bottom plot indicates the *BnaA09.SCL31* position. **e** Box plots for SOC, based on the haplotypes of variations in gene region and upstream 2-kb region of *BnaA09.SCL31*. **f** SOC phenotype of *SCL31* T-DNA mutant lines (*scl31-1*, *scl31-2*) in *Arabidopsis thaliana*. Values are means (SD; *n* = 5 biological repeats). ***P < 0.001 compared with WT in Student’s *t* test. **g** Silique length phenotype of *SCL31* T-DNA mutant lines (*scl31-1*, *scl31-2*) in *Arabidopsis thaliana.* Values are means (SD; *n* = 5 biological repeats). ***P < 0.001 compared with WT in Student’s *t* test

When observing the genomic distribution of distant eQTLs, we found that a large number of eQTLs clustered in certain regions, forming eQTL hotspots (Additional file 1: Figs. S9 and S14). Finally, we identified a total of 141 significant eQTL hotspots at 20 and 40 DAF (Additional file 2: Tables S11 and S12). Interestingly, most of these regulatory hotspots were distributed on the *An* subgenome (*An*:*Cn* = 97:45). Comparison of the eQTL hotspots at two stages showed only 21 hotspots were completely specific (9 hotspots at 20 DAF, 12 hotspots at 40 DAF), which accounted for 14.89% of the total number of eQTL hotspots (Fig. 4a). These results indicate that the distant eQTL distribution is relatively stable during seed development of *B. napus*.

To further investigate whether these eQTL hotspots were involved in the regulation of seed traits, we performed the enrichment analysis between genes regulated by eQTL hotspots and trait-associated genes identified by TWAS. The published phenotype data of SOC, seed glucosinolate content (SGC), and seed coat content (SCC) [50, 51, 60] were used to perform TWAS (Additional file 2: Tables S13 and S14). Subsequently, we found that the SOC-, SGC-, and SCC-related hotspots were mainly located in the *An* subgenome (Fig. 4a). SOC-related aHGPs with local eQTLs were able to maintain a partial homoeolog expression dosage, such as *ADD5* and *PDAT1* (Pearson’s correlation coefficient [PCC] *R*_*ADD5*_ = −0.75, PCC *R*_*PDAT1*_ = −0.35). However, the HGP of *WRI1* without local eQTL does not maintain homoeolog expression dosage, showing that expression is highly correlated (PCC *R*_*WRI1*_ = 0.84; Additional file 1: Fig. S15). Interestingly, there were two adjacent hotspots (hotspot 87, hotspot 88) on chromosome A09 that could regulate 69.73% TWAS significant genes of SOC (Additional file 1: Fig. S16). We combined these two hotspots and named it Hotspot87-88. These results suggest that Hotspot87-88 on A09 plays an important role in regulating SOC (Additional file 2: Tables S15 and S16).

### Combining distant eQTL hotspots and XGBoost module to identify key TFs affecting SOC

Although we have identified important regulatory hotspots that regulate SOC, the identification of regulatory genes in hotspot regions remains challenging. It is known that TFs play an important role in gene regulatory networks. There are often some key TFs in eQTL regulatory hotspots that influence the phenotype by regulating the expression of a series of downstream genes [5, 30]. To find the key TFs affecting SOC regulatory genes in Hotspot87-88, we constructed an XGBoost model to prioritize possible upstream TFs based on a gradient boosting decision tree (Additional file 1: Fig. S17). Based on this, we performed upstream TF prediction for TWAS significant genes of SOC whose eQTLs were located in Hotspot87-88. For each TWAS significant gene of SOC, the top 3 TFs ranked were extracted as possible upstream TFs by the XGBoost model. Afterwards, we ranked all genes predicted to be upstream TFs according to the number of occurrences, and the TFs that were predicted to regulate more TWAS significant genes of SOC were ranked higher. Thus, we predicted *BnaA09.SCL31* (BnaA09G0670500ZS) to be the key TF regulating TWAS significant genes of SOC in Hotspot87-88 (Fig. 4b, c). *BnaA09.SCL31* located in Hotspot87-88 and its different genotypes of eQTL lead SNP significantly associated with its own gene expression, TWAS-SOC significant gene expression, and SOC (Additional file 1: Fig. S18).

Interestingly, GWAS for SOC showed that *BnaA09.SCL31* was also located within the SOC QTL *qOC.A09.5* in our previous study [50]. By distinguishing the coding region and the upstream 2-kb promoter of *SCL31* into four haplotypes, significant differences were observed between SOC and expression value corresponding to various haplotypes (*P*_SOC_ = 6.7×10^−5^; *P*_expression_ = 5.19×10^−3^; Fig. 4d, e; Additional file 1: Fig. S19a). We further found that gene expression of *BnaA09.SCL31* was significantly positively correlated with SOC (PCC *R*_20 DAF-SOC_ = 0.33, *P*_20 DAF-SOC_ = 1.78×10^−8^; Additional file 1: Fig. S19b).

To verify the function of *SCL31*, we applied two *Arabidopsis* T-DNA insertion mutants of *SCL31* (one inserted in the promoter and the other in the exon) to examine the SOC (Additional file 1: Fig. S20). Compared with WT, the mutants had a relatively stable fatty acid composition and showed a significant decrease in SOC. Besides, we also observed shorter siliques and smaller seeds in the mutants (Fig. 4f, g; Additional file 1: Figs. S21 and S22). These results suggest that *SCL31* is an important TF positively affecting SOC, silique length, and seed sizes.

### Predicting regulatory mechanisms affecting SOC

**Fig. 5:**
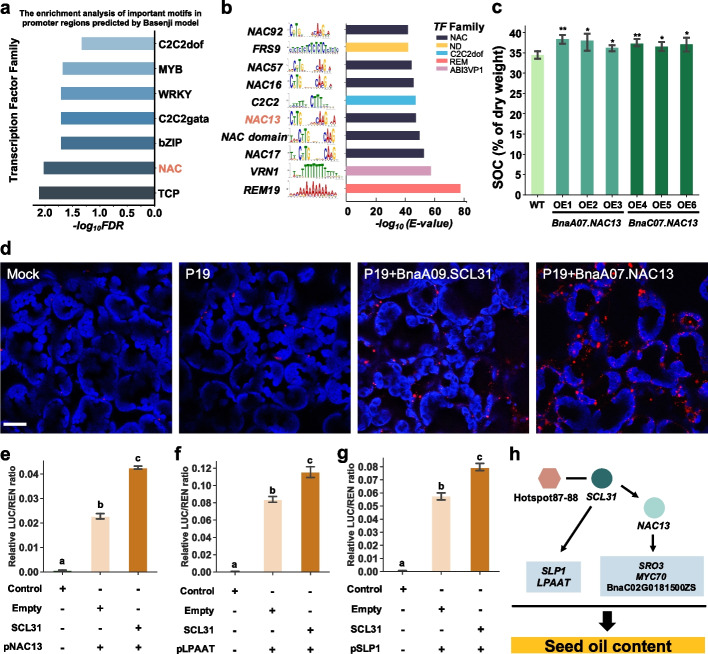
Predict the regulatory relationships affecting SOC TFs. **a** Enrichment of the Basenji model predicted key TF motifs in promoter sequences of TWAS significant genes of SOC. *-log10 (FDR)* represents the significant enrichment level. **b** Bar graph demonstrates the enrichment of the *NAC* motif in the promoter sequences of TWAS significant genes of SOC regulated by Hotspot87-88. *-log10 (E-value)* represents the significant enrichment level. **c** SOC phenotype of OE lines (*BnaA07.NAC13*, *BnaC07.NAC13*). Values are means (SD; *n* = 5 biological repeats). *P < 0.05 compared with WT and **P < 0.01 compared with WT in Student’s *t* test. **d** Representative confocal images of LDs (BODIPY 493/503 fluorescence, false color red) in tobacco leaves. The blue color shows chloroplast auto-fluorescence. Images were collected at the same magnification. Mock infection was infiltrated with media only; P19 was used as a viral suppressor of transgene silencing and was coexpressed in all BnaA09.SCL31 and BnaA07.NAC13 treatments. Bar = 20 μm. **e**–**g** Bar graph showing the relative LUC/REN ratio in the dual-luciferase assay. Values are means (SD; *n* = 3 biological repeats). *BnaC03.SLP1*(*SHEWENELLA-LIKE PROTEIN PHOSPHATASE 1.***h** A model map predicting the regulatory mechanism of Hotspot87-88 in regulating SOC. The hexagonal shape shows the hotspot region, the circular shape represents the TFs, and the blue box inside represents the downstream TWAS significant genes of SOC

In order to further explore the regulatory mechanisms of TWAS significant genes of SOC, we trained the Basenji, which uses convolutional neural networks to predict chromatin accessibility (Additional file 1: Fig. S23). The trained Basenji model showed high consistency between the predicted and observed features (Additional file 1: Fig. S24). Basenji can also predict the effect of genomic variants on chromatin accessibility and TF binding. Based on this model, we detected key motifs in the 2-kb promoter of TWAS significant genes of SOC and then annotated the key motif by comparing for similarity with known motifs by Tomtom [61] (Fig. 5a; Additional file 2: Table S17). Find Individual Motif Occurrences (Fimo) can be applied to scan DNA sequences with motifs described as position-specific scoring matrices [62]. We also used Fimo to scan the 2-kb promoter of TWAS significant genes of SOC (Fig. 5b). These results showed that the NAC family motifs were significantly enriched in the Basenji and Fimo results. And *NAC13* had a significantly enriched motif among the NAC family (Fig. 5a, b). Subsequently, we integrated the eQTL results of TWAS significant genes of SOC and selected six genes (*BnaC06.GLTP*, *BnaA06.RIN4*, *BnaA04.MYC70*, *BnaC04.MYC70*, *BnaC04.PBL30*) whose eQTL interval harbor *NAC13* and had NAC motif in its promoter, and we also found that the expression of these genes was highly correlated with *BnaA07.NAC13* and *BnaC07.NAC13* (PCC *R* from 0.55 to 0.8; Additional file 1: Fig. S25). To further confirm the prediction, we performed a transient dual-luciferase assay to verify the results and found that *BnaC04.MYC70* and BnaC02G0181500ZS were activated by *BnaA07.NAC13* (Additional file 1: Fig. S26). These results suggest that *NAC13* may affect SOC by regulating TWAS significant genes of SOC.

*NAC13* has not been reported to impact SOC in oilseed plants. To investigate whether *NAC13* affects the SOC, we performed heterologous overexpression of *BnaA07.NAC13* and *BnaC07.NAC13* in *Arabidopsis* using the CaMV35S promoter. The quantitative RT-PCR showed a significant increase in *BnaA07.NAC13* and *BnaC07.NAC13* in the pure overexpression lines (Additional file 1: Fig. S27). We harvested the mature seeds of T2 overexpression lines and the SOC results showed that overexpression of *BnaA07.NAC13* and *BnaC07.NAC13* in *Arabidopsis* significantly increased (Fig. 5c), indicating that *NAC13* positively regulated SOC. Compared with WT, the fatty acid composition was also altered (Additional file 1: Fig. S28). Surprisingly, we found that the eQTLs of *BnaA07.NAC13* were also localized in Hotspot87-88 at 20 DAF and 40 DAF (Additional file 1: Fig. S29). Furthermore, *BnaA07.NAC13* also showed the most significant positive gene expression correlation with *BnaA09.SCL31* (PCC *R*_20 DAF_ = 0.82, *P*_20 DAF_ = 8.86×10^−68^) (Additional file 1: Figs. S30 and S31).

To provide solid evidence, we transiently expressed BnaA09.SCL31 and BnaA07.NAC13 in tobacco leaves. Examination by confocal microscopy revealed that BnaA09.SCL31 and BnaA07.NAC13 increased the numbers of lipid droplets (LDs) but did not significantly change the size of LDs in leaf cells (Fig. 5d; Additional file 1: Fig. S32). This indicates that *BnaA07.NAC13* and *BnaA09.SCL31* have a direct effect on lipid accumulation. And then we examined the relative expression of *NAC13* by quantitative RT-PCR in *SCL31* mutants. The results showed that the relative expression was significantly decreased. In addition, the relative expression of some genes related to lipid metabolism (*SUPPRESSOR OF DRY2 DEFECTS 1* [*SUD1*], *LPAAT*) and some other TWAS significant genes of SOC (*SCL30*, *SHEWENELLA-LIKE PROTEIN PHOSPHATASE 1* [*SLP1*]) were also significantly decreased compared to WT (Additional file 1: Fig. S33). And then, we measured the effect of *BnaA09.SCL31* on *BnaA07.NAC13* promoter activity using transient dual-luciferase reporter assays. There was a significant increase in relative LUC/REN values with the addition of *BnaA09.SCL31* plasmid vector compared to the empty (*P* = 9.88×10^−8^; Fig. 5e; Additional file 1: Fig. S34a). Furthermore, for some TWAS significant genes of SOC that were highly positively correlated with *BnaA09.SCL31*, such as *BnaC07.LPAAT* and *BnaC03.SLP1*, adding *BnaA09.SCL31* plasmid vector also led to a significant increase in the relative LUC/REN values (*P*_*LPAAT*_ = 1.80×10^−4^, *P*_*SLP1*_ = 5.13×10^−6^) (Fig. 5f, g). These suggest that *BnaA09.SCL31* can affect the expression of *BnaA07.NAC13*, *BnaC07.LPAAT*, and *BnaC03.SLP1*. Moreover, for the *BnaA07.SRO3* (*SIMILAR TO RCD ONE 3*), a TWAS significant gene of SOC with a key NAC motif in the promoter region predicted by the Basenji model, the experimental results showed that *BnaA07.NAC13* was also able to affect the expression of *BnaA07.SRO3* (*P* = 3.35×10^−3^; Additional file 1: Fig. S34b). Therefore, based on these results, we speculate that *SCL31* may influence TWAS significant genes of SOC expression by regulating *NAC13* and multiple downstream genes to impact SOC (Fig. 5h).

### A comprehensive web-based tool for exploring eQTL variation in *B. napus*

**Fig. 6:**
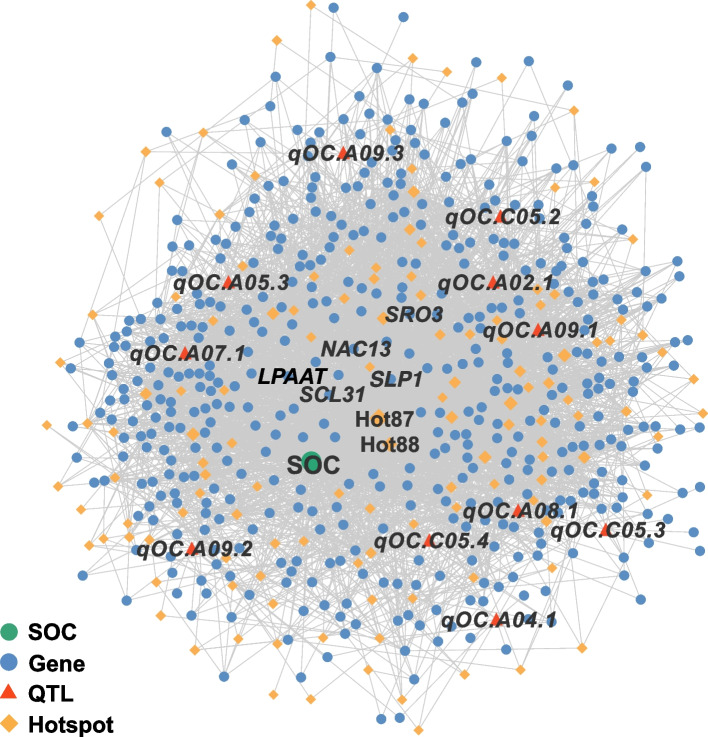
SOC-GWAS-TWAS-eGWAS regulated network. Green circles represent SOC, and blue circles represent 422 TWAS significant genes of SOC. Triangles represent 11 GWAS significantly associated QTLs of SOC. Diamonds represent 141 eQTL hotspots, and the shape size represents the number of regulatory genes. The two eQTL hotspots with the most regulated genes are Hotspot 87 and Hotspot 88

To better understand the regulatory mechanism of oil biosynthesis and seed development of *B. napus*, we integrated the results of eGWAS, GWAS, and TWAS to construct a comprehensive multi-omics eQTL regulatory network. The network contained 11 GWAS significantly associated QTLs of SOC, 424 TWAS significant genes of SOC, and 141 eQTL hotspots, indicating that oil biosynthesis is extremely complex in *B. napus* (Fig. 6). Lastly, we constructed a comprehensive web-based tool Bn-eQTL database with useful tools for searching eQTLs by genes or SNPs and querying gene expression information (http://yanglab.hzau.edu.cn/BneQTL/).

## Discussion

### Asymmetric distribution of eQTLs between *B. napus* subgenomes

*B. napus* originated about 7500–10,000 years ago [52] and was domesticated only 400–500 years [63]. *B. napus* is an allotetraploid crop formed by the natural cross and doubling of *Brassica rapa* (AA, 2n = 20) and *Brassica oleracea* (CC, 2n = 18) [52, 53]. A large number of these non-homoeologous chromosomal recombination events resulted in a high abundance of genetic variation within *B. napus* [64, 65]. Studying the imbalance characteristics of polyploid genomes can be a key tool to resolve the regulatory mechanisms of polyploid crop evolution, domestication, and improvement [15, 16, 66, 67]. In the past studies, previous authors applied epigenome mapping [11] and GWAS [53] of *B. napus* to reveal imbalanced transcription and different evolutionary trends among subgenomes, indicating that the overall gene expression and activity epigenetic signal modification levels were significantly stronger on the *An* subgenome than the *Cn* subgenome, and the ratio of expression bias and epigenetic activity bias of homologous gene pairs on *An* and *Cn* is symmetric [11, 68]. In this study, we used eQTL analysis to find that *An* had higher genetic variation than the *Cn* subgenome and *An* also had more hotspot regulatory regions at both seed development stages. Hotspot87-88 on chromosome A09 were found to affect SOC mainly through regulating TWAS significant genes of SOC (Fig. 4a). Besides, the analysis of homoeologous gene pairs between subgenomes revealed that asymmetric transcriptional regulation causes expression asymmetry, while local eQTLs affected the gene expression trend between subgenomes (Fig. 3e; Additional file 1: Fig. S11). These results provide new insights for broadening the genetic basis and selective breeding in *B. napus*.

### TWAS combined with machine learning to resolve key genes regulating hotspot regions

Distant eQTL hotspots can lead to expression changes of multiple downstream genes. In previous eQTL studies in crops, the identification of hotspot regulatory regions was important to reveal the regulatory relationships between genes and traits as well as to construct a gene regulatory network [5, 7, 19, 69]. However, mining key genes in the regulatory regions of hotspots and inferring gene regulatory networks are still challenging.

In our study, we used eQTL analysis to identify important hotspots on chromosome A09 that regulate TWAS significant genes of SOC (Fig. 4a). In addition, we constructed a machine learning method based on the Boosting integration model (XGBoost) using the expression of regulated genes and TFs to build a feature selection integration framework for gene expression data [27]. This method has been not used in the previous eQTL studies. In addition, we calculated the initial weights of gene regulatory relationships based on the established feature selection integration framework (Additional file 1: Fig. S17). Based on this, our results indicate the two TFs *NAC13* and *SCL31* could regulate TWAS significant genes of SOC (Fig. 5h). In the *SCL31* mutants, in addition to a significant decrease in SOC, shorter siliques and smaller seeds were also observed. We also found *BnaA09.SCL31* haplotypes were significantly associated with thousand seed weight (TSW; *P* = 3.0×10^−6^), and the expression of *BnaA09.SCL31* in 40 DAF was positively correlated with TSW (PCC *R*_40 DAF-TSW_ = 0.18, *P*_40 DAF-TSW_ = 4.04×10^−3^). These results suggest that *SCL31* may also play a pleiotropic role in plant growth and development besides regulating SOC (Fig. 4f, g; Additional file 1: Fig. S22). However, we only applied the population transcriptome data at 20 DAF and 40 DAF and there may be temporal limitations during seed development. We believe that it is possible to resolve the dynamic regulatory processes of genes and TFs if they can be analyzed in conjunction with multiple key time points during seed development.

### ATAC-seq combined with deep learning to resolve causal variants

GWAS has been proposed and widely practiced to resolve the genetic basis of crop traits, but it has been particularly difficult to resolve the underlying mechanisms of variation in non-coding regions [22]. Variants in non-coding regulatory regions are also an important factor in phenotypic variation and their alteration of gene expression levels and patterns has a broader scope for genetic improvement. In previous studies, Zachary Lippman et al. obtained several yield-enhancing regulatory region variants by editing the tomato *CLV3* promoter region [70] and Zeng et al. improved the quality of rice by editing the promoter and 5′ UTR-intron region of the *Waxy* gene [71]. Recent studies have improved maize yield by editing the promoter of the maize *CLE* gene, overcoming the disadvantage of known related mutants that had abnormal development [72]. However, how to pinpoint the linkage between core regulatory elements and phenotypes on a genome-wide scale and further explore the regulatory pathways of genes remains a pressing challenge.

With the development of high-throughput sequencing technology, a large amount of high-throughput ATAC-seq data has been generated [73–76]. Our study applied ATAC-seq to predict the binding sites of TFs and OCRs on a genome-wide scale (Additional file 1: Fig. S4). Besides, Basenji (one deep convolutional neural network module) predicted the variation of human genomic loci affecting gene expression with consistent information with eQTL results, showing the excellent prediction ability [77]. In future studies, we can also combine Basenji with ATAC-seq to construct convolutional deep learning network models to predict the key regulatory elements in gene promoter regions, providing the possibility to resolve the epigenetic state and regulatory processes of *B. napus* genes.

## Conclusions

Taken together, in this study, we used large-scale transcriptome data of oilseed rape populations, ATAC-seq data combined with eGWAS, GWAS, machine learning models, and deep learning models to identify key transcription factors and regulatory networks regulating SOC in oilseed rape. This study will greatly enrich our understanding of the regulatory mechanisms of oil metabolism.

## Methods

### Data sources

In this study, we applied resequencing data from 505 *B. napus* accessions. From these, developing seeds of 309 at 20 DAF and 274 at 40 DAF *B. napus* accessions were selected for whole transcriptome sequencing [50]. Besides, we used 273 time-series and multi-tissue transcriptome data covering the entire growth period of rapeseed cultivar ZS11 to construct the regulatory network [78]. We also obtained the 59 ATAC-seq samples from six accessions of *B. napus* (ZS11, Westar, No2127, Zheyou7, Gangan, and Shengli) at four stages of 20, 26, 34, and 40 DAF which were newly generated data in this research.

### Variation calling, genotype filling, and annotation

The rapeseed genome (ZS11 v0) was downloaded from BnPIR [79] (http://cbi.hzau.edu.cn/bnapus/index.php). The reads were aligned to the reference genome with BWA software [80] with the command “mem -M -k 32 -t 4.” The PCR duplicates of sequencing reads were removed with SAMTools [81], and the mapping reads were retained in BAM format. Then, the Genome Analysis Toolkit (GATK v3.6) [82] was used to identify the sequencing variations among 505 accessions with the HaplotypeCaller module and command “-T HaplotypeCaller -allowPotentiallyMisencodedQuals –emitRefConfidence GVCF.” GVCF files were merged with the “GenotypeGVCFs” command. SNPs and InDels were filtered if mapping quality < 20 or sequencing depth < 50 in the whole population. After obtaining the genotype, the LD-KNN algorithm was used to estimate the missing genotype [83].

### Linkage disequilibrium

For the linkage disequilibrium analysis, the decay of LD uses PLINK v1.9 [84] software and is calculated based on the value of *r*^2^ and the corresponding distance between the two SNPs. We set the gene composition to a 4-Mb region and set a 2-Mb overlap between the windows. The detailed parameters of each window are as follows: “--r2 --ld-window-r2 0 --allow-extra-chr- ld-window 99999 --ld-window-kb 99999 --allow-no-sex.”

### Transcriptome-wide association study for SOC and SGC

TWAS methods were designed according to our previously established study [50], but we used the ZS11 v0 rapeseed genome for TWAS in this study. Some low-expressed genes (the 95th percentile of log2-transformed expression values) in the population were excluded from the analysis, and finally, 80,122 and 78,405 expressed genes were obtained at 20 DAF and 40 DAF, respectively. Association analysis was performed using EMMAX software [85] and FDR-corrected *P* value ≤0.05 was used as the significance threshold for TWAS.

To assess the accuracy of transcript abundance estimation by mapping RNA-seq reads to the *B. napus* genome, we simulated RNA-seq datasets using gene models only from one of the ZS11 *An* subgenome and then used all *An* gene from the ZS11 reference genome to calculate TPM. Simulation performed for the *An* showed a high level of correlation (PCC *R* = 0.91, *N* = 41338) between the real values and those estimated. This indicates a high accuracy of transcript abundance estimation for homologous gene sets between subgenomes.

### The identification of eQTL and distant eQTL hotspots

The linear model in the FaST-LMM v0.3.8 [86] package was used to combine 11,700,689 high-quality SNPs (MAF > 0.05) corresponding to 20 DAF and 40 DAF for eGWAS. The explained variance (*r*^2^) of LeadSNPs was calculated by FaST-LMM v0.3.8. The significance threshold of the association was calculated by the software GEC [87] as 1×10^−6^ and a total of 377,616 and 373,073 significantly associated SNPs were detected at 20 DAF and 40 DAF, respectively. Combining SNPs was performed according to the criterion of LD > 0.2. Based on the position of lead SNP in relation to the target genes, if the SNP is located within 500 kb (the distance of LD decay [*r*^*2*^] to 0.1 is about 500 kb) upstream and downstream of the gene, it was considered a local eQTLs, otherwise a distant eQTL.

To identify the potential distant eQTL hotspot regions, distant eQTLs were randomly assigned in the genome, the number of eQTLs randomly distributed throughout the genome was scanned using 1-Mb size intervals and 100-kb step size, and subsequently, the maximum number of eQTLs within the 1-Mb interval was retained. After 1000 random permutations, we defined a genome-wide QTL number greater than 81 (*P* < 0.01) within 1Mb as a hotspot regulatory region based on the distribution of the maximum number of eQTLs in the permutation (Additional file 1: Fig. S14). Any overlapped or adjacent hotspots that may correspond to a single hotspot were combined into one, resulting in 141 potential hotspots at 20 DAF and 40 DAF. The distant eQTL hotspots were visualized using Circos v.0.69-6 [88].

### ATAC-seq data analysis

ATAC-seq experiments were designed according to the previously established experimental procedure [89]. We applied the constructed ATAC-seq library and completed sequencing on the Illumina Hiseq at the Huazhong Agricultural University sequencing platform and obtained raw reads. ATAC-seq reads were aligned to the ZS11 v0 genome [79] by BWA [80] using default parameters. The high mapping quality reads (MAPQ > 30, qualified reads) were used for further analysis. We divided the genome with a bin of 100 bp and extended 75 bp at both ends of each bin. We extracted the number of ATAC-seq fragment ends and quantified and normalized them. Multidimensional scaling was performed on these fragment counts by the python sklearn package [90] (https://scikitlearn.org). Peaks were identified using MACS2 v2.2.7.1 [91] with the parameters “-g 1.13e9 --nomodel --extsize 38 --shift -15 --keep-dup all -B --SPMR --call-summits.” The default parameters of BEDTools [92] merge the peaks for each sample.

### XGBoost model construction

We applied 273 ZS11 RNA-seq data [78] and 583 *B. napus* accession RNA-seq data [50] to construct the XGBoost model. The TFs in *Arabidopsis* were downloaded from PlantTFDB (http://planttfdb.gao-lab.org/), and homoeologous TFs of *Arabidopsis* and *B. napus* were compared using blast (https://blast.ncbi.nlm.nih.gov/). A total of 6587 *B. napus* TFs were obtained. GRN was built using the Python machine learning libraries scikit-learn v.0.22.2.post1 and XGBoost v.1.4.0 [27, 90]. The transformed TPM matrix and the list of putative TFs were used to train the XGBboost model for each dataset using the XGBRegressor with the parameters “-n_estimators 1000 -max_depth 3-learning_rate 0.0001 -reg_alpha 0 -reg_lambda 1” [27, 90].

### Construction of the deep learning model

Fifty-nine *B. napus* ATAC-seq samples were used as input data for Basenji model training, and all duplicate sequences with failed original genome assembly were removed in the sampling process. The Basenji model took a long fragment of 131,072-bp DNA sequence as input and counts the sum of the number of reads obtained from ATAC-seq sequencing in this region in units of 128 bp and then wrote it to an HDF5 file together with the sampled sequences of single heat encoding to complete the partitioning of the dataset during the sequence sampling process [93]. After the HDF5 file was obtained, the data were input into the Basenji model for deep learn training [77].

### Plant growth conditions and T-DNA insertion mutant identification

*Arabidopsis* ecotype Columbia-0 was used as the WT control. We ordered *scl31-1* (SALK_043461) and *scl31-2* (SALK_105042) *Arabidopsis* mutants from the company (https://m.arashare.cn/pages/batch/batch). The homozygous T-DNA insertional mutants were verified by PCR-based screening using a T-DNA left border primer and gene-specific primers. Primers used for the PCR verification of *scl31-1* and *scl31-2* were listed (Additional file 2: Table S18). We also constructed an overexpression vector (p35S-FAST-*BnaA07.NAC13* and p35S-FAST-*BnaC07.NAC13*) that was used for genetic transformation to obtain *Arabidopsis* overexpression lines. *Arabidopsis* genetic transformation mediated by *Agrobacterium tumefaciens* was performed by the flower dipping method [94]; T0 generation seeds were harvested and cultured in a 16-h light/8-h dark light chamber to harvest T1 generation seeds, after which the transgenic seeds were harvested from T1 generation plants by means of an incubator containing a kanamycin concentration of 50 ng/μL of 1/2 MS culture medium for screening, followed by transplantation of the screened *Arabidopsis* plants into the growth chamber and further identified T2 generation *Arabidopsis*-positive seedlings and homozygous lines for phenotypic investigations. All plants were grown in a growth room with a 16-h light/8-h dark cycle at 23/21°C, 50% humidity, and 100 μmol m^−2^ s^−1^ of light intensity.

### RNA isolation and qRT-PCR

For quantitative RT-PCR (qRT-PCR) analysis, total RNA was extracted from per sample (∼60–80 mg tissue) using TransZol Plant Reagent (TransGen, China). Then, 2 μg was used to synthesize cDNA with EasyScript® First-Strand cDNA Synthesis SuperMix Kit according to the manufacturer’s instructions (TransGen, China). Primers were designed using NCBI primer design software and are listed in Table S18. To ensure the reliability and validity of the qRT-PCR experiments, one reference gene, *AtACTIN7*, was used. PCR reactions were set up in 96-well Hard-Shell PCR plates (Bio-Rad, USA) with 0.4-μM primers using SYBR Premix TransStart® Green qPCR SuperMix (TransGen, China) in 15 μL and run on a Bio-Rad CFX Connect. The data were collected from three biological replicates and three technical replicates and expressed as the mean ± standard error (mean ± SE). PCR conditions were as follows: one cycle of 95 °C for 1 min; 45 cycles of DNA melting at 95 °C for 10 s, DNA annealing at 60 °C for 10 s, and DNA extension at 72 °C for 30 s; and a final extension of DNA at 72 °C for 10 min.

### Determination of seed fatty acid composition and SOC

We used a gas chromatography-flame ionization detector (GC-FID) to analyze the quality of rapeseeds harvested at maturity and obtained the data of seed fatty acid composition and SOC [95]. Briefly, we weighted 4–6 mg of *Arabidopsis* seeds and add 4 mL of extraction solution (5% H_2_SO_4_, 95% methanol, 0.01% BHT) and 100 μL of internal standard 16.2 μM/mL heptadecanoic acid (C17:0) at 85°C for 2 h to methyl esterify the fatty acids. After it cooled to room temperature, 3.0 mL of hexane and 3.0 mL of H_2_O were added, vortexed and mixed, and centrifuged at 1000 r/min for 10 min, and 1.0 mL of supernatant was taken into the injection vial. The gas chromatography was equipped with a hydrogen flame ionization detector and a capillary RESTEK Rtx-wax column (0.25 mm×30 m) with a helium carrier of 20 mL/min. The oven temperature was kept at 170°C for 1 min and then gradually increased to 210°C at a rate of 3°C/min. GC-MS was automatically injected for analysis. Finally, the fatty acid species were identified according to the retention time. The peak area data corresponding to each fatty acid component were compiled. Using the internal standard heptadecanoic acid (17:0) as a reference, the content of each fatty acid was used for the calculation of SOC as a percentage of the dry weight and fatty acid composition was calculated as mol%.

### Dual-luciferase assay

Dual-luciferase assays were performed by using the Dual-Luciferase^Ⓡ^Reporter Assay System (Promega, Madison, WI, USA). All reagents were prepared according to the manufacturer’s description. The *BnaA07.NAC13*, *BnaC07.LPAAT*, *BnaC03.SLP1*, *BnaA07.SRO3*, *BnaA04.MYC70*, and BnaC02G0181500ZS promoters were amplified and inserted into the pGreenII0800-LUC vector as the reporter plasmid (Additional file 2: Table S18). The ORF of *BnaA09.SCL31* and *BnaA07.NAC13* was amplified and inserted into the pGreenII-SK vector as the effecter plasmids. The method was performed in *Arabidopsis* protoplasts according to Yoo et al. [96]. After 16 h, the transformed *Arabidopsis* protoplasts were dissolved into 100 μL of passive lysate. After 30s, a 50-μL aliquot was used for luminescence measurements with the SPARKR^Ⓡ^MULTIMODE MICROPLATE (TECAN, Swiss). The following steps were used for luminescence measurements: 50 μL of the firefly luciferase reagent (LARII) was added to the test sample, with a 10-s equilibration time and measurement of luminescence with a 10-s integration time, followed by addition of 50 μL of the REN reagent and firefly quenching (Stop and Glow TM buffer), 10-s equilibration time, and measurement of luminescence with a 10-s integration time. The data were represented as the ratio of firefly to *Renilla* luciferase activity (Fluc/Rluc). Each data point consisted of at least three biological replicates and 15 repeats were performed for each assay.

### Tobacco transient transformation and lipid droplet labeling analysis

The recombinant vectors pBin-BnaA09SCL31-GFP and 1305-35S-Bna.NAC13-GFP were transformed into *Agrobacterium tumefaciens* (GV3101) and a mixture of *Agrobacterium tumefaciens* containing the vectors was prepared according to the previous method [97, 98]. Three-week-old tobacco was taken and injected into the leaf pulp using a syringe with the needle removed and placed firmly against the back of the tobacco leaf. After dark incubation overnight and light incubation for 2 days, the infested leaves were cut into 1–2-mm pieces and placed in 2-mL centrifuge tubes and stained with BODIPY staining solution (2μg/mL in 50-mM pipes buffer) for 45 min under vacuum, followed by 15 min of washing with 50-mM pipes under vacuum and repeated three times. After resting on ice, the protein expression localization and lipid droplet staining were observed under a laser confocal microscope. The lipid droplets emitted bright red fluorescence, and the number of lipid droplets was quantified using ImageJ software.

### Construction of the SOC-GWAS-TWAS-eGWAS regulated network and Bn-eQTL database

The eGWAS analysis was performed on each of the 422 TWAS significant genes of SOC using information from 27 QTLs identified in the previous study. A QTL significantly associated with the expression of a gene was an eQTL for that gene. Based on the hotspot and QTL information, a gene network regulating SOC was constructed. And then, we constructed the Bn-eQTL database (http://yanglab.hzau.edu.cn/BneQTL/) based on the Python Django framework (https://djangoproject.com).

## Supplementary Information

Additional file 1: Fig. S1. Overview of experimental and research analysis methods. Fig. S2. Venn diagram of the distribution of genes regulated by different types of eQTLs (local eQTL and distant eQTL) at 20 DAF and 40 DAF. (a) Distribution number of genes which were regulated by different types of eQTLs at 20 DAF. (b) Distribution number of genes which were regulated by different types of eQTLs at 40 DAF. Fig. S3. Manhattan plot of *BnaA05.FAD7* eGWAS at 40 DAF. Fig. S4. Study design on ATAC-seq of 6 representative accessions of *B. napus*. Fig. S5. Correlation analysis of 59 ATAC-seq samples. The samples are named according to “(22, 26, 34 or 40) DAF” + “accession_cellular ploidy (2C, 3C or 4C)” + “biological replicate” format naming. Fig. S6. Regional plot of ATAC-seq data and eGWAS results of *BnaA08.TGD1*. *BnaA08.TGD1* is marked by a dashed line. The shaded area indicates the lead SNP of the local eQTL affecting *BnaA08.TGD1*. Fig. S7. Comparison of the explained variance (*r2*) of eQTLs for expression variation in or not in OCRs. Fig. S8. Expression correlation analysis of adjacent genes and randomly sampled gene pairs. Violin plot shows that the expression correlation of adjacent genes is significantly higher than that of randomly sampled gene pairs, *** indicates *P* < 0.001 in Kolmogorov-Smirnov test. Fig. S9. eQTL localization of *B. napus*. (a) Dot plot showing eQTL and their regulated genes in 19 chromosomes. *x*-axis indicates the physical position of each variant on the ZS11 genome. *y*axis indicates the physical position of the localized gene on the ZS11 genome and each point indicates a detected eQTL locus. Points on the diagonal line indicate local eQTLs and points away from the diagonal line indicate distant eQTLs. (b) Average number of eQTL for different gene types. Fig. S10. The propensity of genome-wide homoeologous gene expression on different chromosomes of *An* and *Cn* at 40 DAF. Fig. S11. Effect of local eQTLs on asymmetric regulation (*Cn* gene regulating *An* gene) of subgenomes. Fig. S12. Comparison of gene correlations in different types of aHGPs at 40 DAF. “BG” represents all HGPs. “Local” represents aHGPs with local eQTLs and “No Local” represents aHGPs without local eQTLs. Fig. S13. The density map shows the enrichment of TFs in HGPs with feedback regulation. The blue inverted triangle represents 333 HGPs that are TFs. Fig. S14. The density plot shows the maximum number of eQTLs within 1 Mb. We defined the number of eQTLs within 1 Mb greater than 81 (*P* < 0.01) as a hotspot (red dot). Fig. S15. Characterization of SOC-related gene regulation in subgenomic imbalance. a Comparison of SOC-related gene correlations in different types of aHGPs at 20 DAF. “BG” represents all HGPs. “Local” represents aHGPs with local eQTLs and “No Local” represents aHGPs without local eQTLs. b Correlation between expression levels of *BnaA02.PDAT1* and *BnaC02.PDAT1* at 20 DAF. c Correlation between expression levels of *BnaA01.AAD5* and *BnaC01.AAD5* at 20 DAF. d Correlation between expression levels of *BnaA07.WRI1* and *BnaC06.WRI1* at 20 DAF. Fig. S16. The enrichment of hotspots regulating TWAS significant genes of SOC. The *x*-axis and *y*-axis represent enrichment of TWAS significant genes of SOC in hotspots at 20 DAF and 40 DAF. Fig. S17. Workflow of XGBoost module. The collected expression data of TFs were used to construct the XGBoost model, upstream TFs prediction is performed for each gene in a gene set, and finally the prediction results of the whole gene set are summarized. Fig. S18. Expression of *BnaA09.SCL31* at 20 DAF is regulated by a distant hotspot eQTL, which in turn regulates downstream SOC-related genes and affects seed oil content. Box plots of SOC and SOC-related genes (*BnaA07.NAC13*, *lysophosphatidic acid acyltransferase* (*BnaC07*.*LPAAT*), *GLYCOLIPID TRANSFER PROTEIN* (*BnaC06*.*GLPT*), *SHEWENELLA-LIKE PROTEIN PHOSPHATASE 1* (*BnaC03.SLP1*)) based on haplotypes of distant eQTL. Fig. S19. The population information of *BnaA09.SCL31* expression level at 20 DAF. a Box plots for expression levels at 20 DAF based on the haplotypes of variants in the gene region and the upstream 2 kb region of *BnaA09.SCL31*. b Correlation between SOC and expression levels of *BnaA09.SCL31* at 20 DAF. Fig. S20. Identification of Arabidopsis T-DNA Mutants of *SCL31*. (a) Schematic diagram of TDNA insertion sites in *SCL31* mutants (*scl31-1*, *scl31-2*). (b) Identification of homozygous TDNA mutants by PCR with a pair of gene specific primers (LP+RP) or combination of T-DNA border primer (LB1.3) and gene specific primers (RP). (c) Expression of *SCL31* in WT, *scl31-1*, *scl31-2* analyzed by quantitative RT-PCR using RNA extracted from leaves. Values are means(SD) (*n* = 3 biological repeats). ** indicates *P* < 0.01 and *** indicates *P* < 0.001 compared with WT in Student’s *t* test. Fig. S21. Fatty acid composition of *SCL31* mutant seeds (*scl31-1*, *scl31-2*). Values are means(SD) (*n* = 5 biological repeats). * indicates *P* < 0.05 and ** indicates *P* < 0.01 compared with WT in Student’s *t* test. Fig. S22. Silique and seed of *SCL31* mutant lines (*scl31-1*, *scl31-2*). (a) Silique of *scl31-1* and *scl31-2*. Bar = 2mm. (b) Seed of *scl31-1* and *scl31-2*. Bar = 500𝜇m. (c) Seed length of *scl31-1* and *scl31-2*. Values are means(SD) (n = 5 biological repeats) and different letters indicate differences at *P* < 0.05 using Student’s *t* test. (d) Seed width of *scl31-1* and *scl31-2*. Values are means(SD) (*n* = 5 biological repeats) and different letters indicate differences at *P* < 0.05 using Student’s *t* test. Fig. S23. Workflow of Basenji module. ATAC-seq data are used to identify OCRs and construct basenji models. Subsequently, 2 kb promoter sequences of genes in the gene set are collected for key motif identification. Fig. S24. The performance metrics of the Basenji deep learning model trained with the ZS11 reference genome and 59 rapeseed ATAC-seq samples. The horizontal axis represents 59 different samples and the vertical axis represents the performance data of the corresponding fold (where *r2* represents the square of the correlation. AUPRC is the accuracy between the predicted regression value and the true value using the precision score function in scikit-learn package to calculate the accuracy between the predicted regression value and the true value; AUROC is the area under the precision-recall curve of the predicted regression value and the true value using the roc_auc_score function in the in scikit-learn package). Fig. S25. The correlation between *BnaA07.NAC13* and the expression of the downstream genes potentially regulated by *BnaA07.NAC13*. Fig. S26. Transcriptional regulation of *BnaC04.MYC70* and BnaC02G0181500ZS are activated by *BnaA07.NAC13*. a Schematic representation of the constructs used for the dual-luciferase assay. The effector constructs contain *BnaA07.NAC13* driven by the CaMV35S promoter. The reporter construct contains the firefly luciferase driven by *BnaC04.MYC70* and BnaC02G0181500ZS promoter, and the Renilla luciferase (REN) driven by the CaMV35S promoter. And the black square is the terminator. b,c Bar graph showing the relative LUC/REN ratio in the dual-luciferase assay. Values are means(SD) (*n* = 3 biological repeats). Fig. S27. Expression of *BnaA07.NAC13* and *BnaC07.NAC13* in WT and OE lines analyzed by quantitative RT-PCR using RNA extracted from leaves. (a, b) Values are means(SD) (*n* = 3 biological repeats). *** indicates *P* < 0.001 compared with WT in Student’s *t* test. Fig. S28. Fatty acid composition phenotype of OE lines (*BnaA07.NAC13*, *BnaC07.NAC13*). (a, b) Values are means(SD) (*n* = 5 biological repeats). * indicates *P* < 0.05, ** indicates *P* < 0.01 and *** indicates *P* < 0.001 compared with WT in Student’s *t* test. Fig. S29. Manhattan plot *of BnaA07.NAC13* eGWAS at 20 DAF and 40 DAF. Fig. S30. Correlation between expression levels of *BnaA07.NAC13* and TFs (such as *BnaA09.SCL31* , *WIP DOMAIN PROTEIN 3* (*BnaA09.WIP3*), *CRY2-INTERACTING BHLH 4* (*BnaA09.CIB4*), *BLUEJAY* (*BnaA09.BLJ*)) in Hotspot87-88 at 20 DAF and 40 DAF. Fig. S31. Correlation between expression levels of *BnaA09.SCL31* and *BnaA07.NAC13* at 20 DAF and 40 DAF. Fig. S32. Size and number of LDs expressing BnaA09.SCL31 and BnaA07.NAC13s in tobacco leaves. a LD count expressing BnaA09.SCL31 and BnaA07.NAC13s by size (average diameter). Values are means(SD) (*n* = 3 biological repeats). Different letters indicate significant difference at *P* < 0.05, as determined by one-way ANOVA with Tukey’s post-test. b Quantification of LD sizes in leaf mesophyll cells. Circles represent size of LDs. Fig. S33. Expression of *NAC13, SUD1, LPAAT, SCL30, SLP1* in WT, *scl31-1*, *scl31-2* analyzed by quantitative RT-PCR using RNA extracted from leaves. (a-e) Values are means(SD) (*n* = 3 biological repeats). “NS” indicates *P* > 0.05, * indicates *P* < 0.05, ** indicates *P* < 0.01 and *** indicates *P* < 0.001 compared with WT in Student’s *t* test. Fig. S34. Transcriptional regulation of genes are activated by *BnaA09.SCL31* or *BnaA07.NAC13*. a Schematic representation of the constructs used for the dual-luciferase assay. The effector constructs contain *BnaA09.SCL31* and *BnaA07.NAC13* driven by the CaMV35S promoter, respectively. The reporter construct contains the firefly luciferase driven by *BnaA07.NAC13*, *BnaC07.LPAAT*, *BnaC03.SLP1* and *BnaA07.SRO3* promoter, and the Renilla luciferase (REN) driven by the CaMV35S promoter. And the black square is the terminator. b Bar graph showing the relative LUC/REN ratio in the dual-luciferase assay. Values are means(SD) (*n* = 3 biological repeats). *BnaA07.SRO3* (*SIMILAR TO RCD ONE 3*).

Additional file 2: Table S1. Genome-wide eQTLs identified at 20 DAF. Table S2. Genome-wide eQTLs identified at 40 DAF. Table S3. Genome-wide eGene-eQTLs identified at 20 DAF. Table S4. Genome-wide eGene-eQTLs identified at 40 DAF. Table S5. Results of GO enrichment analysis of specifically identified eGenes at 20 DAF. Table S6. Results of GO enrichment analysis of specifically identified eGenes at 40 DAF. Table S7. Summary of ATAC-Seq data. Table S8. List of homoeologous genes among subgenomes. Table S9. List of homoeologous genes on gene expression trend and regulation at 20 DAF. Table S10. List of homoeologous genes on gene expression trend and regulation at 40 DAF. Table S11. The information of hotspots at 20 DAF. Table S12. The information of hotspots at 40 DAF. Table S13. The list of TWAS significant genes of SOC and SGC at 20 DAF. Table S14. The list of TWAS significant genes of SOC and SGC at 40 DAF. Table S15. Enrichment analysis of eQTL hotspot regulatory genes in TWAS significant genes of SOC at 20 DAF. Table S16. Enrichment analysis of eQTL hotspot regulatory genes in TWAS significant genes of SOC at 40 DAF. Table S17. The results of Tomtom analysis of key sequences identified by Basenji module. Table S18. Primers for gene cloning and PCR confirmation.

Additional file 3. Review history.

## Data Availability

The genome resequencing data of 505 *B. napus* accessions are available under Genome Sequence Archive (GSA; https://bigd.big.ac.cn/gsa/) with Bioproject ID PRJCA002835 [99], and the 583 seed transcriptome data are available under GSA with Bioproject ID PRJCA002836 [100]. The 273 time-series and multi-tissue transcriptome data are available under the BnTIR database [101]. All the ATAC-seq raw sequencing data generated in this study are available in the GSA with Bioproject ID PRJCA009262 [102]. All the materials in this study are available upon request.
