# Supplementary material for: Comprehensive transcriptional variability analysis reveals gene networks regulating seed oil content of Brassica napus
Source: Genome Biol. 2022 Nov 7;23:233. doi: 10.1186/s13059-022-02801-z (PMC9639296; doi:10.1186/s13059-022-02801-z)
Supplement: Supplementary file 1 — Additional file 1: Fig. S1. Overview of experimental and research analysis methods. Fig. S2. Venn diagram of the distribution of genes regulated by different types of eQTLs (local eQTL and distant eQTL) at 20 DAF and 40 DAF. (a) Distribution number of genes which were regulated by different types of eQTLs at 20 DAF. (b) Distribution number of genes which were regulated by different types of eQTLs at 40 DAF. Fig. S3. Manhattan plot of BnaA05.FAD7 eGWAS at 40 DAF. Fig. S4. Study design on ATAC-seq of 6 representative accessions of B. napus. Fig. S5. Correlation analysis of 59 ATAC-seq samples. The samples are named according to “(22, 26, 34 or 40) DAF” + “accession_cellular ploidy (2C, 3C or 4C)” + “biological replicate” format naming. Fig. S6. Regional plot of ATAC-seq data and eGWAS results of BnaA08.TGD1. BnaA08.TGD1 is marked by a dashed line. The shaded area indicates the lead SNP of the local eQTL affecting BnaA08.TGD1. Fig. S7. Comparison of the explained variance (r2) of eQTLs for expression variation in or not in OCRs. Fig. S8. Expression correlation analysis of adjacent genes and randomly sampled gene pairs. Violin plot shows that the expression correlation of adjacent genes is significantly higher than that of randomly sampled gene pairs, *** indicates P < 0.001 in Kolmogorov-Smirnov test. Fig. S9. eQTL localization of B. napus. (a) Dot plot showing eQTL and their regulated genes in 19 chromosomes. x-axis indicates the physical position of each variant on the ZS11 genome. yaxis indicates the physical position of the localized gene on the ZS11 genome and each point indicates a detected eQTL locus. Points on the diagonal line indicate local eQTLs and points away from the diagonal line indicate distant eQTLs. (b) Average number of eQTL for different gene types. Fig. S10. The propensity of genome-wide homoeologous gene expression on different chromosomes of An and Cn at 40 DAF. Fig. S11. Effect of local eQTLs on asymmetric regulation (Cn gene regulating An gene [file 13059_2022_2801_MOESM1_ESM.pdf]

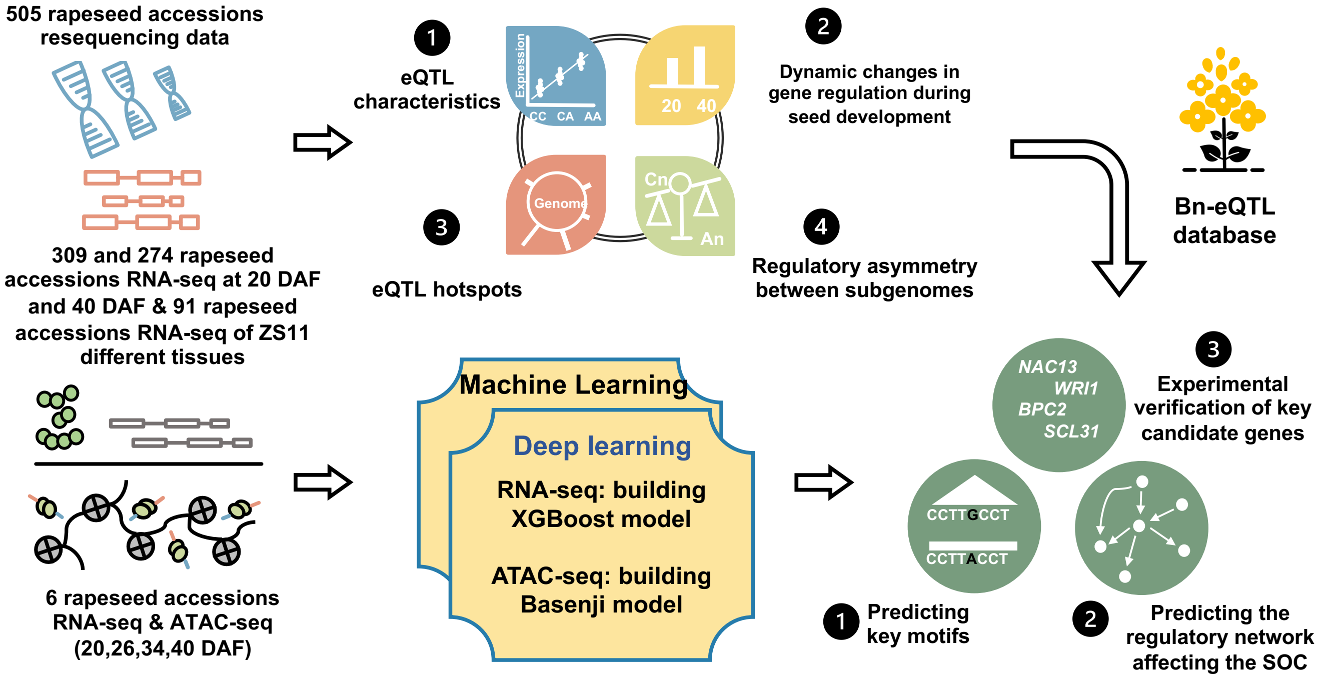

Fig. S1 Overview of experimental and research analysis methods.

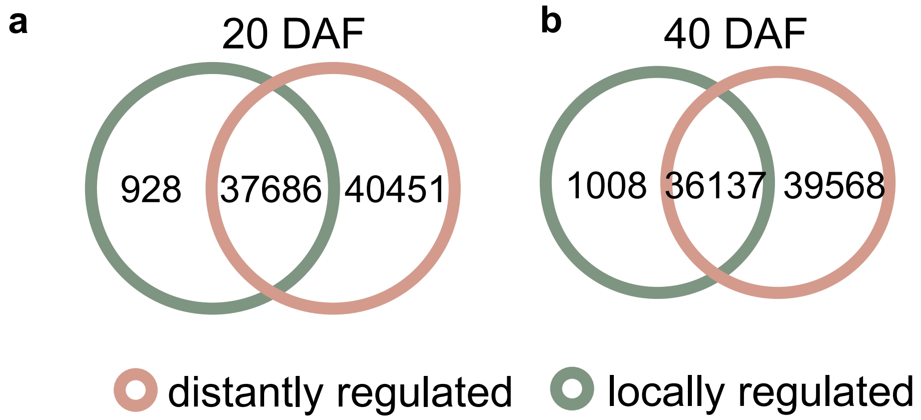

Fig. S2 Venn diagram of the distribution of genes regulated by different types of eQTLs (local eQTL and distant eQTL) at 20 DAF and 40 DAF. (a) Distribution number of genes which were regulated by different types of eQTLs at 20 DAF. (b) Distribution number of genes which were regulated by different types of eQTLs at 40 DAF.

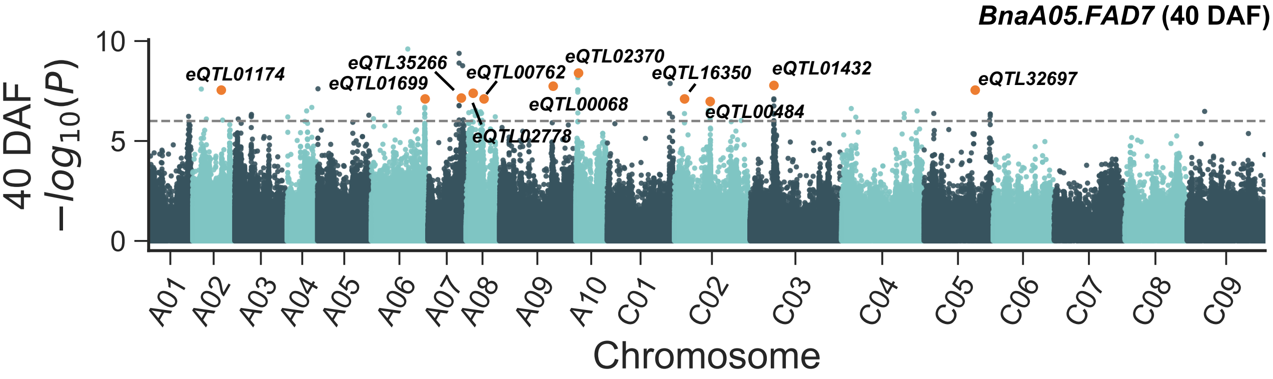

Fig. S3 Manhattan plot of *BnaA05.FAD7* eGWAS at 40 DAF.

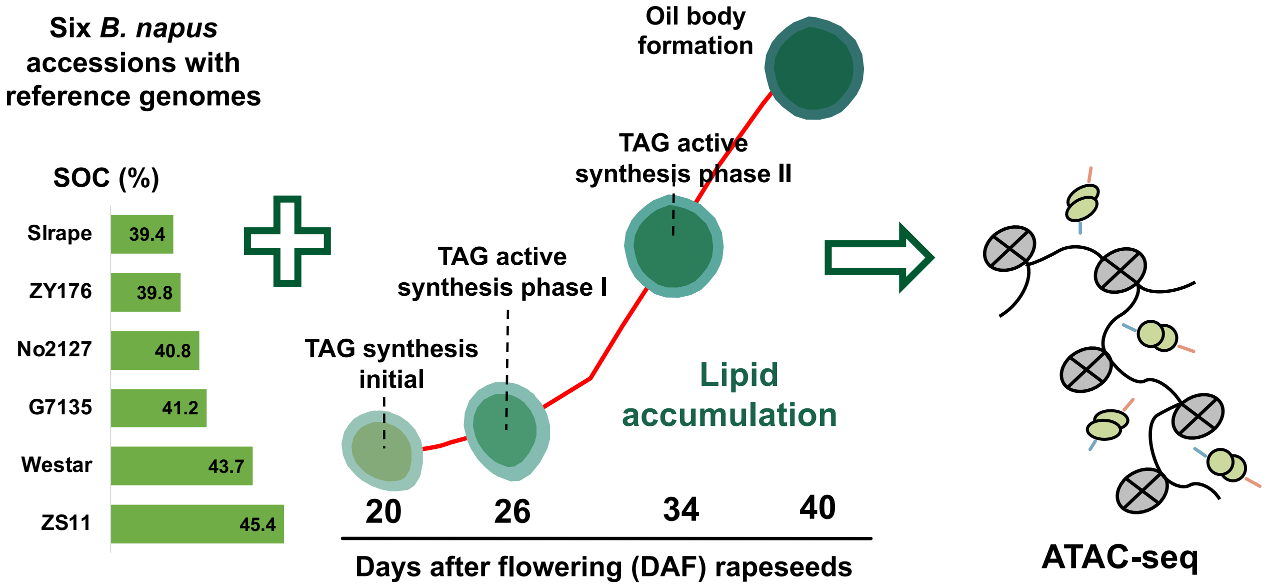

Fig. S4 Study design on ATAC-seq of 6 representative accessions of *B. napus*.

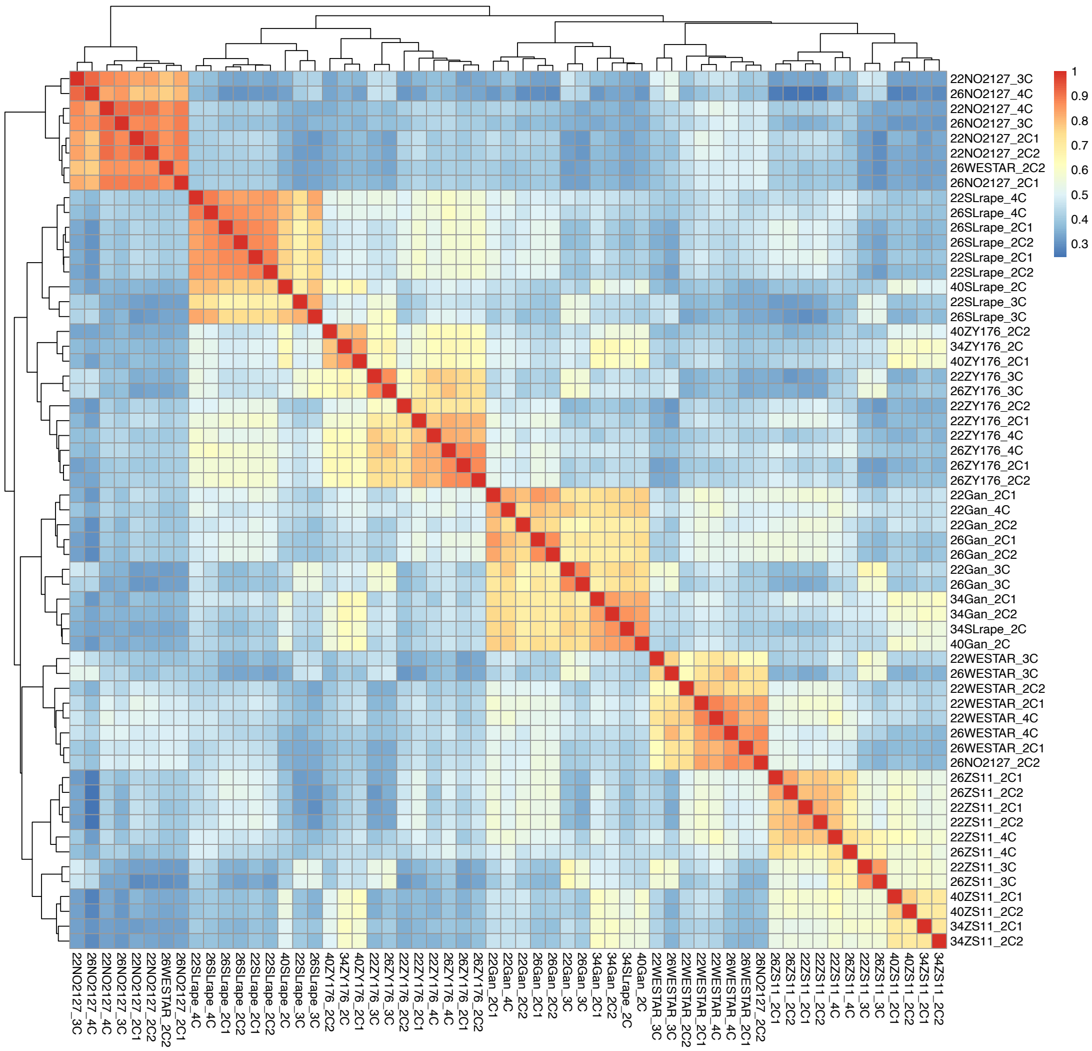

Fig. S5 Correlation analysis of 59 ATAC-seq samples. The samples are named according to "(22, 26, 34 or 40) DAF" + "accession_cellular ploidy (2C, 3C or 4C)" + "biological replicate" format naming.

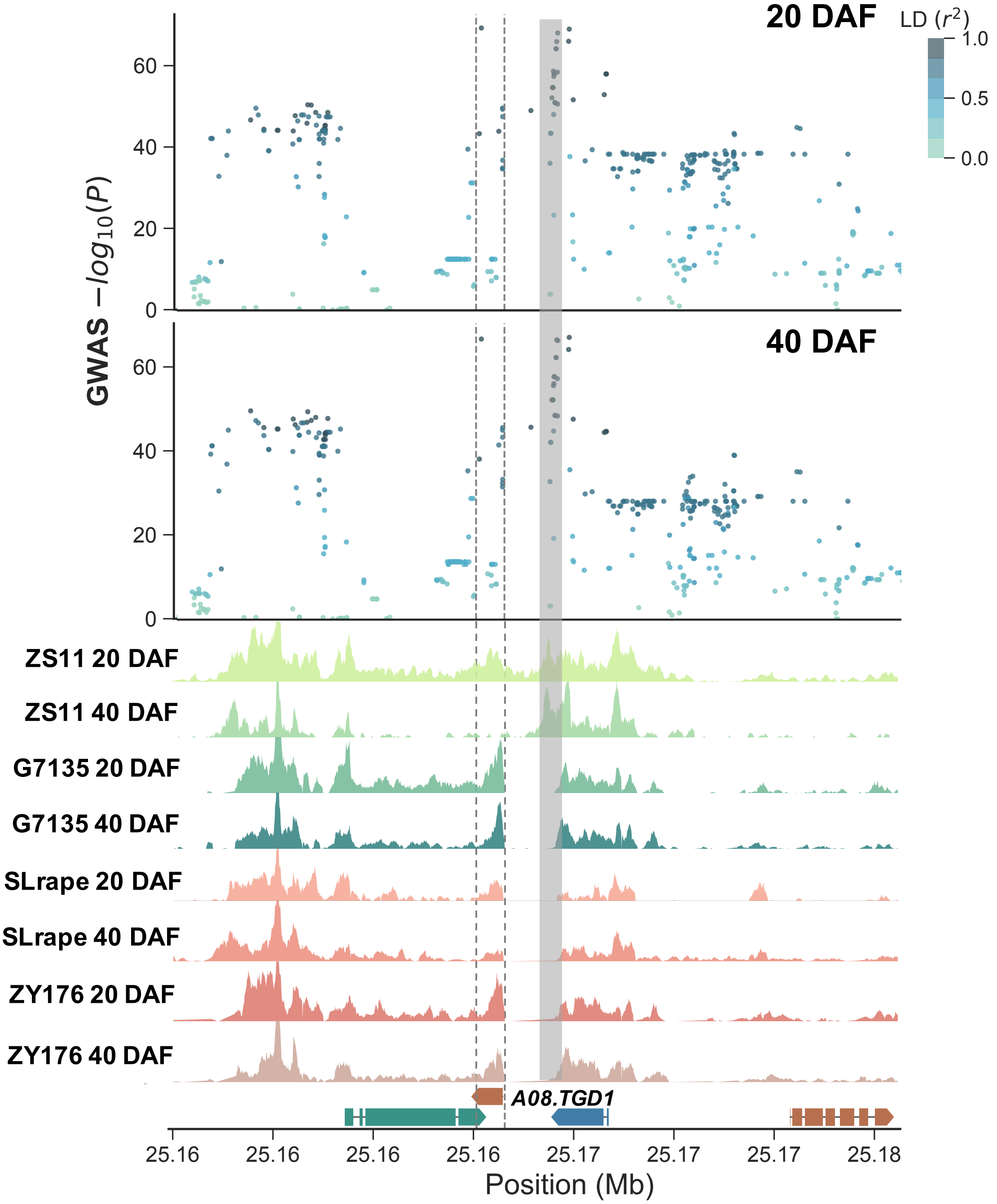

Fig. S6 Regional plot of ATAC-seq data and eGWAS results of *BnaA08.TGD1*. *BnaA08.TGD1* is marked by a dashed line. The shaded area indicates the lead SNP of the local eQTL affecting *BnaA08.TGD1*.

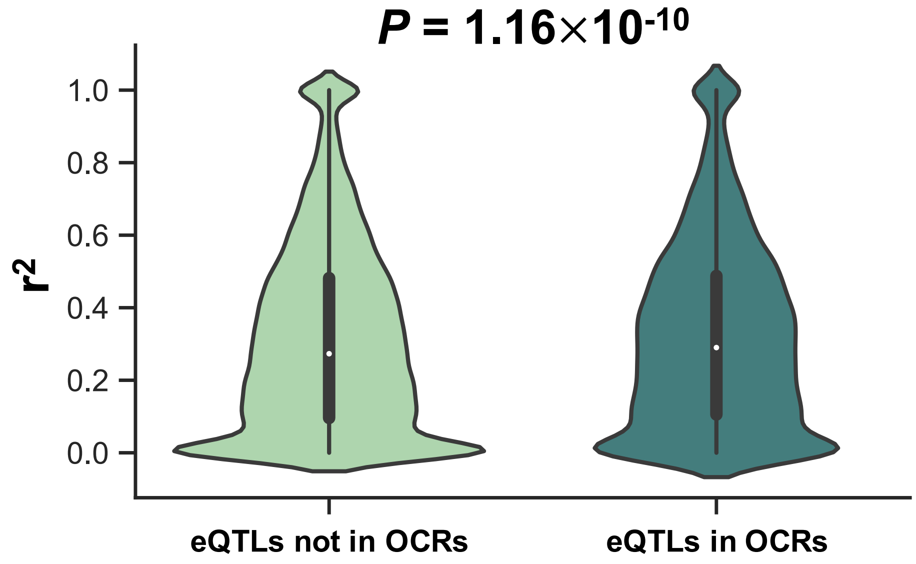

Fig. S7 Comparison of the explained variance (r^2) of eQTLs for expression variation in or not in OCRs.

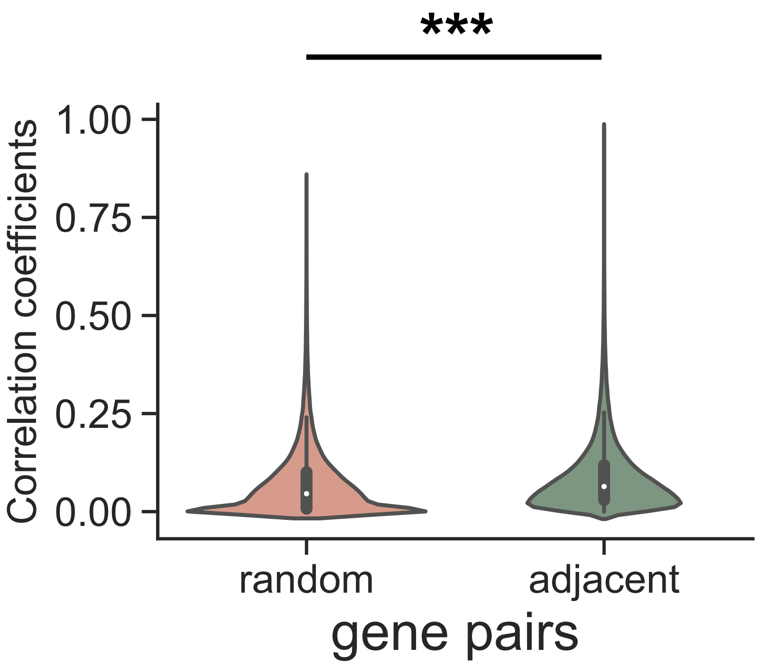

Fig. S8 Expression correlation analysis of adjacent genes and randomly sampled gene pairs. Violin plot shows that the expression correlation of adjacent genes is significantly higher than that of randomly sampled gene pairs, *** indicates $P < 0.001$ in Kolmogorov-Smirnov test.

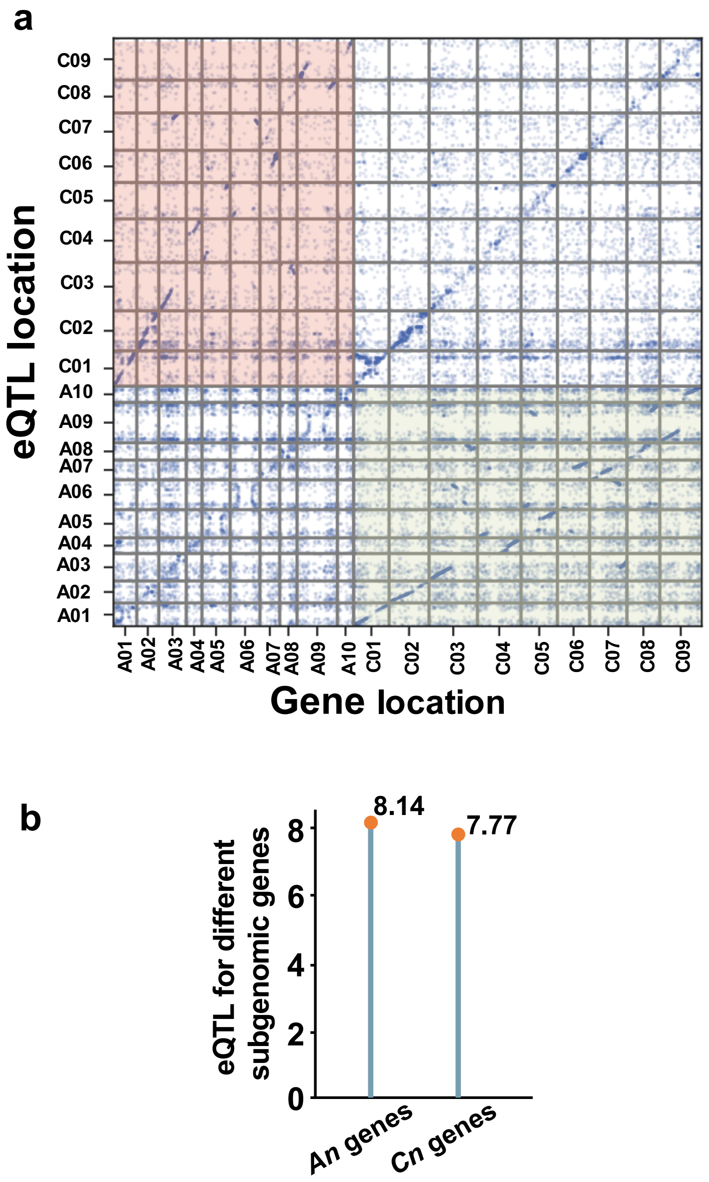

Fig. S9 eQTL localization of *B. napus*. (a) Dot plot showing eQTL and their regulated genes in 19 chromosomes. *x*-axis indicates the physical position of each variant on the ZS11 genome. *y*-axis indicates the physical position of the localized gene on the ZS11 genome and each point indicates a detected eQTL locus. Points on the diagonal line indicate local eQTLs and points away from the diagonal line indicate distant eQTLs. (b) Average number of eQTL for different gene types.

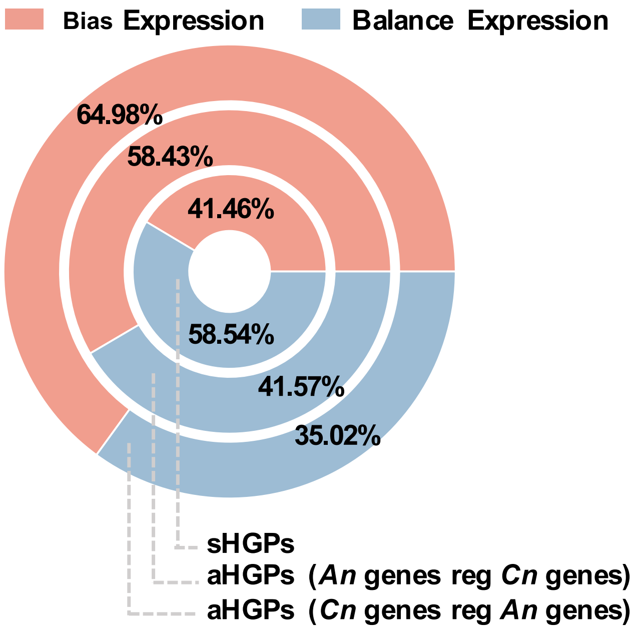

Fig. S10 The propensity of genome-wide homoeologous gene expression on different chromosomes of *An* and *Cn* at 40 DAF.

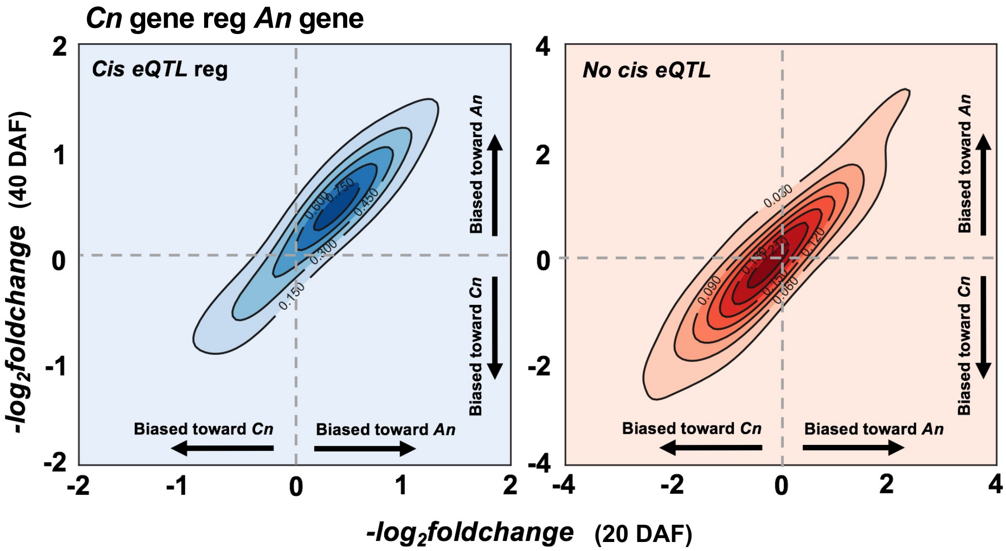

Fig. S11 Effect of local eQTLs on asymmetric regulation (*Cn* gene regulating *An* gene) of subgenomes.

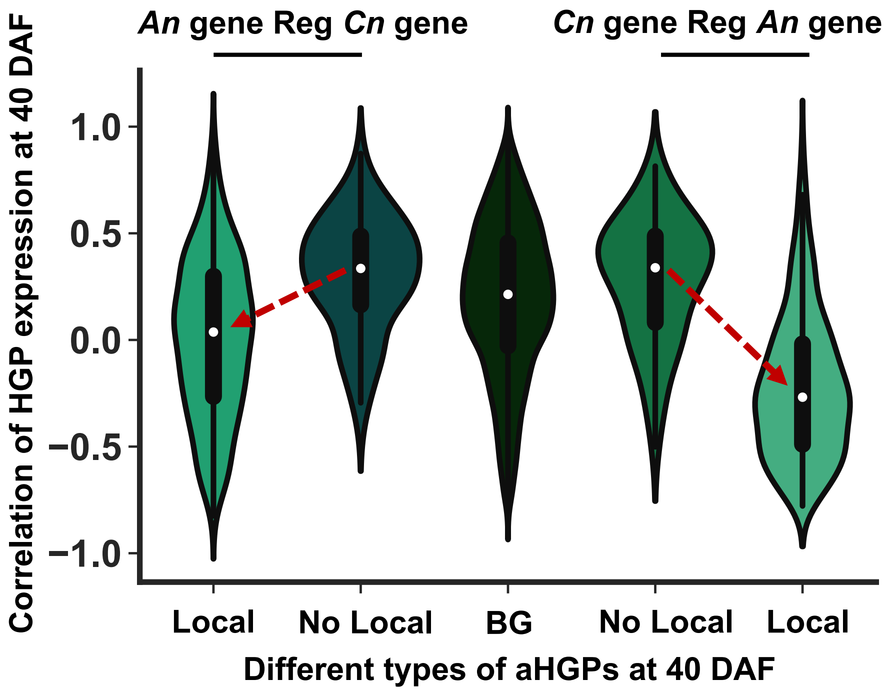

Fig. S12 Comparison of gene correlations in different types of aHGPs at 40 DAF. "BG" represents all HGPs. "Local" represents aHGPs with local eQTLs and "No Local" represents aHGPs without local eQTLs.

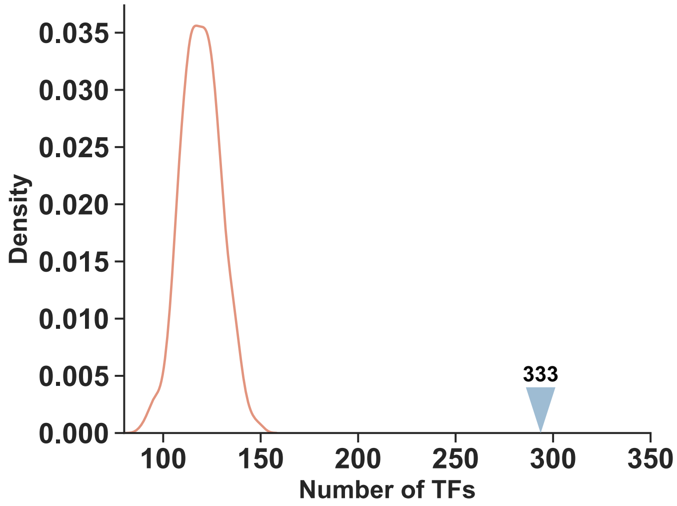

Fig. S13 The density map shows the enrichment of TFs in HGPs with feedback regulation. The blue inverted triangle represents 333 HGPs that are TFs.

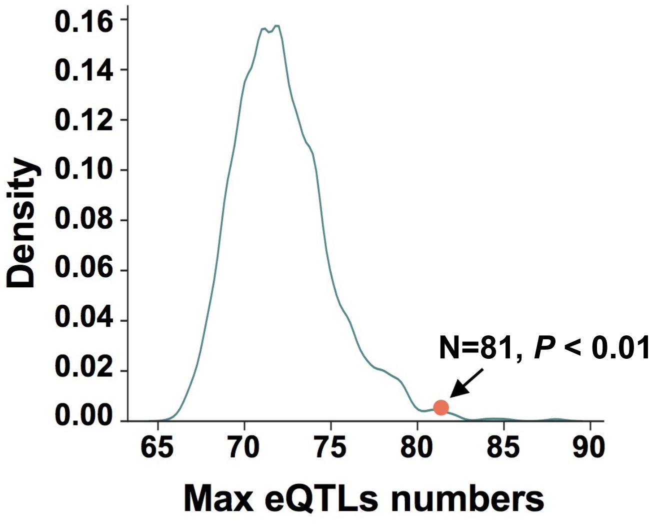

Fig. S14 The density plot shows the maximum number of eQTLs within 1 Mb. We defined the number of eQTLs within 1 Mb greater than 81 ($P < 0.01$) as a hotspot (red dot).

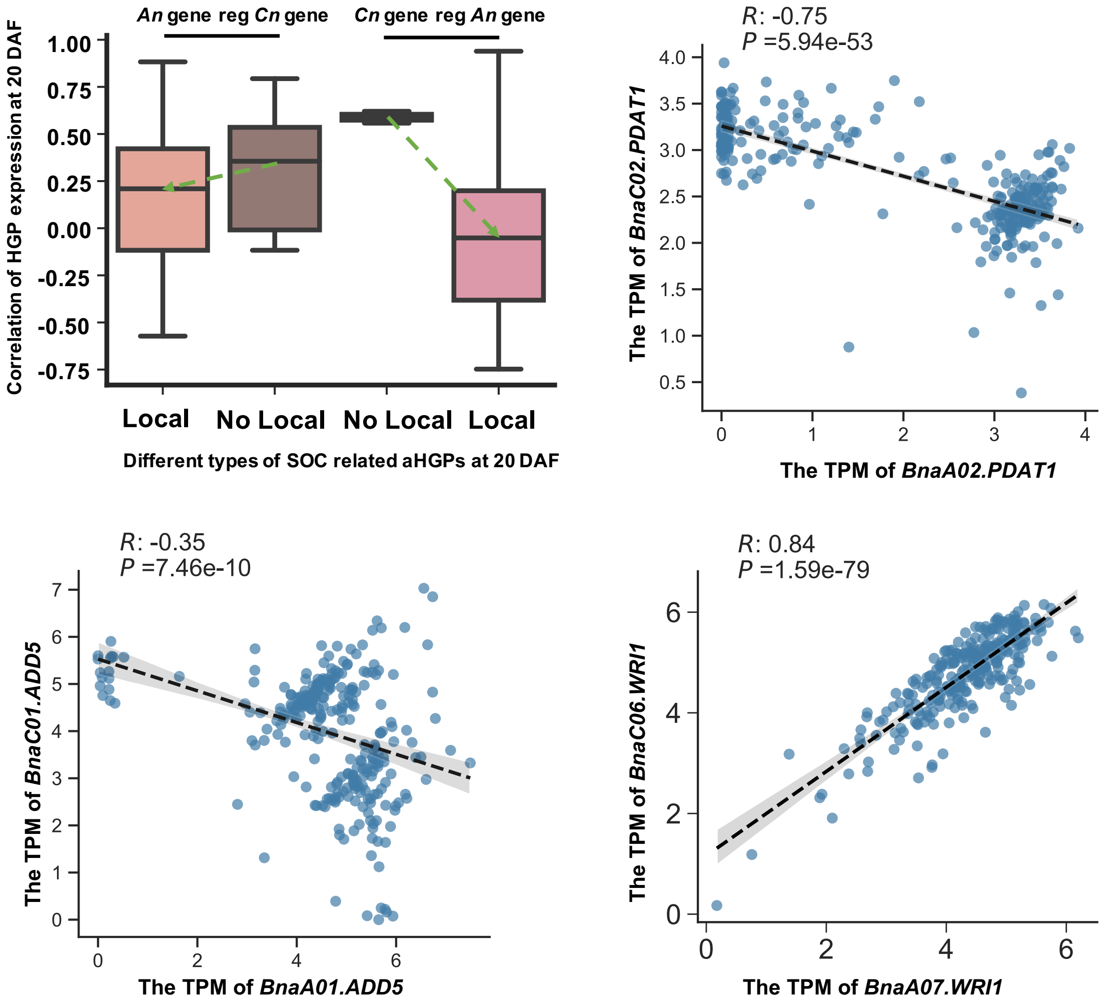

Fig. S15 Characterization of SOC-related gene regulation in subgenomic imbalance. **a** Comparison of SOC-related gene correlations in different types of aHGPs at 20 DAF. "BG" represents all HGPs. "Local" represents aHGPs with local eQTLs and "No Local" represents aHGPs without local eQTLs. **b** Correlation between expression levels of *BnaA02.PDAT1* and *BnaC02.PDAT1* at 20 DAF. **c** Correlation between expression levels of *BnaA01.AAD5* and *BnaC01.AAD5* at 20 DAF. **d** Correlation between expression levels of *BnaA07.WRI1* and *BnaC06.WRI1* at 20 DAF.

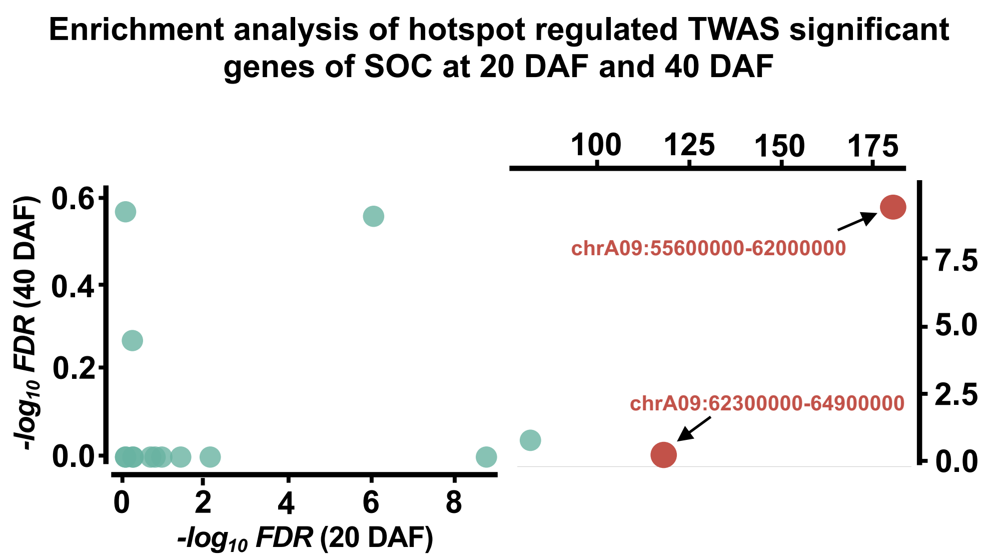

Fig. S16 The enrichment of hotspots regulating TWAS significant genes of SOC. The *x*-axis and *y*-axis represent enrichment of TWAS significant genes of SOC in hotspots at 20 DAF and 40 DAF.

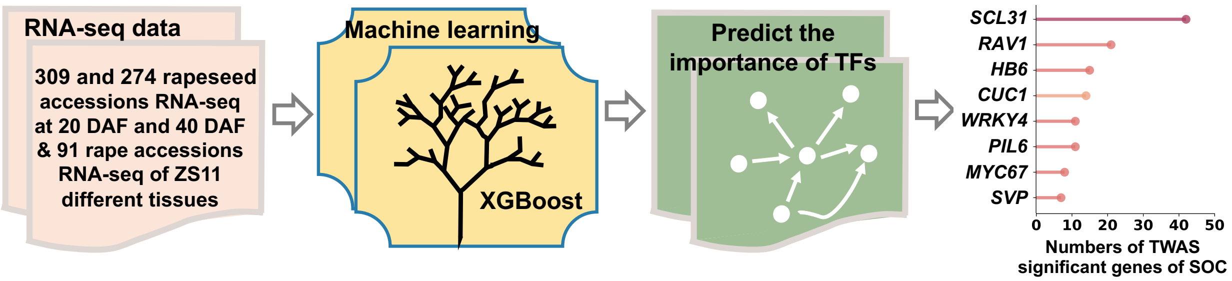

Fig. S17 Workflow of XGBoost module. The collected expression data of TFs were used to construct the XGBoost model, upstream TFs prediction is performed for each gene in a gene set, and finally the prediction results of the whole gene set are summarized.

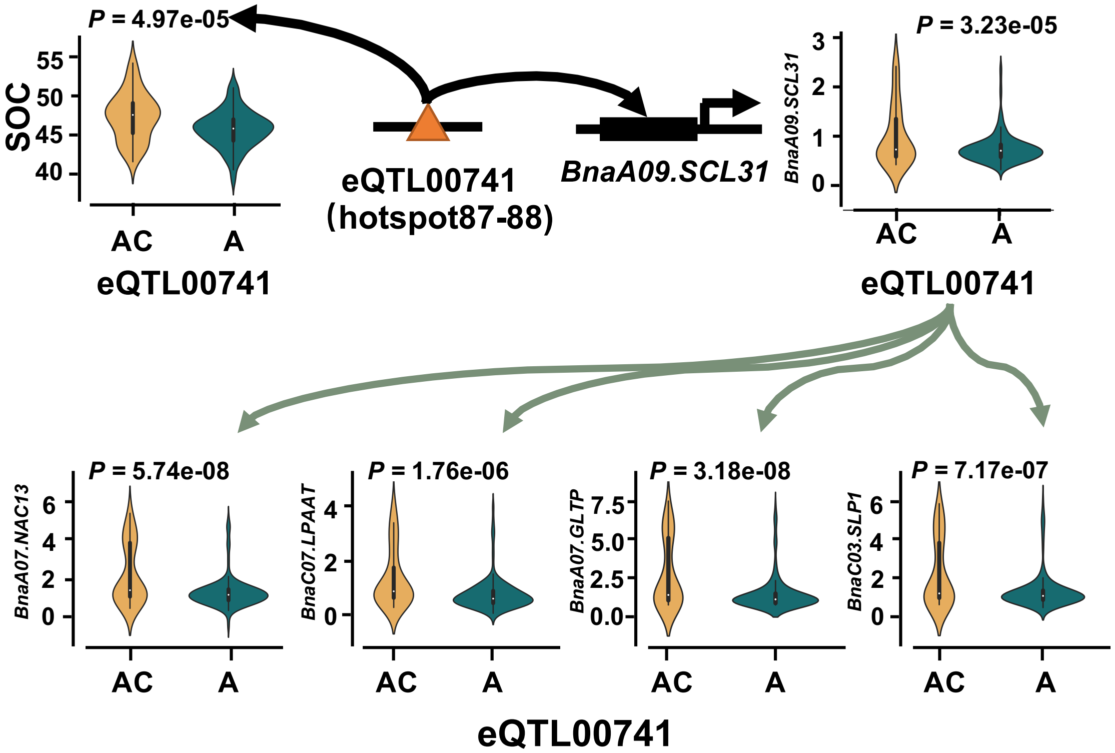

Fig. S18 Expression of *BnaA09.SCL31* at 20 DAF is regulated by a distant hotspot eQTL, which in turn regulates downstream SOC-related genes and affects seed oil content. Box plots of SOC and SOC-related genes (*BnaA07.NAC13*, *lysophosphatidic acid acyltransferase* (*BnaC07.LPAAT*), *GLYCOLIPID TRANSFER PROTEIN* (*BnaC06.GLPT*), *SHEWENELLA-LIKE PROTEIN PHOSPHATASE 1* (*BnaC03.SLP1*)) based on haplotypes of distant eQTL.

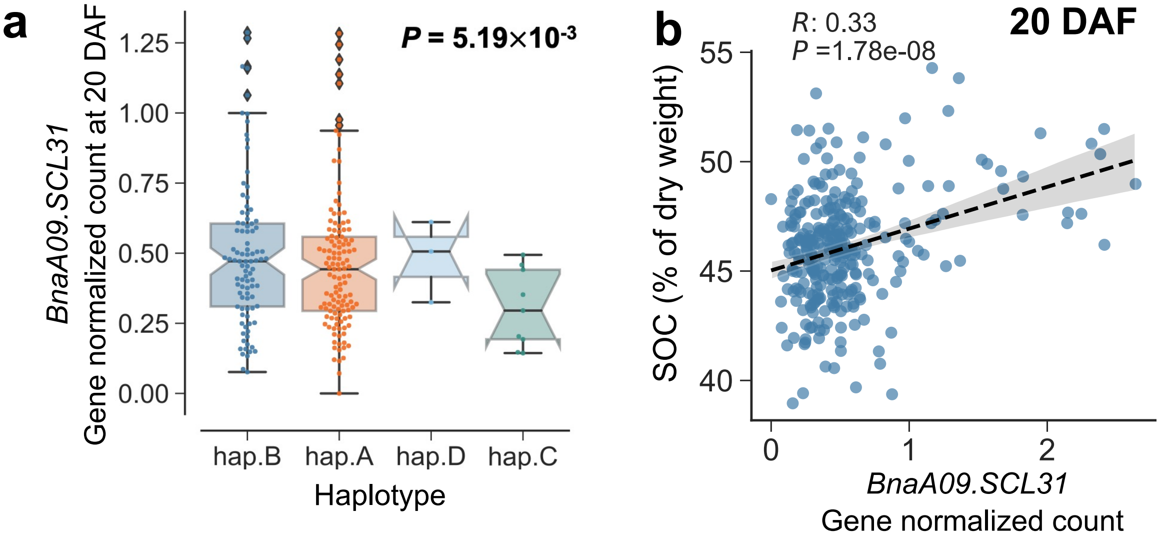

Fig. S19 The population information of *BnaA09.SCL31* expression level at 20 DAF. **a** Box plots for expression levels at 20 DAF based on the haplotypes of variants in the gene region and the upstream 2 kb region of *BnaA09.SCL31*. **b** Correlation between SOC and expression levels of *BnaA09.SCL31* at 20 DAF.

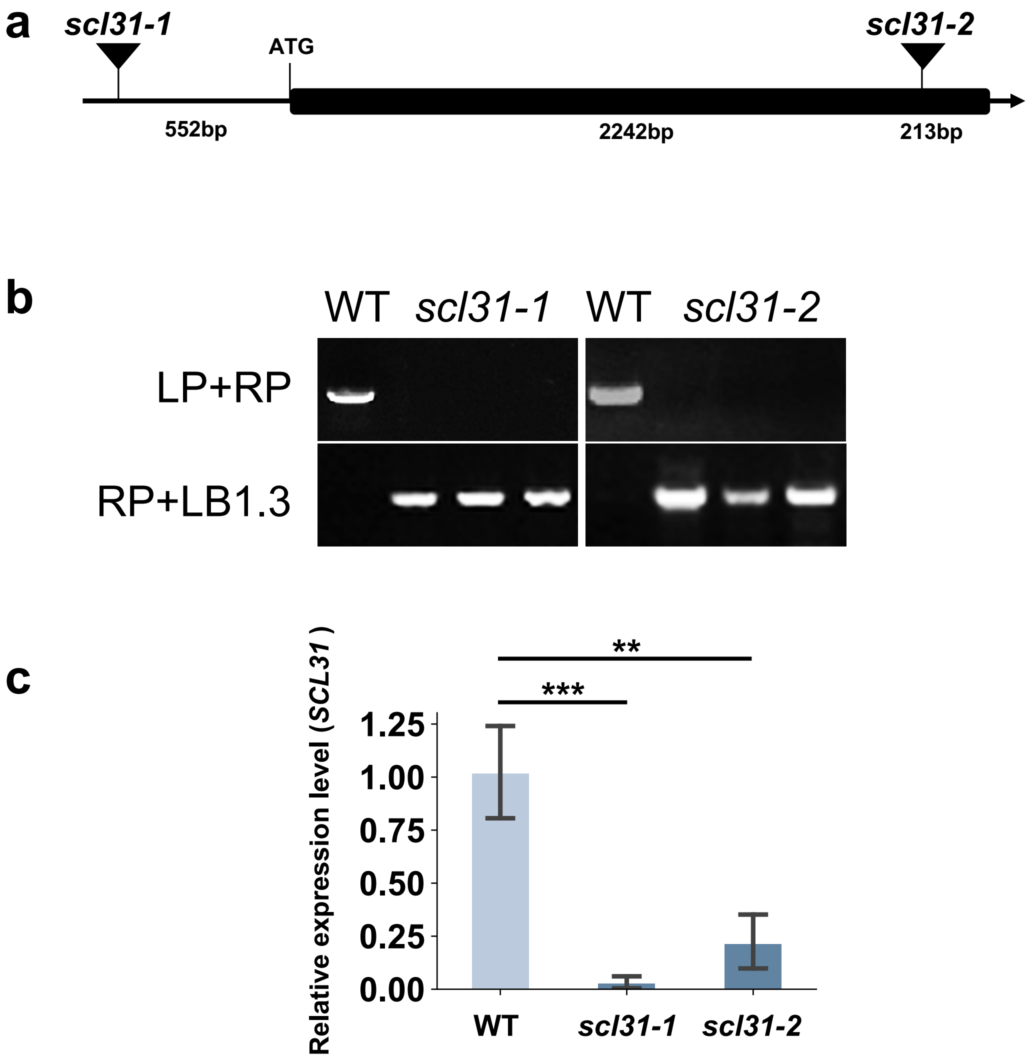

Fig. S20 Identification of Arabidopsis T-DNA Mutants of *SCL31*. (a) Schematic diagram of T-DNA insertion sites in *SCL31* mutants (*scl31-1*, *scl31-2*). (b) Identification of homozygous T-DNA mutants by PCR with a pair of gene specific primers (LP+RP) or combination of T-DNA border primer (LB1.3) and gene specific primers (RP). (c) Expression of *SCL31* in WT, *scl31-1*, *scl31-2* analyzed by quantitative RT-PCR using RNA extracted from leaves. Values are means(SD) (n = 3 biological repeats). ** indicates P < 0.01 and *** indicates P < 0.001 compared with WT in Student's t test.

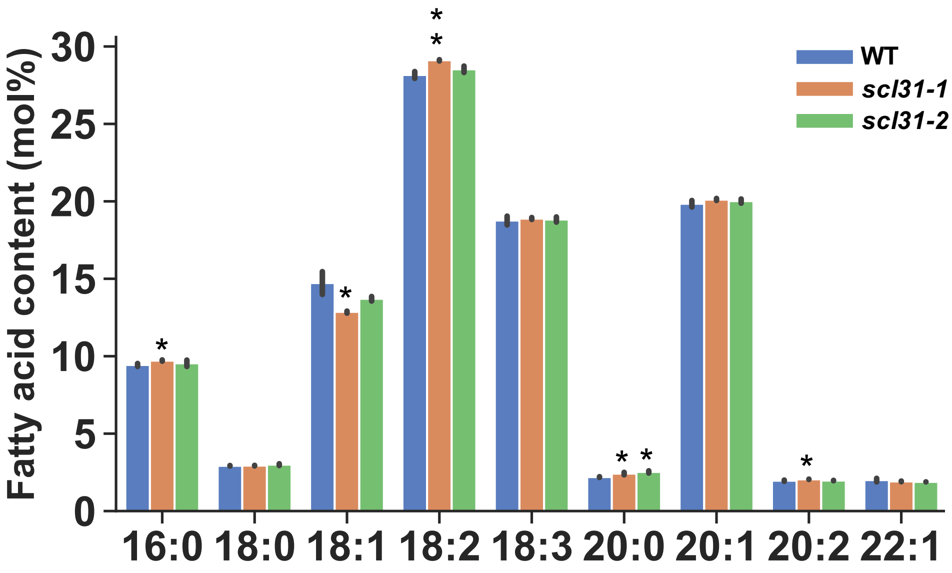

Fig. S21 Fatty acid composition of *SCL31* mutant seeds (*scl31-1*, *scl31-2*). Values are means(SD) (n = 5 biological repeats). * indicates $P < 0.05$ and ** indicates $P < 0.01$ compared with WT in Student's *t* test.

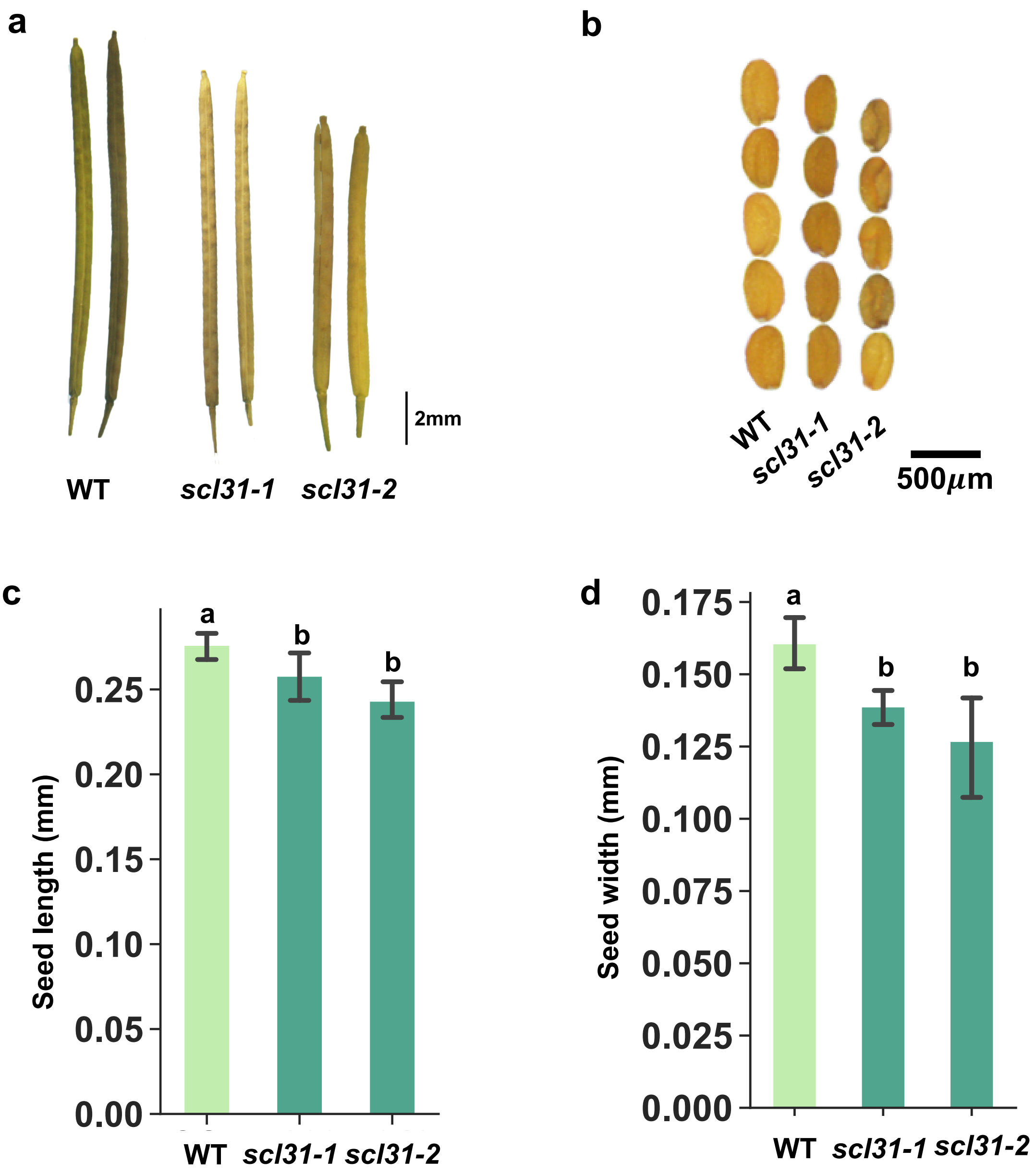

Fig. S22 Silique and seed of *SCL31* mutant lines (*scl31-1*, *scl31-2*). (a) Silique of *scl31-1* and *scl31-2*. Bar = 2mm. (b) Seed of *scl31-1* and *scl31-2*. Bar = 500μm. (c) Seed length of *scl31-1* and *scl31-2*. Values are means(SD) (n = 5 biological repeats) and different letters indicate differences at $P < 0.05$ using Student's *t* test. (d) Seed width of *scl31-1* and *scl31-2*. Values are means(SD) (n = 5 biological repeats) and different letters indicate differences at $P < 0.05$ using Student's *t* test.

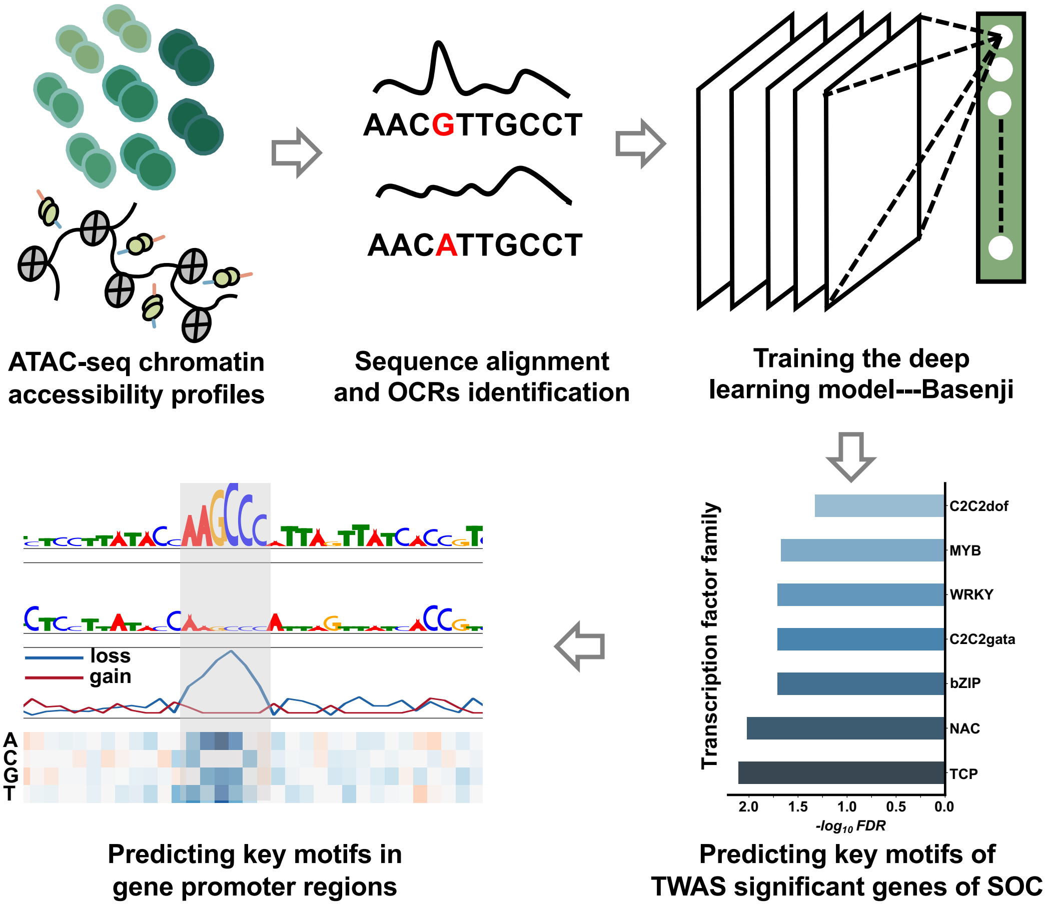

Fig. S23 Workflow of Basenji module. ATAC-seq data are used to identify OCRs and construct basenji models. Subsequently, 2 kb promoter sequences of genes in the gene set are collected for key motif identification.

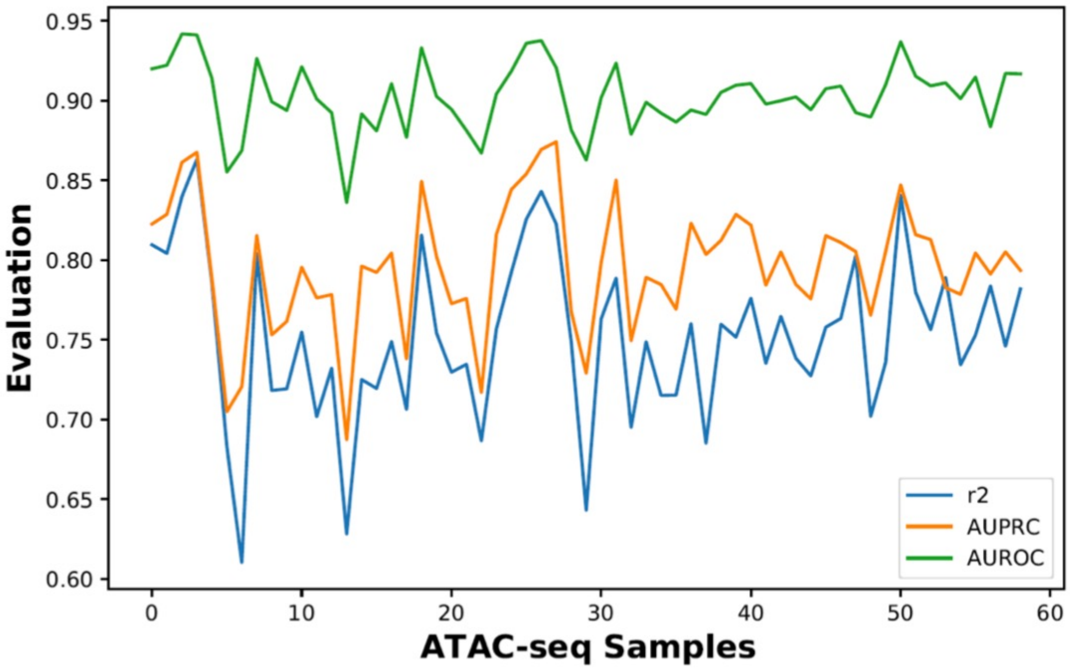

Fig. S24 The performance metrics of the Basenji deep learning model trained with the ZS11 reference genome and 59 rapeseed ATAC-seq samples. The horizontal axis represents 59 different samples and the vertical axis represents the performance data of the corresponding fold (where r^2 represents the square of the correlation. AUPRC is the accuracy between the predicted regression value and the true value using the precision score function in scikit-learn package to calculate the accuracy between the predicted regression value and the true value; AUROC is the area under the precision-recall curve of the predicted regression value and the true value using the roc_auc_score function in the in scikit-learn package).

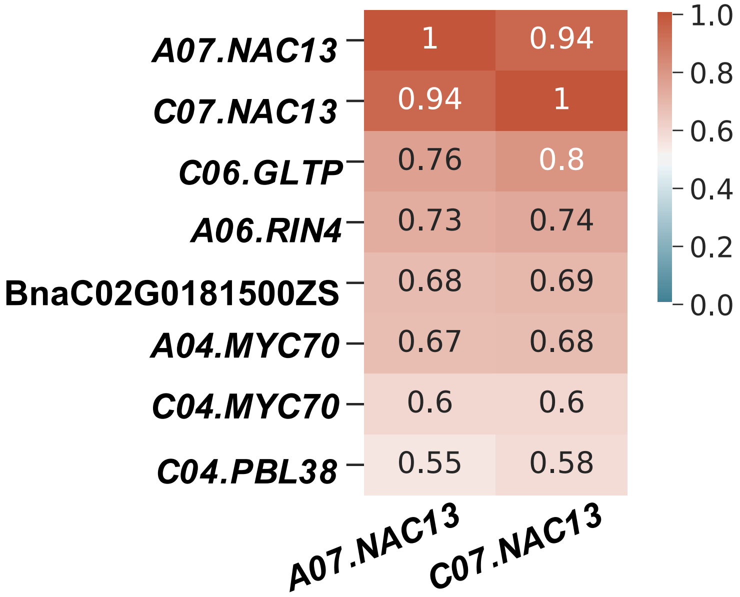

Fig. S25 The correlation between *BnaA07.NAC13* and the expression of the downstream genes potentially regulated by *BnaA07.NAC13*.

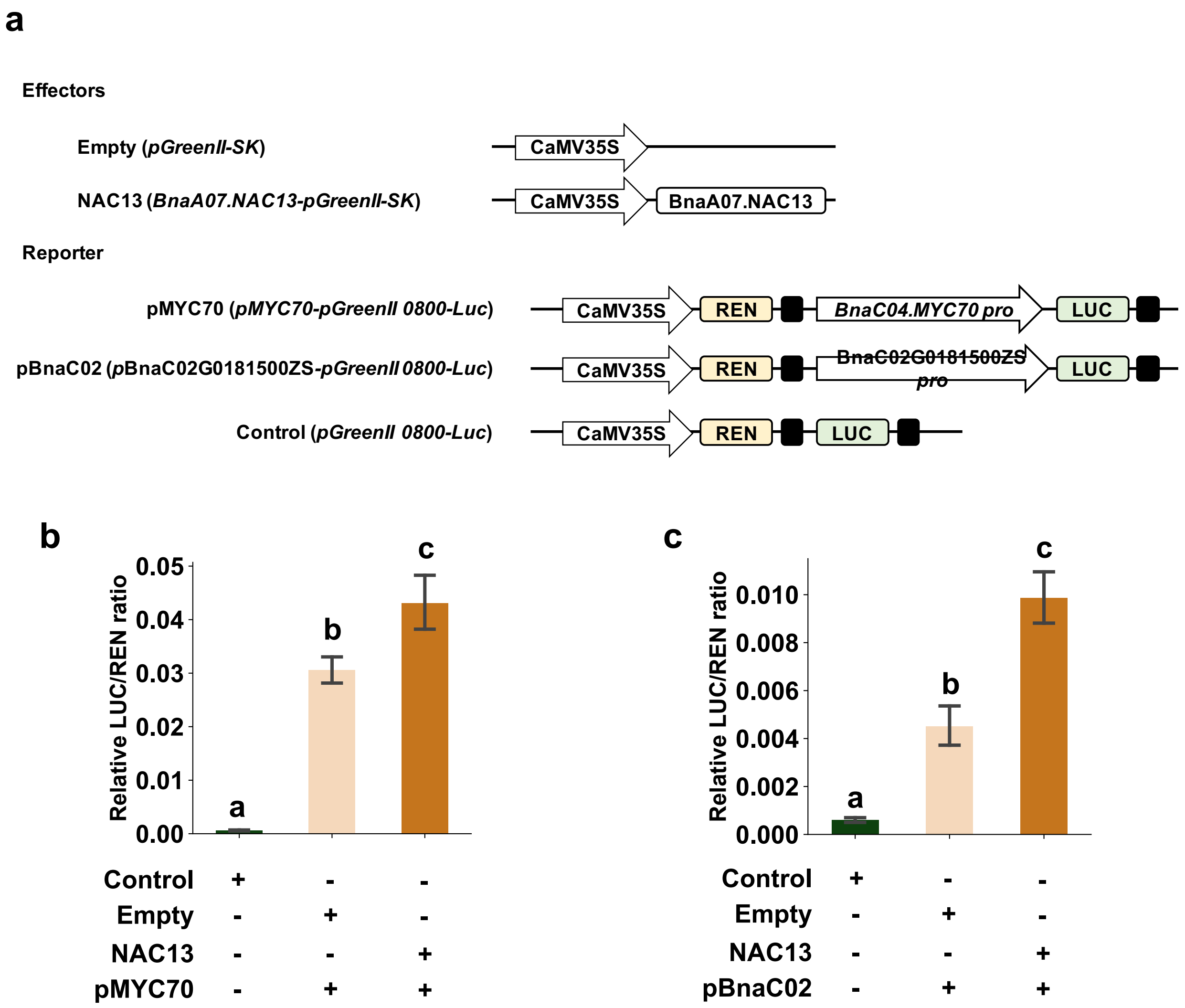

Fig. S26 Transcriptional regulation of *BnaC04.MYC70* and BnaC02G0181500ZS are activated by *BnaA07.NAC13*. **a** Schematic representation of the constructs used for the dual-luciferase assay. The effector constructs contain *BnaA07.NAC13* driven by the CaMV35S promoter. The reporter construct contains the firefly luciferase driven by *BnaC04.MYC70* and BnaC02G0181500ZS promoter, and the Renilla luciferase (REN) driven by the CaMV35S promoter. And the black square is the terminator. **b,c** Bar graph showing the relative LUC/REN ratio in the dual-luciferase assay. Values are means(SD) (n = 3 biological repeats).

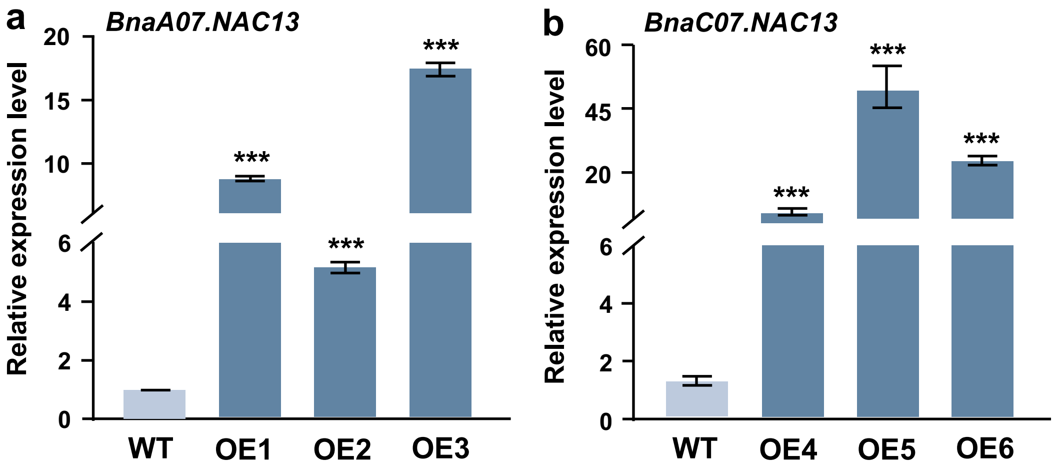

Fig. S27 Expression of *BnaA07.NAC13* and *BnaC07.NAC13* in WT and OE lines analyzed by quantitative RT-PCR using RNA extracted from leaves. (a, b) Values are means(SD) (n = 3 biological repeats). *** indicates $P < 0.001$ compared with WT in Student's *t* test.

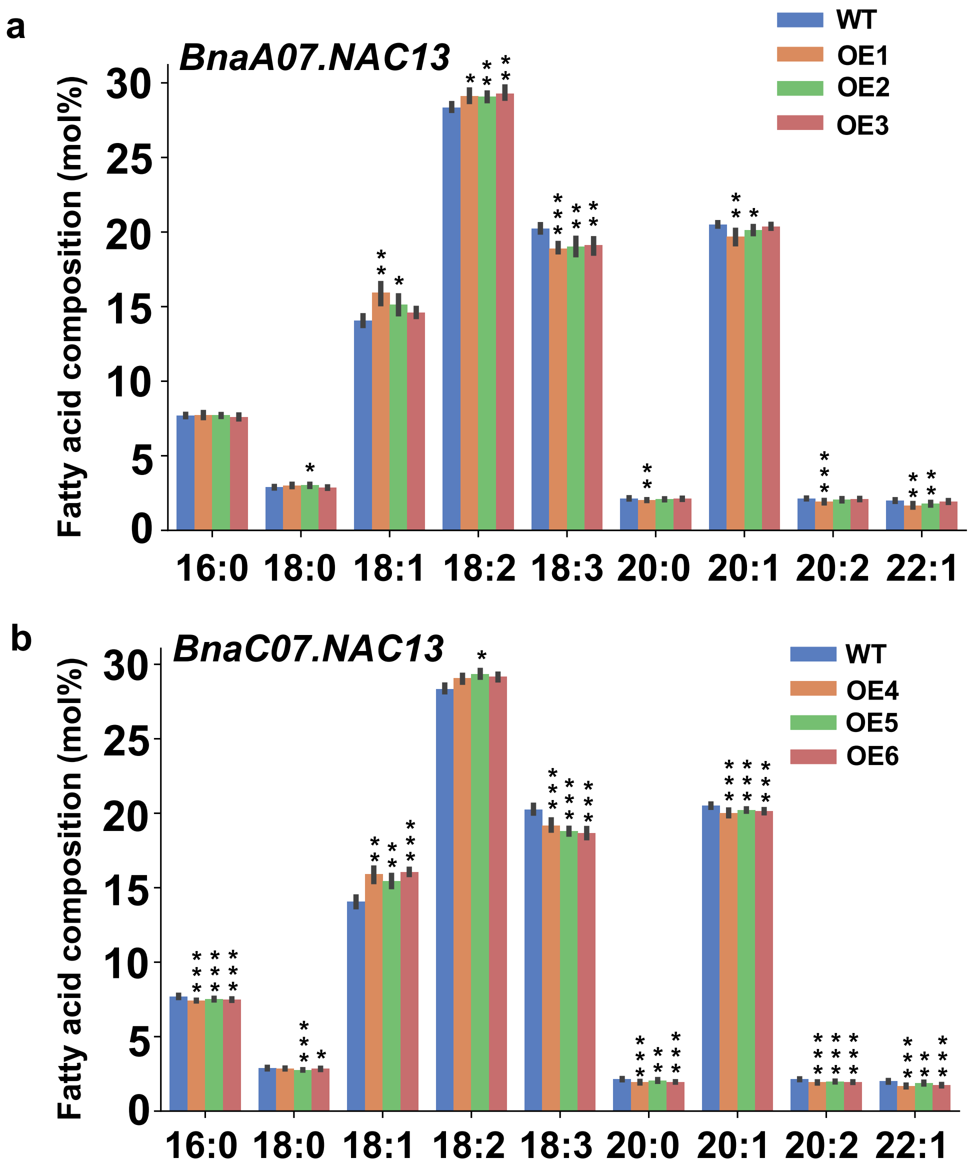

Fig. S28 Fatty acid composition phenotype of OE lines (*BnaA07.NAC13*, *BnaC07.NAC13*). (a, b) Values are means(SD) (n = 5 biological repeats). * indicates $P < 0.05$, ** indicates $P < 0.01$ and *** indicates $P < 0.001$ compared with WT in Student's t test.

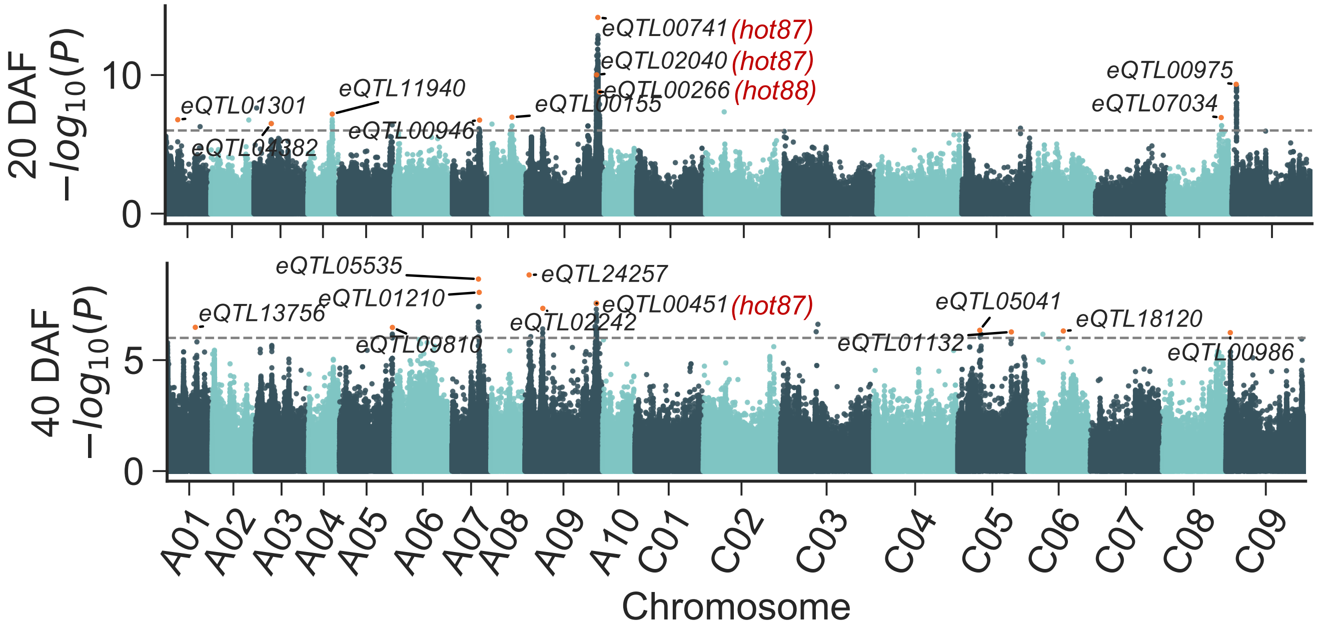

Fig. S29 Manhattan plot *of BnaA07.NAC13* eGWAS at 20 DAF and 40 DAF.

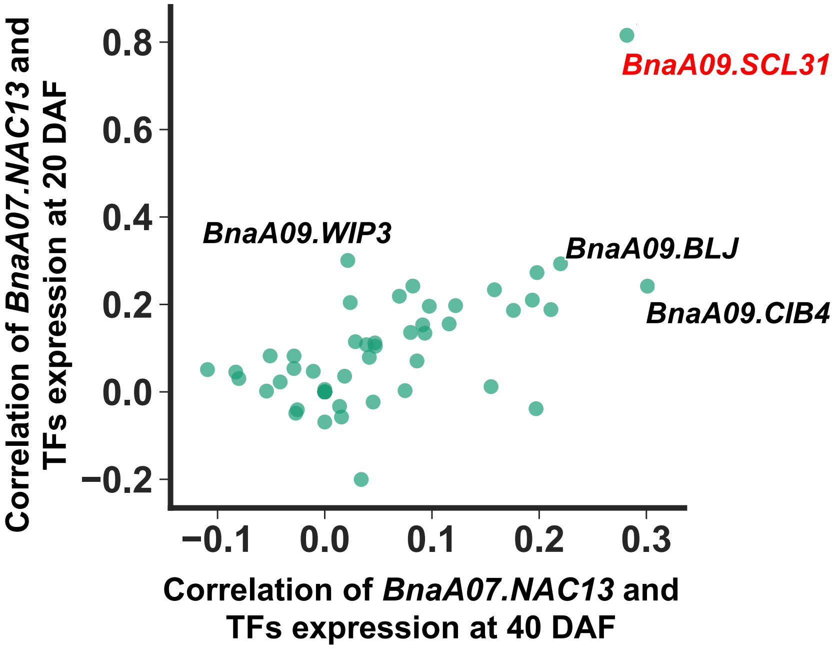

Fig. S30 Correlation between expression levels of *BnaA07.NAC13* and TFs (such as *BnaA09.SCL31* , *WIP DOMAIN PROTEIN 3* (*BnaA09.WIP3*), *CRY2-INTERACTING BHLH 4* (*BnaA09.CIB4*), *BLUEJAY* (*BnaA09.BLJ*)) in Hotspot87-88 at 20 DAF and 40 DAF.

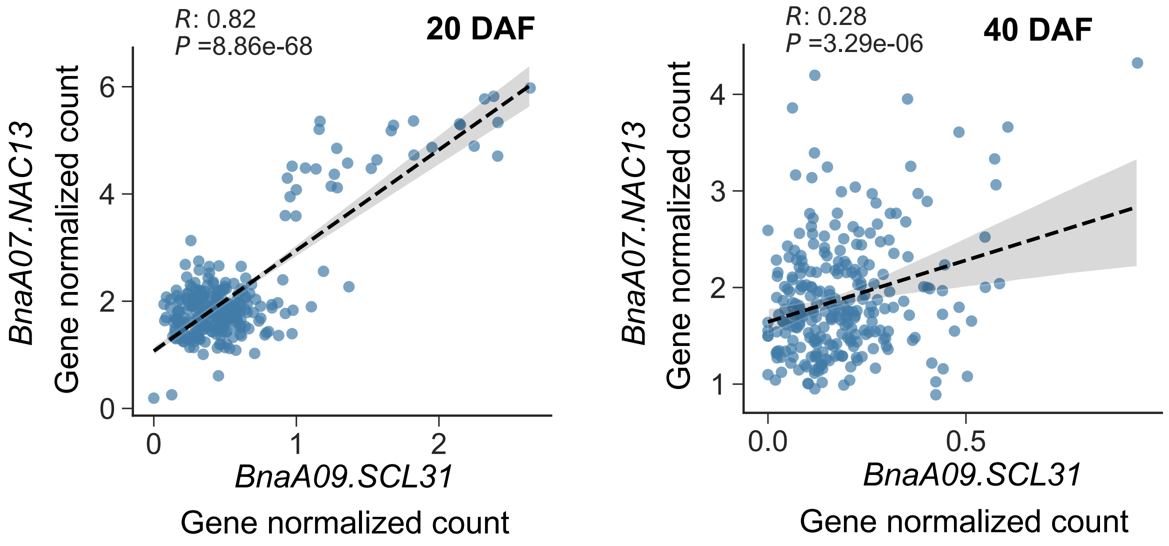

Fig. S31 Correlation between expression levels of *BnaA09.SCL31* and *BnaA07.NAC13* at 20 DAF and 40 DAF.

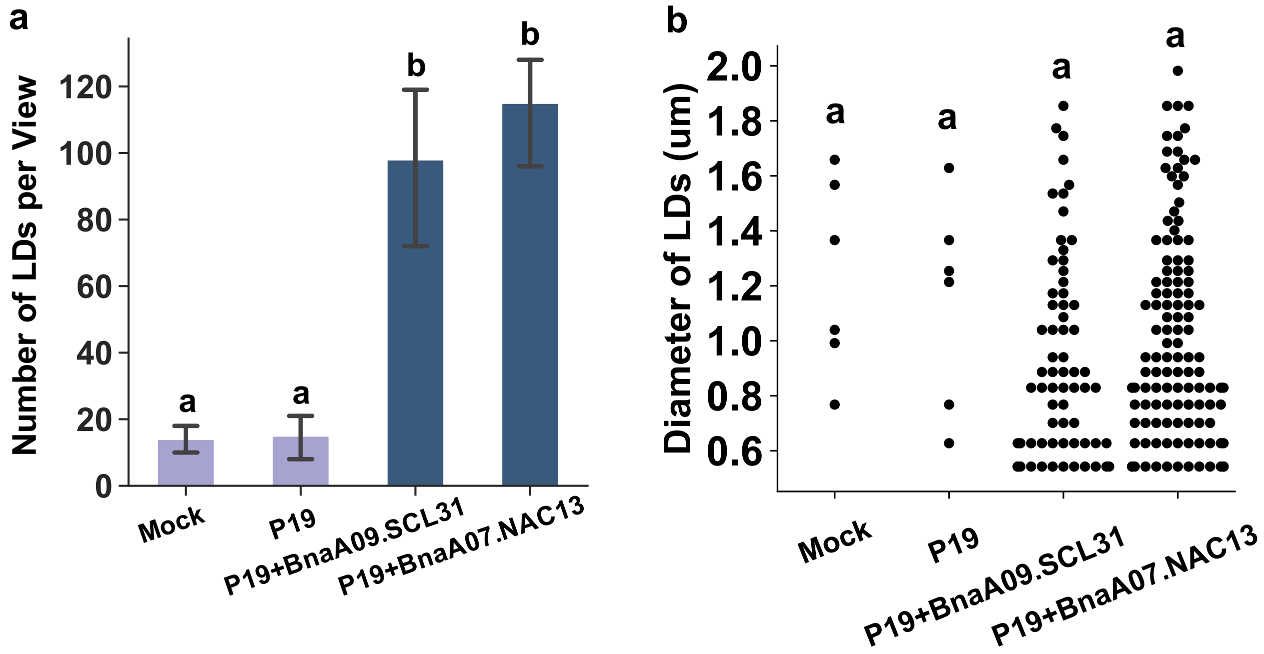

Fig. S32 Size and number of LDs expressing BnaA09.SCL31 and BnaA07.NAC13s in tobacco leaves. **a** LD count expressing BnaA09.SCL31 and BnaA07.NAC13s by size (average diameter). Values are means(SD) (n = 3 biological repeats). Different letters indicate significant difference at $P < 0.05$, as determined by one-way ANOVA with Tukey's post-test. **b** Quantification of LD sizes in leaf mesophyll cells. Circles represent size of LDs.

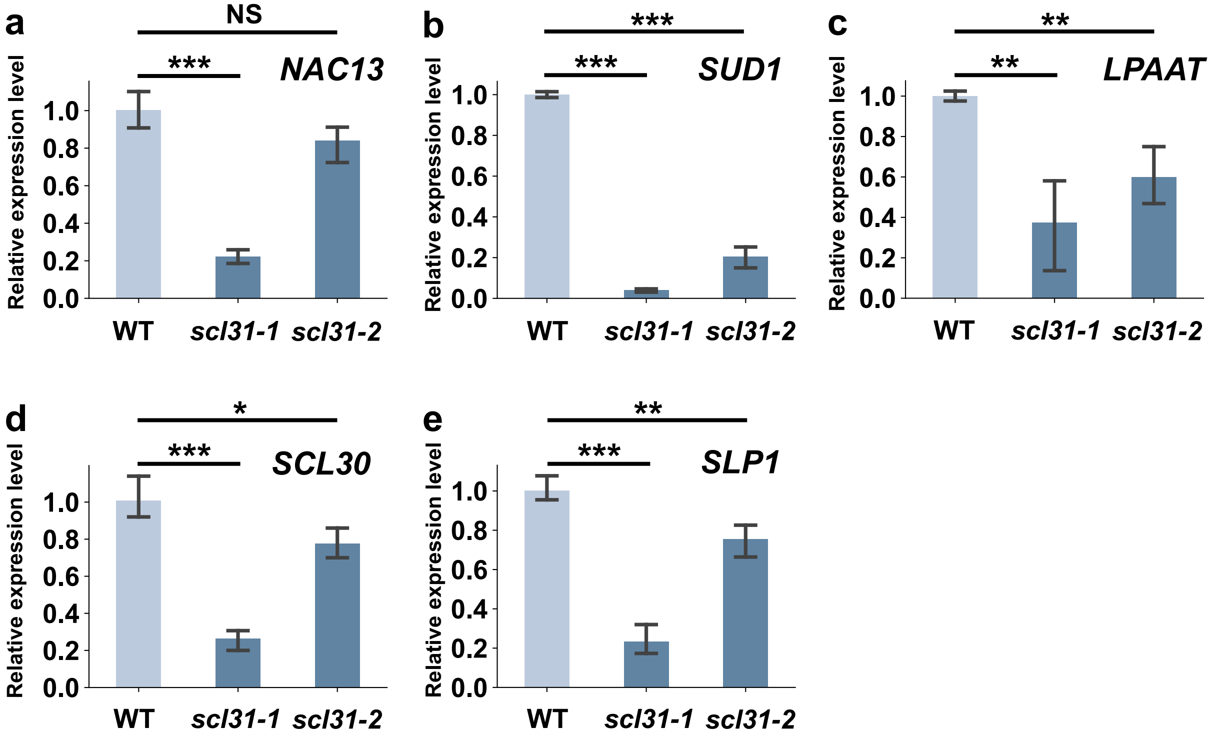

Fig. S33 Expression of *NAC13, SUD1, LPAAT, SCL30, SLP1* in WT, *scl31-1*, *scl31-2* analyzed by quantitative RT-PCR using RNA extracted from leaves. (a-e) Values are means(SD) (n = 3 biological repeats). "NS" indicates $P > 0.05$, * indicates $P < 0.05$, ** indicates $P < 0.01$ and *** indicates $P < 0.001$ compared with WT in Student's *t* test.

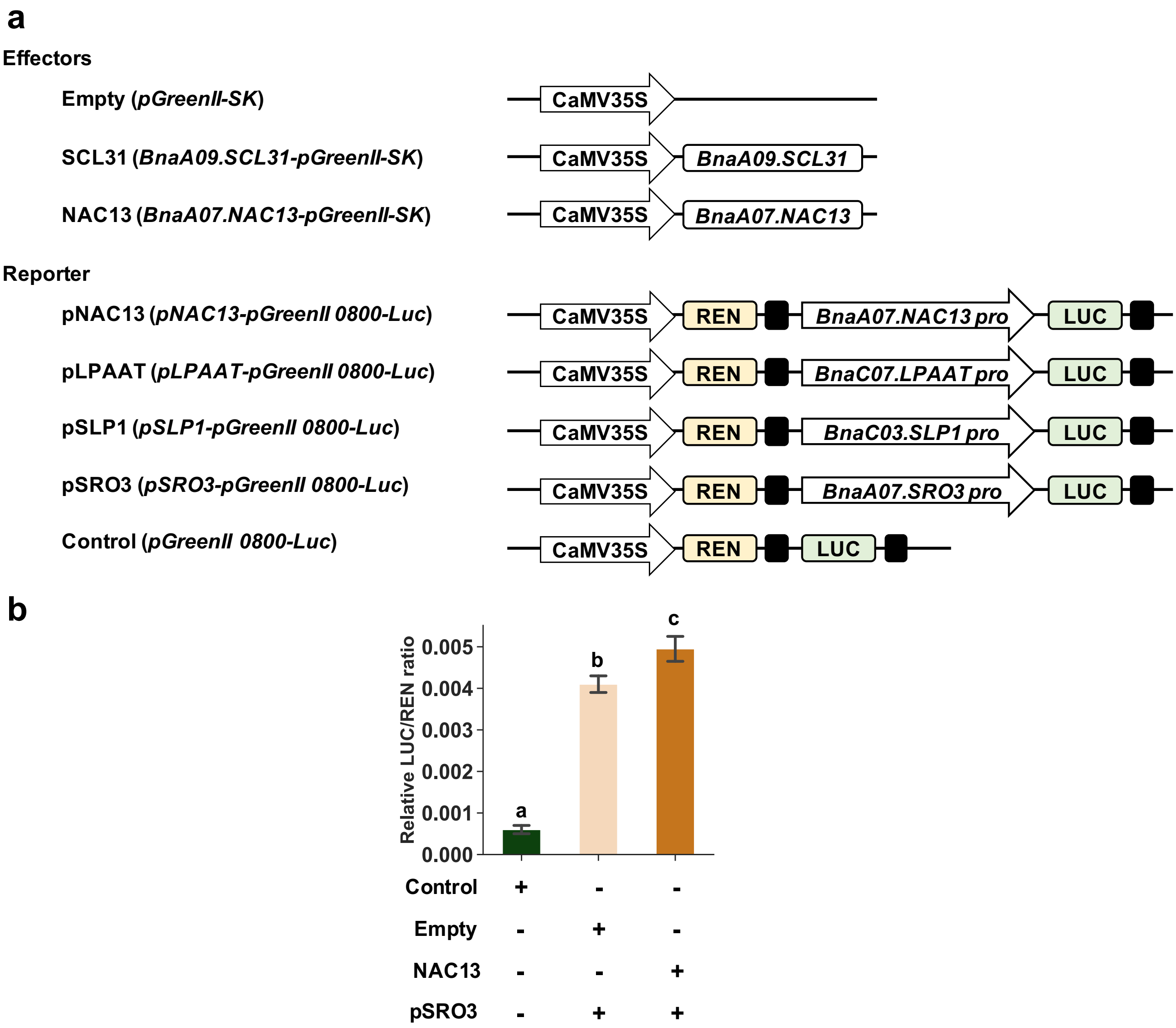

Fig. S34 Transcriptional regulation of genes are activated by *BnaA09.SCL31* or *BnaA07.NAC13*. **a** Schematic representation of the constructs used for the dual-luciferase assay. The effector constructs contain *BnaA09.SCL31* and *BnaA07.NAC13* driven by the CaMV35S promoter, respectively. The reporter construct contains the firefly luciferase driven by *BnaA07.NAC13*, *BnaC07.LPAAT*, *BnaC03.SLP1* and *BnaA07.SRO3* promoter, and the Renilla luciferase (REN) driven by the CaMV35S promoter. And the black square is the terminator. **b** Bar graph showing the relative LUC/REN ratio in the dual-luciferase assay. Values are means(SD) (n = 3 biological repeats). *BnaA07.SRO3* (*SIMILAR TO RCD ONE 3*)
