# Supplementary material for: Comprehensive transcriptional variability analysis reveals gene networks regulating seed oil content of Brassica napus
Source: Genome Biol. 2022 Nov 7;23:233. doi: 10.1186/s13059-022-02801-z (PMC9639296; doi:10.1186/s13059-022-02801-z)
Supplement: Supplementary file 3 — Additional file 3. Review history. [file 13059_2022_2801_MOESM3_ESM.docx]

Review History

**First round of review**

**Reviewer 1**

**Were you able to assess all statistics in the manuscript, including the appropriateness of statistical tests used?**

Yes, and I have assessed the statistics in my report.

**Comments to author:**

Tan et al. provides a global landscape of gene networks regulating seed oil content in Brassica napus using comprehensive transcriptional variability analysis. The results obtained in this study give some insights into the complicated gene networks of seed oil synthesis genome-wide. However, there are still some serious concerns to be addressed before being acceptable for publication.

1. The seed oil content (SOC) of Brassica napus using genome- and transcriptome-wide association studies with 505 accessions has been reported as cited in the reference [47]. In this manuscript of line107-129, the authors employed a different reference genome to reanalyze this phenotype. I am most concerned about the rationale why the authors used another reference genome for re-analysis, and what is the difference between this result and the previous one?

2. In line 210-212, if this finding has already been reported before [47], it is not a new one for this study. Actually, differential homeolog gene expressions between sub-genomes are ubiquitous and well documented in many previous studies of allopolyploidy crops. The authors should introduce the relevant reference articles in the background and discussion sections.

3. In line 296-302, the authors found that knock-out of SCL31 in Arabidopsis decreased the SOC and affected silique length and seed sizes as well., and stated that SCL31 play an important role in seed oil content. It is quite possible that SCL31 reduces seed oil content because of its adverse effect on seed development. In line 294 to 295, the authors made a statement that "gene expression of BnaA09.SCL31 was significantly positively correlated with SOC". Is the gene expression of BnaA09.SCL31 correlated with silique length and seed size? If not, how to explain other phenotypic differences in scl31 mutant in Arabidopsis? If it is caused by species differences, authors should perform functional verification in Brassica napus.

4. In line 318-320, the authors found eight genes with NAC motif in the promoters were highly correlated with the expression of BnaA07.NAC13 and BnaC07.NAC13 by deep learning algorithms. What are the eight specific genes? Authors should conduct additional molecular experiments such as EMSA and LUC/REN approaches to validate the reliability of Basenji model here in predicting genes associated with SOC, in particular for new candidate genes.

**Reviewer 2**

**Were you able to assess all statistics in the manuscript, including the appropriateness of statistical tests used?**

Yes, and I have assessed the statistics in my report.

**Comments to author:**

In the study, taking the Brassica napus as an example, the author performed an experiment to reveal gene networks regulating seed oil content using multiple methods, such as machine learning models and deep learning models, which have certain novelty.

(1) Regarding the role of eQTL in the discovery of excellent genes, the author described too little in the first paragraph of Introduction, please added some new reference, for example in other crops, what type of key variation is and how many distances are there from key genes, as well as what is the detailed regulation mechanism between eQTLs and genes.

(2) Line90, it cannot be considered that the regulatory network is unclear because of the complex genome. I think the former exists forever, while the latter could become clearer. Therefore, please added the detailed disadvantage of current progress.

(3) Line 110, the author performed transcriptome sequencing at 20 and 40 days after flowering. Why did they choose these two periods?

(4) Line 121, 38509 should be deleted.

(5) Line 135, at 40 DAF, what is the eQTL of WRI1 regulated, can you speculate on the possible physiological reasons.

(6) Line260-270, the author only selected SOC and SGC for TWAS analysis, I suggest that it may be more meaningful to compare eQTL hotspots with all previous QTLs for seed-related traits, such as appearance traits. Of course, after the comparison, you can select SOC and SGC to do subsequent analysis.

(7) Line378, The author mentioned that Studying the imbalance characteristics of polyploid genomes can be a key tool to resolve the regulatory mechanisms of polyploid crop evolution, domestication and improvement. However, when analyzing SOC-related regulatory mechanisms in line303-line360, whether was the above theory about the imbalance of the characteristics of polyploid genomes existed. Wouldn't it be better if they could be combined organically?

(8) Line 475, some numbers were not used in scientific notation? Please check it in the all manuscript.

(9) Line 482, 377,616 and 373,073, which period is it?

(10) Line486, why you select 500 kb to discriminate Local eQTL and distant eQTL, is it referring to LD decay? It should be pointed out.

(11) In the Data availability section, the reference number of the previous literature should be listed.

**Reviewer 3**

**Were you able to assess all statistics in the manuscript, including the appropriateness of statistical tests used?**

Yes, and I have assessed the statistics in my report.

**Comments to author:**

This study examined eQTLs for genes expressed at 20 and 40 DAF in Brassica napus using previously published resequencing and transcriptome datasets. Fifty-nine ATAC-seq data were generated, with peaks enriched for some eQTLs. An eQTL hotspot co-localized with a previously detected QTL for seed oil content (SOC). Further integration of ATAC and transcriptome data was used to detect SOC-associated TFs. The main problem with this study is that the major conclusions jump from the results without solid experimental validation, or confuse cause and effect. Furthermore, the first part of eQTL detection and the second part of TF identification are like two relatively independent artifacts pieced together. Although there is an eQTL hotspot near SCL13, no solid evidence was provided.

Major

1. Population data usually have high noise, mainly caused by mapping and SNP detection errors, because the population genomes are different from the reference genome. Polyploid genomes in particular, with two sets of similar genomes, have much higher errors in mapping and SNP calling. Different software and different parameters can lead to completely different results. Experimental verification is definitely required. However, the experimental validation of this study is far from convincing.

1) Given that eQTL is the main outcome of the manuscript, at least one eQTL site needs to be validated for its regulation of SOC by gene editing.

2) To validate the top TF in regulating SOC, using Arabidopsis alone is not convincing. Genetic manipulation in Brassica napus is needed to confirm the effect of TF on ROC.

3) It is inferred that hotspots 87-88 may affect BnaA09.SCL31 and thus affect SOC. Figure 4e shows that different haplotypes in the SLC31 promoter are associated with different levels of SOC, but the y-axis is not 0-based and the differences appear to be small, all appearing to be between 46.5-47. In addition, the expression distribution of SCL31 needs to be shown to demonstrate whether its expression is controlled by the corresponding haplotype and correlated with SOC.

2. . Lines 210-212. Cause and effect are mixed up. The higher proportion of eQTLs in An is likely due to the higher proportion of SNVs in An, possibly caused by the high diversity of An subgenomes in the population. Therefore, the unbalanced eQTL number reflects only the unbalanced SNV number, rather than drawing the conclusion that "regulation between subgenomes is unbalanced between An and Cn subgenomes". Likewise, subsequent expression analysis related to this conclusion do not hold.

3. line：216-217 "We assumed that if a gene was in the distant eQTL of its homoeologous gene, then it was likely to regulate the homoeologous gene. " The 'regulate' here is arbitrary, since it is difficult to speculate the cause and effect based on the association. Figure 3 based on this hypothesis without experimental validation for even one site is not convincing.

4. Lines 228-249. Direct regulation between homologous genes is an interesting result, however, eQTL results are more likely to be affected by noise introduced during mapping and snp calling, especially for population data whose genome sequence differs from the reference, and in polyploid with two sets of similar genomes. Without experimental evidence, the results are not convincing.

5. The eQTLs were detected using previously published resequencing and transcriptome. ATAC-seq data were primarily used to validate results. Predicting TF using ATAC data may be a separate study. Despite the presence of an eQTL hotsplot in TF, there is no experimental evidence to support this regulatory relationship.

Minor:

In view of the detection of eQTL in populations, especially polyploid populations, a large amount of noise can be introduced from mapping and snp calling, and different protocols and parameters may lead to completely different results. However, not only the details of the analyses are not described in the text, but some important intermediate results are not introduced when they are used.

1. The results and methods sections do not clearly describe how local and distant eQTL are defined?

2. Fig.1c. The legend and the main text do not make it clear how the explanation rate is calculated

3. There are several numbers for different accessions in ATAC-seq. What materials do the different numbers represent? figS5 labels 22, 26, 34, and 40 instead of 20, 26, 34, and 40 as described in the text.

4. Line158 mentioned that the lead SNPs of eQTL were identified in the previous article, but there was no relevant description in the previous article, and no article containing the identification method was cited, so how were the lead SNPs identified?

5. Fig. 2a,.Where is the blue histogram mentioned in the legend.

6. Fig. S15. What do the x-axis and y-axis on the right panel represent?

**Authors Response**

**Point-by-point responses to the reviewers’ comments:**

Reviewer #1:

Tan et al. provides a global landscape of gene networks regulating seed oil content in Brassica napus using comprehensive transcriptional variability analysis. The results obtained in this study give some insights into the complicated gene networks of seed oil synthesis genome-wide. However, there are still some serious concerns to be addressed before being acceptable for publication.

*Response: We are grateful to the reviewer for the positive comments on our study.*

1. The seed oil content (SOC) of Brassica napus using genome- and transcriptome-wide association studies with 505 accessions has been reported as cited in the reference [47]. In this manuscript of line107-129, the authors employed a different reference genome to reanalyze this phenotype. I am most concerned about the rationale why the authors used another reference genome for re-analysis, and what is the difference between this result and the previous one?

*Response: Thanks for your comment. The first B. napus reference genome 'Darmor-bzh' was published in 2014 and assembled using Sanger + Illumina + Titanium GSFLX 454 technology, which was largely incomplete due to limitations in available read length at that time [1]. So you can see that in the study for SOC [2], we specifically developed a LD-based approach to place variants on random contigs onto rapeseed chromosomes (you can see the detailed information in the Method section of the study). To fundamentally address this issue, our lab constructed a higher quality reference genome of rapeseed cv. ZS11 by Illumina + PacBio + HiC + BioNano assembly strategy, which significantly improved the contigN50, scaffold N50 compared to Darmor (Darmor genome size: 849Mb, Contig N50: 39Kb; ZS11 genome size: 1008Mb, Contig N50: 159Kb) and reduced the number of reduced random contigs [3].*

*In our SOC study, the high-quality reference ZS11 genome is not available yet and we used the genome of Darmor-bzh. After we released the ZS11 genome, it is a reasonable choice to use ZS11 genome in this study. We have not done the comprehensive eQTL and related analysis using the old genome as reference.*

2. In line 210-212, if this finding has already been reported before [47], it is not a new one for this study. Actually, differential homeolog gene expressions between sub-genomes are ubiquitous and well documented in many previous studies of allopolyploidy crops. The authors should introduce the relevant reference articles in the background and discussion sections.

*Response: Thank you for the suggestion. We agree that differential homeolog gene expressions between sub-genomes are well documented in allopolyploidy crops. But these reports are mainly at the gene expression level. In our study, we compared the eQTL between two subgenomes, which reflected the unbalanced regulation between two subgeomes. And this different from comparing the gene expression level between two subgenomes.*

*According to your advice, we have revised the Introduction (Lines 54-63) and Discussion (Lines 403-408) sections as follows “The overall gene expression of the cotton Dn subgenome was stronger than An subgenome, and the bias of DNA methylation levels can influence the expression bias of homologous gene pairs in the subgenomes [16, 17]. Moreover, B. napus gene expression levels and various active epigenetic signal modifications were significantly stronger on An subgenome than on Cn subgenome, with a higher proportion of SNPs were also observed on An subgenome [11, 18]. eQTL is also a good approach to explore the characteristics and regulatory mechanisms among subgenomes of polyploid plants [19-21]. In cotton, for example, eQTL analysis revealed the imbalanced genetic regulation patterns between An and Dn subgenomes and indicated the important role of the Dn subgenome in fiber development [19].” and “In the past studies, previous authors applied epigenome mapping [11] and GWAS [70] of B. napus to reveal imbalanced transcription and different evolutionary trends among subgenomes, indicating that the overall gene expression and activity epigenetic signal modification levels were significantly stronger on An subgenome than Cn subgenome, and the ratio of expression bias and epigenetic activity bias of homologous gene pairs on An and Cn is symmetric [11, 71].”*

3. In line 296-302, the authors found that knock-out of SCL31 in Arabidopsis decreased the SOC and affected silique length and seed sizes as well., and stated that SCL31 play an important role in seed oil content. It is quite possible that SCL31 reduces seed oil content because of its adverse effect on seed development. In line 294 to 295, the authors made a statement that "gene expression of BnaA09.SCL31 was significantly positively correlated with SOC". Is the gene expression of BnaA09.SCL31 correlated with silique length and seed size? If not, how to explain other phenotypic differences in scl31 mutant in Arabidopsis? If it is caused by species differences, authors should perform functional verification in Brassica napus.

*Response: Thanks for your valuable suggestions, we fully agree with your comments, SCL31 may ultimately affect the seed oil content by influencing the seed development process. At present, we have only collected thousand seed weight (TSW) of the population (unpublished), so we investigated the correlation between SCL31 haplotypes, expression levels and TSW by association analysis. We constructed haplotypes from variants in the SCL31 gene region and the 2-kb upstream region, and the results showed significant differences in TSW corresponding to the various haplotypes (PTSW = 3.0×10-6; Fig. R1). We also found that gene expression of BnaA09.SCL31 in 40 DAF was positively correlated with TSW (Pearson’s correlation coefficient [PCC] R40 DAF-TSW = 0.18, P40 DAF-TSW = 4.04×10-3). These results suggest that SCL31 is likely to be involved in the regulation of rapeseed seed development. Because we have another project on the TSW and the TSW data has not been released. We choose not to include the results in this study.*

*To further confirm that BnaA09.SCL31 is regulating the lipid accumulation. We transiently expressed BnaA09.SCL31 and BnaA07.NAC13 in tobacco leaves and the influence on lipid droplets (LDs) was examined by confocal microscopy (this experiment is commonly used in lipid metabolism field to prove if a candidate gene could increase oil accumulation. Oil is stored in the LD and more LD indicates more oil accumulation). As shown in Fig. R2 and Fig. R3, BnaA09.SCL31 and BnaA07.NAC13 elevated the numbers of LDs in leaf cells. This new experiment provided more evidence that BnaA07.NAC13 and BnaA09.SCL31 have a regulatory effect on lipid accumulation. We have added relevant experiments in the manuscript (Lines 358-363, Figure 5d and Fig. S32). We described results in the manuscript as following:*

*“To provide solid evidence, we transiently expressed BnaA09.SCL31 and BnaA07.NAC13 in tobacco leaves. Examination by confocal microscopy revealed that BnaA09.SCL31 and BnaA07.NAC13 elevated the numbers of lipid droplets (LDs) in leaf cells (Fig. 5d; Additional file 1: Fig. S31). This indicates that BnaA07.NAC13 and BnaA09.SCL31 have a direct effect on lipid accumulation.”*

*Fig. R1 Box plots for TSW based on the haplotypes of variations in gene region and upstream 2 kb region of BnaA09.SCL31.*

*Fig. R2 Representative confocal images of LDs (BODIPY 493/503 fluorescence, false color red) in tobacco leaves. Blue color shows chloroplast auto- fluorescence. Images were collected at the same magnification. Mock infection was infiltrated with media only; P19 was used as a viral suppressor of transgene silencing and was co-expressed in all BnaA09.SCL31 and BnaA07.NAC13 treatments. Bar = 20 μm.*

*Fig. R3 Size and number of LDs expressing BnaA09.SCL31 and BnaA07.NAC13s in tobacco leaves. a LD count expressing BnaA09.SCL31 and BnaA07.NAC13s by size (average diameter). Values are averages and SD of three individual experiments (with three images from each replicate). Different letters indicate significant difference at P < 0.05, as determined by one-way ANOVA with Tukey’s post-test. b Quantification of LD sizes in leaf mesophyll cells. Circles represent size of LDs.*

*Prior to our study, there was no report on the regulatory mechanism of SCL31. Both Brassica napus and Arabidopsis belong to Brassicaceae. Plenty of evidence proved that conserved gene functions between Brassica napus and Arabidopsis. In this study, we added a discussion of the molecular mechanism of SCL31 regulation of seed oil content in the Discussion section (Lines 432-438). In fact, we also designed a series of experiments including transgenic experiments to investigate the function of this gene in rapeseed, and we hope that these experiments will further help our understanding of the molecular regulation mechanism of SCL31 in our subsequent studies. However, as you know, acquiring of gene-edited materials and evaluated the phenotype in the field need 2-3 years.*

*“In the SCL31 mutants, in addition to a significant decrease in SOC, shorter siliques and smaller seeds were also observed. We also found BnaA09.SCL31 haplotypes were significantly associated with thousand seed weight (TSW; P = 3.0×10-6), and expression of BnaA09.SCL31 in 40 DAF was positively correlated with TSW (PCC R40 DAF-TSW = 0.18, P40 DAF-TSW = 4.04×10-3). These results suggest that SCL31 may also play a pleiotropic role in plant growth and development besides regulating SOC (Fig. 4f, g; Additional file 1: Fig. S22)”.*

4. In line 318-320, the authors found eight genes with NAC motif in the promoters were highly correlated with the expression of BnaA07.NAC13 and BnaC07.NAC13 by deep learning algorithms. What are the eight specific genes? Authors should conduct additional molecular experiments such as EMSA and LUC/REN approaches to validate the reliability of Basenji model here in predicting genes associated with SOC, in particular for new candidate genes.

*Response: We apologize for this confusing statement. In addition to the homologs of NAC13 itself (BnaA07.NAC13 and BnaC07.NAC13), there are actually six genes (with NAC motif in the promoter region of the gene) that are highly correlated with the expression of BnaA07.NAC13 and BnaC07.NAC13. These six genes are BnaC06.GLTP, BnaA06.RIN4, BnaA04.MYC70, BnaC04.MYC70, BnaC04.PBL30 and BnaC02G0181500ZS. We have corrected the sentence as follows “Subsequently, we integrated the eQTL results of TWAS significant genes of SOC and selected six genes (BnaC06.GLTP, BnaA06.RIN4, BnaA04.MYC70, BnaC04.MYC70, BnaC04.PBL30) whose eQTL interval harbor NAC13 and had NAC motif in its promoter, and we also found that the expression of these genes was highly correlated with BnaA07.NAC13 and BnaC07.NAC13 (PCC R from 0.55 to 0.8; Additional file 1: Fig. S25).” (Lines 334-339).*

*The Basenji model is used to discover local patterns in sequence data that may represent sequence features, such as cis-elements. After identification of cis-regulatory motifs，we used Tomtom software to compare the similarity with known motifs. The genes which have NAC motif in promoter region and whose expression significantly associated with NAC13 were considered to be more likely regulated by two NAC13 genes.*

*According to your suggestion, we performed transient dual-luciferase assay to verify the results and found that BnaC04.MYC70 and BnaC02G0181500ZS are activated by BnaA07.NAC13 (Fig. R4). We have added relevant experiments and the description in the text Lines 339-342 and Fig. S26.*

*“To further confirm the prediction, we performed transient dual-luciferase assay to verify the results and found that BnaC04.MYC70 and BnaC02G0181500ZS were activated by BnaA07.NAC13.”*

*Fig. R4 Transcriptional regulation of BnaC04.MYC70 and BnaC02G0181500ZS are activated by BnaA07.NAC13. a Schematic representation of the constructs used for the dual-luciferase assay. The effector constructs contain BnaA07.NAC13 driven by the CaMV35S promoter. The reporter construct contains the firefly luciferase driven by BnaC04.MYC70 and BnaC02G0181500ZS promoter, and the Renilla luciferase (REN) driven by the CaMV35S promoter. And the black square is the terminator. b,c Bar graph showing the relative LUC/REN ratio in the dual-luciferase assay. Values are means (SD; n = 3 biological repeats).*

Reviewer #2:

In the study, taking the Brassica napus as an example, the author performed an experiment to reveal gene networks regulating seed oil content using multiple methods, such as machine learning models and deep learning models, which have certain novelty.

*Response: We are honored that the review considers our work to be innovative.*

(1) Regarding the role of eQTL in the discovery of excellent genes, the author described too little in the first paragraph of Introduction, please added some new reference, for example in other crops, what type of key variation is and how many distances are there from key genes, as well as what is the detailed regulation mechanism between eQTLs and genes.

*Response: Thanks for the suggestion, we have already added the corresponding content in Lines 48-53.*

*“In recent years, this approach has also played an important role in the resolution of key genes and regulatory networks for plant traits, such as resolving that Abscisic acid 8′-hydroxylase plays a negative role in maize drought tolerance [5], GhHRK1 is a negative regulator in cotton high temperature stress [6] and Aradu.10025440 was identified as a novel gene that may control the peanut purple testa [7].”*

(2) Line90, it cannot be considered that the regulatory network is unclear because of the complex genome. I think the former exists forever, while the latter could become clearer. Therefore, please added the detailed disadvantage of current progress.

*Response: Thank you and we total agree with your point. we have further revised the content as follows “There are complex regulatory networks between B. napus subgenomes [52, 53], previous studies lacked the analysis of regulatory networks using the omics data at population level, and prediction methods of regulatory networks also need to be further developed in the face of new high-throughput sequencing technologies. eQTL mapping from rapeseed population, epigenetic landscapes constructed by ChIP-seq and ATAC-seq [54], and the well-established machine learning prediction models, can help explore the key TFs and corresponding gene regulatory networks involved in seed oil synthesis” (Lines 96-103).*

(3) Line 110, the author performed transcriptome sequencing at 20 and 40 days after flowering. Why did they choose these two periods?

*Response: 20 DAF and 40 DAF are two representative periods of early and late oil biosynthesis in rapeseeds. Seed oil biosynthesis starts approximately at 20 DAF and accumulates actively at 40 DAF in B. napus. We have previously published our study on genetic basis of seed oil content also at these two developmental stages [2].*

(4) Line 121, 38509 should be deleted.

*Response: We have deleted it.*

(5) Line 135, at 40 DAF, what is the eQTL of WRI1 regulated, can you speculate on the possible physiological reasons.

*Response: This is a great question. WRI1 can directly increase the expression levels of genes involved in plastid glycolysis and fatty acid biosynthesis pathways [7]. We searched the homologs of WRI1 in B. napus using BnTIR (http://yanglab.hzau.edu.cn/BnTIR). From 20 DAF to 32 DAF, the gene was in a high expression state, and from 32 DAF until full seed maturity, the expression of the gene gradually decreased (Fig. R5). Therefore, we believed that WRI1 plays a relatively weak role at 40 DAF, and nearly no eQTL locus regulating WRI1 was identified at 40 DAF. Previous studies have also shown that WRI1 activates gene expression at the early stage of seed maturation by binding to the promoter regions of fatty acid synthesis-related genes mainly in the pre-synthesis phase [8-10] (Fig. R6). In addition, the transcription factors FUS3, ABI3, LEC1, and LEC2 regulate the expression of WRI1 throughout rapeseed seed development [11, 12]. These transcription factors work together to alter the metabolism of sucrose, allowing more carbon to flow to fatty acids and ultimately more TAG to be synthesized [13]. This is consistent with our eQTL results.*

*Fig. R5. Expression levels of four homologous genes of WRI1 in B. napus (ZS11) seed from 20 DAF to 40 DAF. The y-axis represents the Gene expression levels and the x-axis represents days after flowering (from http://yanglab.hzau.edu.cn/BnTIR).*

*Fig. R6. Model of seed oil accumulation by the master regulator WRI1 and its partners [10].*

(6) Line260-270, the author only selected SOC and SGC for TWAS analysis, I suggest that it may be more meaningful to compare eQTL hotspots with all previous QTLs for seed-related traits, such as appearance traits. Of course, after the comparison, you can select SOC and SGC to do subsequent analysis.

*Response: We strongly agree with the reviewer's comments that integrating more phenotypic information is crucial for the understanding of the regulatory mechanism of eQTL hotspots. Previous QTL mapping studies revealed numerous QTLs related to seed-related traits. But it is quite challenge to make comparison because most previous QTLs are preliminary and the QTL interval is large. We have collected other seed-related traits for this population and we have independent projects on these traits. It would be interesting to compare the QTL and eQTL hotspost using the same population in our future study.*

*We tried our best to add more phenotypic data to increase our understanding of eQTL hotspots. We integrated the just published seed coat content (SCC) data [14] for analysis and the corresponding content has been added to the manuscript (Lines 275-279, Figure 4a and Fig. R7).*

*“The published phenotype data of SOC, seed glucosinolate content (SGC) and seed coat content (SCC) were used to perform TWAS [2, 14, 15] (Additional file 2: Table S13,14). Subsequently, we found that the SOC-, SGC- and SCC-related hotspots were mainly located in An subgenome (Fig. 4a).”*

*Fig. R7. The genomic distribution of distant eQTL hotspots and the enrichment of TWAS significant genes of SOC, SGC and SCC in the hotspots. Dot plots represent the distribution of eQTL (outer : 20 DAF, inner : 40 DAF). And the red and light green dots indicate eQTLs in hotspots at 20 DAF and 40 DAF, respectively. Heatmaps represent enrichment of TWAS significant genes of SOC, SGC or SCC (outer : SOC,mid : SGC ,inner : SCC) in hotspots at 20 DAF and 40 DAF (outer : 20 DAF, inner : 40 DAF). The pentagram indicates the location of Hotspot87-88. The bars indicate the number of hotspots. “Special” indicates the specific hotspot, “Partial” indicates the partially overlapping hotspot, and “Overlap” indicates the fully overlapping hotspot at two stages.*

(7) Line378, The author mentioned that Studying the imbalance characteristics of polyploid genomes can be a key tool to resolve the regulatory mechanisms of polyploid crop evolution, domestication and improvement. However, when analyzing SOC-related regulatory mechanisms in line303-line360, whether was the above theory about the imbalance of the characteristics of polyploid genomes existed. Wouldn't it be better if they could be combined organically?

*Response: Thanks for your suggestion, we have added relevant analysis and the description in the text Line279-283, Fig. S15 and Fig.R8.*

*“SOC-related aHGPs with local eQTLs were able to maintain a partial homoeolog expression dosage, such as ADD5, PDAT1 (Pearson’s correlation coefficient [PCC] RADD5 = -0.75, PCC RPDAT1 = -0.35). However, HGP of WRI1 without local eQTL does not maintain homoeolog expression dosage, showing that expression is highly correlated (PCC RWRI1 = 0.84; Additional file 1: Fig. S15).”*

*Fig. R8 Characterization of SOC-related gene regulation in subgenomic imbalance. a Comparison of SOC-related gene correlations in different types of aHGPs at 20 DAF. "BG" represents all HGPs. "Local" represents aHGPs with local eQTLs and "No Local" represents aHGPs without local eQTLs. b Correlation between expression levels of BnaA02.PDAT1 and BnaC02.PDAT1 at 20 DAF. c Correlation between expression levels of BnaA01.AAD5 and BnaC01.AAD5 at 20 DAF. d Correlation between expression levels of BnaA07.WRI1 and BnaC06.WRI1 at 20 DAF.*

(8) Line 475, some numbers were not used in scientific notation? Please check it in the all manuscript.

*Response: We apologize for this mistake, we have checked the full text and corrected them.*

(9) Line 482, 377,616 and 373,073, which period is it?

*Response: Thanks, we have revised the manuscript as following: “The significance threshold of the association was calculated by the software GEC as 1×10-6 and a total of 377,616 and 373,073 significantly associated SNPs were detected at 20 DAF and 40 DAF, respectively”.*

(10) Line486, why you select 500 kb to discriminate Local eQTL and distant eQTL, is it referring to LD decay? It should be pointed out.

*Response: We apologize for the lack of description here. According to the LD decay results of this population [2], the distance of LD decay (r2) to 0.1 is about 500 kb. We also referred to other eQTL studies [16], and finally chose 500 kb to delineate local eQTL and distant eQTL. We have added the statement to the manuscript as following: “Based on the position of lead SNP in relation to the target genes, if the SNP is located within 500 kb (The distance of LD decay [r2] to 0.1 is about 500 kb) upstream and downstream of the gene, it was considered a local eQTLs, otherwise a distant eQTL” (Lines 521-524).*

(11) In the Data availability section, the reference number of the previous literature should be listed.

*Response: Thanks, we have added the corresponding references in this section.*

Reviewer #3:

This study examined eQTLs for genes expressed at 20 and 40 DAF in Brassica napus using previously published resequencing and transcriptome datasets. Fifty-nine ATAC-seq data were generated, with peaks enriched for some eQTLs. An eQTL hotspot co-localized with a previously detected QTL for seed oil content (SOC). Further integration of ATAC and transcriptome data was used to detect SOC-associated TFs. The main problem with this study is that the major conclusions jump from the results without solid experimental validation, or confuse cause and effect. Furthermore, the first part of eQTL detection and the second part of TF identification are like two relatively independent artifacts pieced together. Although there is an eQTL hotspot near SCL13, no solid evidence was provided.

*Response: Thank you for your comments. This is an omics study. We understand that a lot of predictions have been made in this study and experimental validation is important to support the prediction. This work is mostly about eQTL identification, comparison, eQTL hotspot analysis. Because the transcriptome data were from seeds and these eQTL hotspots should potentially have important regulatory functions on seed-related traits. It is reasonable for us to focus on the key eQTL hotspots which co-localize with SOC QTL after we finish analyzing the eQTLs at the whole genome level. After prediction of candidate genes and regulatory relationships, we performed a few experiments to validate the prediction. Overall, different parts are well connected.*

*Regarding “no solid evidence was provided for SCL31”, we tried to provide as much bioinformatics evidence (Fig 4b, 4c; Fig S19b, S30, S31) and experimental evidence (Fig 4f, 4g, 5-5g; Fig S32a) as possible. Especially SCL31 has very high correlation with SOC at population level (this prediction is statistically convincing because of large scale data).*

Major

1. Population data usually have high noise, mainly caused by mapping and SNP detection errors, because the population genomes are different from the reference genome. Polyploid genomes in particular, with two sets of similar genomes, have much higher errors in mapping and SNP calling. Different software and different parameters can lead to completely different results. Experimental verification is definitely required. However, the experimental validation of this study is far from convincing.

1) Given that eQTL is the main outcome of the manuscript, at least one eQTL site needs to be validated for its regulation of SOC by gene editing.

2) To validate the top TF in regulating SOC, using Arabidopsis alone is not convincing. Genetic manipulation in Brassica napus is needed to confirm the effect of TF on SOC.

3) It is inferred that hotspots 87-88 may affect BnaA09.SCL31 and thus affect SOC. Figure 4e shows that different haplotypes in the SLC31 promoter are associated with different levels of SOC, but the y-axis is not 0-based and the differences appear to be small, all appearing to be between 46.5-47. In addition, the expression distribution of SCL31 needs to be shown to demonstrate whether its expression is controlled by the corresponding haplotype and correlated with SOC.

*Response: We fully understand the reviewer’s concern. In order to reduce the influence of data noise on the analysis results as much as possible and to obtain reliable conclusions, we have made the following efforts in data analysis and experimental validation.*

*For data analysis:*

*Use of higher quality reference genome. This can greatly reduce the proportion of significant signals falling in random contigs and increase the accuracy of QTL identification. You can find the detailed information in the response for Reviwer #1 question 1.*

*Use of best practices workflows to identify genomic variants and employ strict thresholds to filter the noise of data. BWA and GATK are two widely used software whose power and accuracy have been extensively validated in polyploid plant genome studies [17-23]. We also used strict thresholds to ensure the accuracy of variant identification. The ‘mapping quality > 20’ parameter is used to filter out reads that mapped to complex sequences (e.g. highly similar sequences in subgenomes), and the ‘sequencing depth > 50’ parameter is used to filter out regions with low resequencing coverage, which maximizes the accuracy of identifying variants. You can find the detailed information in the Methods section (Lines 482-493).*

*Use of simulation-based method to evaluate the accuracy of transcript abundance estimation for homologous gene sets. To assess the accuracy of transcript abundance estimation by mapping RNA-seq reads to the B. napus genome, we simulated RNA-seq datasets using gene models only from one of the ZS11 subgenome (for example, the An genome) and then used all gene models from the B. napus reference genome to calculate TPM values. Simulation performed for the An genome showed a high level* of *correlation (PCC R = 0.91, N = 41338) between the real values and those estimated. This indicates a high accuracy of transcript abundance estimation for homologous gene sets. We also added this result in the Methods section of manuscript (Lines 507-513).*

*For experimental validation:*

*In the manuscript, we applied two T-DNA insertion Arabidopsis mutants of SCL31 to find that SCL31 is an important TF affecting SOC (Fig. 4f, g; Additional file 1: Fig. S21, 22). And heterologous overexpression of BnaA07.NAC13 and BnaC07.NAC13 was performed in Arabidopsis using the CaMV35S promoter (Fig. 5c).*

*To provide solid evidence, we transiently expressed BnaA09.SCL31 and BnaA07.NAC13 in tobacco leaves and the influence on lipid droplets (LDs) was examined by confocal microscopy. As shown in Fig. R2 and Fig. R3, BnaA09.SCL31 and BnaA07.NAC13 elevated the numbers of LDs in leaf cells. This indicates that BnaA07.NAC13 and BnaA09.SCL31 have a regulatory effect on lipid accumulation.*

*We examined the relative expression of some genes related to lipid metabolism by quantitative RT-PCR in SCL31 mutants. The results showed that the relative expression was significantly decreased (Additional file 1: Fig. S33). The effect of BnaA09.SCL31 and BnaA07.NAC13 on downstream gene promoter activity was measured using transient dual luciferase reporter assays (Fig. 5e-g, Additional file 1: Fig. S34, Fig. R4).*

*Both Brassica napus and Arabidopsis belong to Brassicaceae. Plenty of evidence proved that conserved gene functions between Brassica napus and Arabidopsis. To sum up, based on the above analysis process and the experimental validation results, we believe that the results obtained so far are plausible. We strongly agree with the reviewer's comments that using gene editing can further validate our conclusions, and we have designed gene editing efforts for several genes and these efforts are in progress. We believe that with the availability of gene editing materials we can carry out more studies to clarify the molecular regulatory mechanisms of these genes. However, as you know, access to gene-edited materials are not a short-term task, so we are afraid that it will be difficult to include such result in this manuscript.*

*For Figure 4e, as you can see in our population the SOC of all accessions is distributed between approximately 40% and 55%, and we also represent the SOC of the different haplotypes corresponding to the accessions as points in the graph, so the y-coordinate does not start from 0. Meanwhile, the effect of SCL31 on SOC is already very significant for SOC-regulatory genes in rapeseed. SOC is a complex trait controlled by multiple genes. Functional validation of many SOC-related genes suggested a gene effect of 1-2% on SOC. For example, our previously cloned BnPMT6s has an effect on SOC about 1-2% [2]. Many cloned genes are also shown to impact SOC 1-3% in Arabidopsis.*

*As reviewer suggested, we checked the expression distribution between different haplotypes and found that the expression value of BnaA09.SCL31 corresponding to different haplotypes were significantly different at 20 DAF (P = 5.19×10-3). You can also find this information in manuscript Lines 307-310.*

*“By distinguishing the coding region and the upstream 2 kb promoter of SCL31 into four haplotypes, significant differences were observed between SOC and expression value corresponding to various haplotypes (PSOC = 6.7×10-5; Pexpression = 5.19×10-3; Fig. 4d, e)”*

2. Lines 210-212. Cause and effect are mixed up. The higher proportion of eQTLs in An is likely due to the higher proportion of SNVs in An, possibly caused by the high diversity of An subgenomes in the population. Therefore, the unbalanced eQTL number reflects only the unbalanced SNV number, rather than drawing the conclusion that "regulation between subgenomes is unbalanced between An and Cn subgenomes". Likewise, subsequent expression analysis related to this conclusion do not hold.

*Response: We fully understand the reviewer’s concern. Although we thought that the reviewer’s interpretation for unbalanced regulation between subgenomes was actually similar to ours, that the higher proportion of eQTLs in An possibly caused by the high diversity of An subgenomes in the population, and you can find that also discussed in the Discussion section (Lines 408-410). On the other hand, we believe that the fact that An has more variants does not certainly mean that the An subgenome will harbor more eQTLs. The eQTL analysis is a good way to investigate the unbalanced subgenome transcription regulation and widely used including the study of cotton [24], wheat [25] and rapeseed [26].*

3. line：216-217 "We assumed that if a gene was in the distant eQTL of its homoeologous gene, then it was likely to regulate the homoeologous gene. " The 'regulate' here is arbitrary, since it is difficult to speculate the cause and effect based on the association. Figure 3 based on this hypothesis without experimental validation for even one site is not convincing.

*Response: We fully understand the reviewer’s concern. The aim of eQTL analysis is to study the effect of genomic variation on the regulation of gene expression by measuring the association between gene expression levels and variant genotypes (e.g. AA, AB, BB) in population samples. eQTL refers to the genomic regions where variants affecting gene expression levels are present, and also by extension to the variants themselves that affect gene expression [27, 28]. Variants in statistically significant association are referred to as eQTL. Genes are functional units in biological processes, and genes associated with eQTL are referred to as eGene. eQTL are the cause and eGene are the result of being regulated by eQTL [29-31]. Based on the above theory, we performed statistical analysis and obtained conclusions on the regulatory relationships of homologous genes among subgenomes. Such an analysis is currently widely accepted [24-26]. Since these conclusions were obtained based on statistical analyses at the genome-wide level, it is difficult to verify them experimentally by selecting one or two examples.*

4. Lines 228-249. Direct regulation between homologous genes is an interesting result, however, eQTL results are more likely to be affected by noise introduced during mapping and snp calling, especially for population data whose genome sequence differs from the reference, and in polyploid with two sets of similar genomes. Without experimental evidence, the results are not convincing.

*Response: Thanks for your suggestion. At this stage, we have tried our best to reduce the noise, both in the data analysis process and in the experimental validation (you can refer to our response to your first question for more details). It is worth noting that our conclusions are based on observations and statistics for genome-wide eQTLs, further excluding bias caused by inaccurate identification of some eQTL. As explained for the previous response, these analytical methods have been widely used in the analysis of polyploid plants, and it is difficult to verify them experimentally by selecting one or two examples.*

5. The eQTLs were detected using previously published resequencing and transcriptome. ATAC-seq data were primarily used to validate results. Predicting TF using ATAC data may be a separate study. Despite the presence of an eQTL hotsplot in TF, there is no experimental evidence to support this regulatory relationship.

*Response: Thanks for your suggestion. We have mainly applied ATAC-seq data to construct a Basenji model to identify key cis-regulatory elements in the promoter regions of TWAS significant genes of SOC. Since there will be multiple candidate genes within the eQTL interval, combining the prediction of gene cis-regulatory elements based on ATAC-seq data can better help us accurately identify regulatory relationships. We have described in detail in the Introduction section on how ATAC-seq data can help in the identification of regulatory relationships in eQTL (Lines 64-84 and Lines 96-103).*

*Combined with the eQTL results we predicted that Bna.NAC13 affects SOC by regulating TWAS significant genes of SOC. To further confirm the prediction, we performed transient dual-luciferase assay to verify the results and found that two predicted genes BnaC04.MYC70 and BnaC02G0181500ZS were activated by BnaA07.NAC13 (Additional file 1: Fig. S26 and Fig.R4). We have also added the results to the manuscript (Lines 339-343).*

Minor:

In view of the detection of eQTL in populations, especially polyploid populations, a large amount of noise can be introduced from mapping and snp calling, and different protocols and parameters may lead to completely different results. However, not only the details of the analyses are not described in the text, but some important intermediate results are not introduced when they are used.

*Response: Thanks, you can see the response to comment 1 for the detailed information.*

1. The results and methods sections do not clearly describe how local and distant eQTL are defined?

*Response: According to the LD decay results of this population [2], the distance of LD decay (r2) to 0.1 is about 500 kb. We also referred to other eQTL studies [16], and finally chose 500 kb to delineate local eQTL and distant eQTL. See the revised manuscript (Lines 618-621) for details and refer to our response to the question 10 of the reviewer #2.*

2. Fig.1c. The legend and the main text do not make it clear how the explanation rate is calculated

*Response: Sorry for the confusion. The ‘explanation rate’ should be ‘Explained variance’, we have changed it in the manuscript and Figure. And we added the detailed information in the Methods section (Line 614) as follows “The explained variance (r2) of LeadSNPs was calculated by FaST-LMM v0.3.8”.*

3. There are several numbers for different accessions in ATAC-seq. What materials do the different numbers represent? figS5 labels 22, 26, 34, and 40 instead of 20, 26, 34, and 40 as described in the text.

*Response: Thanks. We have added the corresponding description in Fig. S5 lengend as follows “The samples are named according to "(22, 26, 34 or 40) DAF" + "accession_cellular ploidy (2C, 3C or 4C)" + "biological replicate" format naming.”.*

4. Line158 mentioned that the lead SNPs of eQTL were identified in the previous article, but there was no relevant description in the previous article, and no article containing the identification method was cited, so how were the lead SNPs identified?

*Response: Sorry for the mistake. The lead SNPs of eQTL were identified in this study. We have removed the “perviously identified” in Line167.*

5. Fig. 2a, Where is the blue histogram mentioned in the legend.

*Response: Sorry for the mistake. In Fig 2a, "the blue histogram" should actually be "the blue line", and we have changed the description.*

6. Fig. S15. What do the x-axis and y-axis on the right panel represent?

*Response: Thanks for your question. The x-axis and y-axis represent enrichment of TWAS significant genes of SOC in hotspots at 20 DAF and 40 DAF. We have added the corresponding description in Fig. S16 legend.*

*Reference*

*1. Chalhoub B, Denoeud F, Liu S, Parkin IA, Tang H, Wang X, Chiquet J, Belcram H, Tong C, Samans B: Early allopolyploid evolution in the post-Neolithic Brassica napus oilseed genome. science 2014, 345:950-953.*

*2. Tang S, Zhao H, Lu S, Yu L, Zhang G, Zhang Y, Yang Q-Y, Zhou Y, Wang X, Ma W, et al: Genome- and transcriptome-wide association studies provide insights into the genetic basis of natural variation of seed oil content in Brassica napus. Mol Plant 2021, 14:470-487.*

*3. Song J-M, Guan Z, Hu J, Guo C, Yang Z, Wang S, Liu D, Wang B, Lu S, Zhou R, et al: Eight high-quality genomes reveal pan-genome architecture and ecotype differentiation of Brassica napus. Nature Plants 2020, 6:34-45.*

*4. Liu S, Li C, Wang H, Wang S, Yang S, Liu X, Yan J, Li B, Beatty M, Zastrow-Hayes G: Mapping regulatory variants controlling gene expression in drought response and tolerance in maize. Genome Biol 2020, 21:1-22.*

*5. Ma Y, Min L, Wang J, Li Y, Wu Y, Hu Q, Ding Y, Wang M, Liang Y, Gong Z: A combination of genome‐wide and transcriptome‐wide association studies reveals genetic elements leading to male sterility during high temperature stress in cotton. New Phytol 2021, 231:165-181.*

*6. Huang L, Liu X, Pandey MK, Ren X, Chen H, Xue X, Liu N, Huai D, Chen Y, Zhou X: Genome‐wide expression quantitative trait locus analysis in a recombinant inbred line population for trait dissection in peanut. Plant Biotechnol J 2020, 18:779-790.*

*7. Baud S, Lepiniec L: Regulation of de novo fatty acid synthesis in maturing oilseeds of Arabidopsis. Plant Physiol Biochem 2009, 47:448-455.*

*8. Ruuska SA, Girke T, Benning C, Ohlrogge JB: Contrapuntal Networks of Gene Expression during Arabidopsis Seed Filling[W]. The Plant Cell 2002, 14:1191-1206.*

*9. Cernac A, Benning C: WRINKLED1 encodes an AP2/EREB domain protein involved in the control of storage compound biosynthesis in Arabidopsis. The Plant Journal 2004, 40:575-585.*

*10. Sun T: Unexpected Role of a TCP Transcription Factor in Seed Oil Biosynthesis. Plant Physiology 2020, 184:550-551.*

*11. Wang H, Guo J, Lambert KN, Lin Y: Developmental control of Arabidopsis seed oil biosynthesis. Planta 2007, 226:773-783.*

*12. Santos‐Mendoza M, Dubreucq B, Baud S, Parcy F, Caboche M, Lepiniec L: Deciphering gene regulatory networks that control seed development and maturation in Arabidopsis. The Plant Journal 2008, 54:608-620.*

*13. Manan S, Chen B, She G, Wan X, Zhao J: Transport and transcriptional regulation of oil production in plants. Critical reviews in biotechnology 2017, 37:641-655.*

*14. Zhang Y, Zhang H, Zhao H, Xia Y, Zheng X, Fan R, Tan Z, Duan C, Fu Y, Li L, et al: Multi-omics analysis dissects the genetic architecture of seed coat content in Brassica napus. Genome Biol 2022, 23:86.*

*15. Tan Z, Xie Z, Dai L, Zhang Y, Zhao H, Tang S, Wan L, Yao X, Guo L, Hong D: Genome- and transcriptome-wide association studies reveal the genetic basis and the breeding history of seed glucosinolate content in Brassica napus. Plant Biotechnol J 2022, 20:211-225.*

*16. Li Z, Wang P, You C, Yu J, Zhang X, Yan F, Ye Z, Shen C, Li B, Guo K: Combined GWAS and eQTL analysis uncovers a genetic regulatory network orchestrating the initiation of secondary cell wall development in cotton. new phytologist 2020, 226:1738-1752.*

*17. Shen C, Wang N, Huang C, Wang M, Zhang X, Lin Z: Population genomics reveals a fine‐scale recombination landscape for genetic improvement of cotton. The Plant Journal 2019, 99:494-505.*

*18. Rimbert H, Darrier B, Navarro J, Kitt J, Choulet F, Leveugle M, Duarte J, Rivière N, Eversole K, Consortium IWGS: High throughput SNP discovery and genotyping in hexaploid wheat. PloS one 2018, 13:e0186329.*

*19. Ling H-Q, Ma B, Shi X, Liu H, Dong L, Sun H, Cao Y, Gao Q, Zheng S, Li Y: Genome sequence of the progenitor of wheat A subgenome Triticum urartu. Nature 2018, 557:424-428.*

*20. Song J-M, Guan Z, Hu J, Guo C, Yang Z, Wang S, Liu D, Wang B, Lu S, Zhou R: Eight high-quality genomes reveal pan-genome architecture and ecotype differentiation of Brassica napus. Nature Plants 2020, 6:34-45.*

*21. Luo H, Wang X, Zhan G, Wei G, Zhou X, Zhao J, Huang L, Kang Z: Genome-wide analysis of simple sequence repeats and efficient development of polymorphic SSR markers based on whole genome re-sequencing of multiple isolates of the wheat stripe rust fungus. PLoS One 2015, 10:e0130362.*

*22. Li L, Zhang C, Huang J, Liu Q, Wei H, Wang H, Liu G, Gu L, Yu S: Genomic analyses reveal the genetic basis of early maturity and identification of loci and candidate genes in upland cotton (Gossypium hirsutum L.). Plant biotechnology journal 2021, 19:109-123.*

*23. Li J, Manghwar H, Sun L, Wang P, Wang G, Sheng H, Zhang J, Liu H, Qin L, Rui H: Whole genome sequencing reveals rare off‐target mutations and considerable inherent genetic or/and somaclonal variations in CRISPR/Cas9‐edited cotton plants. Plant biotechnology journal 2019, 17:858-868.*

*24. Li Z, Wang P, You C, Yu J, Zhang X, Yan F, Ye Z, Shen C, Li B, Guo K, et al: Combined GWAS and eQTL analysis uncovers a genetic regulatory network orchestrating the initiation of secondary cell wall development in cotton. New Phytologist 2020, 226:1738-1752.*

*25. He F, Wang W, Rutter WB, Jordan KW, Ren J, Taagen E, DeWitt N, Sehgal D, Sukumaran S, Dreisigacker S: Genomic variants affecting homoeologous gene expression dosage contribute to agronomic trait variation in allopolyploid wheat. Nature communications 2022, 13:1-15.*

*26. Li R, Jeong K, Davis JT, Kim S, Lee S, Michelmore RW, Kim S, Maloof JN: Integrated QTL and eQTL mapping provides insights and candidate genes for fatty acid composition, flowering time, and growth traits in a F2 population of a novel synthetic allopolyploid Brassica napus. Frontiers in plant science 2018, 9:1632.*

*27. Albert FW, Kruglyak L: The role of regulatory variation in complex traits and disease. Nat Rev Genet 2015, 16:197-212.*

*28. Sul JH, Raj T, De Jong S, De Bakker PI, Raychaudhuri S, Ophoff RA, Stranger BE, Eskin E, Han B: Accurate and fast multiple-testing correction in eQTL studies. The American Journal of Human Genetics 2015, 96:857-868.*

*29. Gilad Y, Rifkin SA, Pritchard JK: Revealing the architecture of gene regulation: the promise of eQTL studies. Trends Genet 2008, 24:408-415.*

*30. Kliebenstein D: Quantitative genomics: analyzing intraspecific variation using global gene expression polymorphisms or eQTLs. Annu Rev Plant Biol 2009, 60:93-114.*

*31. Majewski J, Pastinen T: The study of eQTL variations by RNA-seq: from SNPs to phenotypes. Trends in Genetics 2011, 27:72-79.*

**Second round of review**

**Reviewer 1**

The authors have addressed most of my concerns. I would like to recommend the manuscript to be published.

**Reviewer 2**

No.

**Reviewer 3**

The authors have addressed most of my concerns. I have no further comment.
